# Improved Multi-Strategy Sand Cat Swarm Optimization for Solving Global Optimization

**DOI:** 10.3390/biomimetics9050280

**Published:** 2024-05-08

**Authors:** Kuan Zhang, Yirui He, Yuhang Wang, Changjian Sun

**Affiliations:** 1 College of Information Science and Technology, Northeastern University, Shenyang 110000, China; 20212267@stu.neu.edu.cn (K.Z.); 20212366@stu.neu.edu.cn (Y.H.); 2 School of Aerospace, Harbin Institute of Technology, Harbin 150001, China; 3 School of Software, Henan University, Kaifeng 475001, China; 20210901@henu.edu.cn; 4 College of Electronic Science and Engineering, Jilin University, Changchun 130000, China

**Keywords:** sand cat swarm optimization, fitness–distance balancing strategy, non-exclusive learning search, CEC 2017, metaheuristic algorithm

## Abstract

The sand cat swarm optimization algorithm (SCSO) is a novel metaheuristic algorithm that has been proposed in recent years. The algorithm optimizes the search ability of individuals by mimicking the hunting behavior of sand cat groups in nature, thereby achieving robust optimization performance. It is characterized by few control parameters and simple operation. However, due to the lack of population diversity, SCSO is less efficient in solving complex problems and is prone to fall into local optimization. To address these shortcomings and refine the algorithm’s efficacy, an improved multi-strategy sand cat optimization algorithm (IMSCSO) is proposed in this paper. In IMSCSO, a roulette fitness–distance balancing strategy is used to select codes to replace random agents in the exploration phase and enhance the convergence performance of the algorithm. To bolster population diversity, a novel population perturbation strategy is introduced, aiming to facilitate the algorithm’s escape from local optima. Finally, a best–worst perturbation strategy is developed. The approach not only maintains diversity throughout the optimization process but also enhances the algorithm’s exploitation capabilities. To evaluate the performance of the proposed IMSCSO, we conducted experiments in the CEC 2017 test suite and compared IMSCSO with seven other algorithms. The results show that the IMSCSO proposed in this paper has better optimization performance.

## 1. Introduction

In the current epoch marked by swift technological advancements, we are presented with challenges and opportunities that are truly unmatched in history. The information explosion, coupled with the ascendancy of big data technology, has catapulted optimization problems to the forefront of scientific research and engineering applications. The quest for optimization reaches beyond merely identifying superior solutions to augment system performance; it fundamentally grapples with the challenge of optimizing objective functions to their fullest potential within the confines of scarce resources and stringent constraints. This endeavor necessitates intricate decision-making processes that demand a thorough examination of various dimensions, including but not limited to decision variables, the objective function itself, and the constraints that govern the problem space. Although traditional deterministic algorithms are highly effective in dealing with linear, continuous, differentiable, and convex optimization problems, their limitations are gradually exposed when dealing with complex, nonlinear, and multi-constraint optimization problems in the real world [1]. For example, although Newton’s method uses the Hessian matrix to quickly obtain information about the problem and quickly solve the optimization problem, it requires that the objective function has continuous first- and second-order partial derivatives and the Hessian matrix must be positive definite. In many cases, traditional gradient-based optimization methods terminate the search when the gradient approaches zero, which can happen in both global and local optimal cases, making it difficult to determine the optimal solution. As a result, these methods have limitations in the derivation of the search space and are prone to fall into local optimality with much lower efficiency.

In such a background, metaheuristic algorithms, with their unique stochasticity and global search capability, provide innovative ideas and methods for solving complex optimization problems. Such algorithms do not depend on the specific form of the problem but rather guide the search process by simulating phenomena in nature, behaviors of organisms, physical principles, and social laws, etc., thus demonstrating excellent adaptability and efficiency in numerous application fields. With the continuous development of metaheuristic algorithms, these algorithms play a crucial role in a variety of fields, such as path planning [2,3], image segmentation [4,5], feature selection [6,7], neural network hyper-parameter optimization [8,9], task allocation [10,11], supply chain management [12,13], waste collection [14], wireless sensor optimization problems [15,16], and antenna array synthesis issues [17,18]. And, they show great potential in promoting the development of engineering technology, improving productivity, and solving multi-objective optimization problems [19,20]. Their flexibility and adaptability enable the provision of solutions for different types of problems, ensuring that they play a vital role in practical applications.

Metaheuristic algorithms are based on modeling natural phenomena, animal behavior, physical concepts, and human and other evolutionary processes. They usually fall into four main categories: evolution-based algorithms, physics-based algorithms, swarm-based algorithms, and human-based algorithms.

Evolution-based algorithms are a class of metaheuristic algorithms based on the principles of natural evolution, such as genetic algorithms (GA) [21], based on Darwinian evolution; differential evolutionary (DE) [22], based on the concepts of natural selection and reproduction in Darwinian evolution; genetic programming (GP) [23], inspired by the process of biological evolution; and evolutionary strategies (ES) [24]. Among them, genetic algorithm and differential evolution are widely recognized as the most popular evolutionary algorithms.

Physics-based algorithms are metaheuristic algorithms inspired by various phenomena and principles in physics. For example, simulated annealing (SA) [25] is based on the principle of solid-state annealing in metallurgy. The gravitational search algorithm (GSA) [26] is derived from Newton’s laws of gravity and kinematics. The sine cosine algorithm (SCA) [27] is inspired by the periodic oscillatory properties of the sine and cosine functions and their useful properties in optimization. The multi-verse optimization (MVO) [28] is based on the assumption of the existence of multiple universes in the universe and the possible interactions and evolutionary laws between these universes.

Human-based algorithms are metaheuristic algorithms that solve optimization problems by simulating certain natural human behaviors. For example, teaching-and-learning-based optimization (TLBO) [29] is based on the teacher’s influence on the learner’s output. Social network search (SNS) [30] is inspired by the real-life behaviors of people when they are socializing. The group teaching optimization algorithm (GTOA) [31] draws on the idea that teachers use different teaching methods for different students in the teaching and learning process.

Swarm-based algorithms are metaheuristics inspired by the social behavior of various groups of organisms in nature. For example, particle swarm optimization (PSO) [32] is inspired by the foraging behavior of bird flocks. Ant colony optimization (ACO) [33] is derived from the social foraging behavior of ant colonies. The whale optimization algorithm (WOA) [34] is inspired by the hunting behavior of whales feeding on their prey. Grey wolf optimization (GWO) [35] is based on the social hierarchy and hunting behavior of grey wolf packs. The reptile search algorithm (RSA) [36] is based on the hunting behavior of alligators. The dwarf mongoose optimization (DMO) [37] is developed as an algorithm for optimization by simulating the foraging behavior of the dwarf mongoose. The tuna swarm optimization (TSO) [38] is inspired by two collaborative foraging behaviors of tuna swarms. Maziar et al. proposed a lion optimization algorithm (LOA) [39] based on the special lifestyle and cooperation characteristics of lions. Inspired by the mating pattern of naked mole-rats, Salgotra et al. proposed the naked mole-rat algorithm [40].

The sand cat swarm optimization (SCSO) [41] is a novel swarm-based metaheuristic algorithm. It finds the optimal solution in a suitable sized problem space by studying and imitating the hunting habits of sand cats, finding prey in space as the exploration phase and hunting prey as the exploitation phase. The algorithm is characterized by simplicity, few control parameters, easy implementation, and generality. Currently, SCSO has been widely used in various fields such as feature selection, security factor evaluation, code refactoring, intrusion detection, etc. The no free lunch (NFL) theorem [42] states that no single optimization method can solve all practical problems. Each optimization problem has its own characteristics and constraints, so a metaheuristic algorithm applicable to one class of optimization problems may not be suitable for another class of optimization problems. Therefore, it is important to improve the existing algorithms to fit a wider range of optimization problems and enhance their optimization capabilities.

Amir et al. [43] proposed combining the sand cat swarm optimization algorithm with reinforcement learning techniques to improve its global optimization performance. Wang et al. [44] proposed a chaos-based oppositional adaptive Cauchy sand cat swarm optimization algorithm. The algorithm balances exploration and exploitation through a nonlinear adaptive parameter and introduces a Cauchy variation operator to perturb the search step size. Wu et al. [45] use a triangular walk strategy and a Lévy flight walk strategy to improve the optimization performance of the algorithm. Li et al. [46] used a stochastically varying elite collaborative strategy to enable the algorithm to avoid local optimums and then replaced the SCSO’s linear adaptive parameter with a nonlinear adaptive parameter to enhance the global search capability of the algorithm. Amjad et al. [47] used a memory strategy for secondary selection and filtering of features to improve the optimization performance of the algorithm.

In the SCSO algorithm, each sand cat searches for prey in the search area and then captures the prey. This will imbalance the exploration phase and the exploitation phase of the SCSO algorithm, resulting in the late stage of the algorithm due to the decrease in the efficiency of the sand cat’s movement and the lack of searching ability; it is easy for each sand cat to fall into the local optimal trap and stop searching, preventing the algorithm from finding a better position. In order to solve these problems, an improved multi-strategy sand cat swarm optimization algorithm (IMSCSO) is proposed in this paper. The main contributions of this algorithm are as follows:

1. A roulette fitness–distance balance strategy is proposed. Roulette is used to select individuals to replace the randomly selected individuals in the exploration phase, thus improving the optimization ability of the algorithm.
2. A population perturbation mechanism is proposed. The strategy improves the quality of the sand cat population and is useful for freeing the algorithm from local optima.
3. A best–worst mutation mechanism is proposed. Adjustments are made for the best and worst individual, respectively, to achieve more comprehensive global exploration and local exploitation.

To verify the effectiveness of IMSCSO, experiments were conducted on IEEE CEC2017 test suites whose dimensions were 10, 30, 50, and 100, respectively. It was also compared with the arithmetic optimization algorithm (AOA) [48], salp swarm algorithm (SSA) [49], dung beetle optimizer (DBO) [50], whale optimization algorithm (WOA), aquila optimizer (AO) [51], Harris hawks optimization (HHO) [52], and golden jackal optimization (GJO) [53]. To further evaluate the performance of the algorithms, we statistically analyzed the experimental results using the Wilcoxon rank sum test and Friedman test. Stability analysis and convergence analysis of IMSCSO were also performed to further verify its superior performance.

The subsequent sections of this paper are organized as follows: the second part presents a detailed description of the sand cat swarm optimization algorithm. The third part of this paper shows the detailed description of the proposed IMSCSO. In Section 4, a theoretical analysis of IMSCSO is presented, including the effectiveness analysis of the proposed strategy, numerical analysis, stability analysis, convergence analysis, and statistical tests. Finally, a conclusion and outlook are given in Section 5.

## 2. Sand Cat Swarm Optimization

The sand cat swarm optimization algorithm simulates the hunting behavior of sand cats in a 2 kHz low-frequency noise environment, which consists of two main phases: exploration and hunting. SCSO employs a balancing mechanism to control the different search phases of the algorithm.

### 2.1. Initialization

In the SCSO algorithm, each dune cat is regarded as a search agent of the algorithm, and the population of sand cats formed by all individuals is regarded as the population of the algorithm. First, the whole population of sand cats is initialized. It is similar to other swarm intelligent optimization algorithms, which are randomly generated in the search space, and the population is initialized as shown below:

(1) $$X_{ini}=(ub-lb)\times rand+lb$$

where lb and ub represent the lower and upper bounds of the decision variables, and rand is a random number between 0 and 1.

### 2.2. Search for Prey (Exploration Phase)

The search for prey by sand cats relies on the emission of low-frequency noise, and its sensitivity to low-frequency noise is defined in the SCSO algorithm as $r_{G}$, which is in the range of 0 to 2 Hz. R is the control parameter used to switch between exploration and exploitation. When $\left|R\right|>1$, the sand cat performs the search prey behavior. The computational formula for the exploration phase is expressed as follows:

(2) $$r_{G}=S_{m}-(\frac{S_{m}\times t}{t_{\max}}),$$

(3) $$R=2\times r_{G}\times rand-r_{G},$$

(4) $$Sr=r_{G}\times rand,$$

(5) $$X_{i}^{t+1}=Sr\cdot (X_{r}-rand\times X_{i}^{t})$$

where $S_{m}$ is used to simulate the auditory characteristics of sand cats, with a value of 2 indicating that sand cats can detect low-frequency noise at 2 kHz. t represents the current iteration number, $t_{\max}$ is the maximum number of iterations, and $X_{r}$ denotes an individual randomly selected from the population.

### 2.3. Hunting Prey (Exploitation Phase)

In the SCSO algorithm, the noise-sensitive range of the sand cat is defined as a circular area in order to clearly describe the sand cat’s predation process. In each iteration, the angle amount is randomly calculated using a roulette wheel selection algorithm, which determines the direction in which the sand cat moves within the circular area. The selection of random angles within the entire circular area ranges from 0 to 360°, resulting in a range of values. This approach randomizes a different direction of movement for each sand cat, which enhances the randomness of the algorithm and avoids local convergence. In the predation phase, the position update formula of the sand cat is expressed as follows:

(6) $$X_{i}^{t+1}=X_{best}-Sr\times \left|rand\times X_{best}-X_{i}^{t}\right|\times \cos(\alpha )$$

where $X_{best}$ denotes the global optimal position.

Below is the pseudo-code for the SCSO (Algorithm 1).
**Algorithm 1.** Sand Cat Swarm OptimizationInitialize the population and algorithm parameters Calculate the fitness of the objective function.While (t ≤ $t_{\max}$)        For each agent                Calculate α obtained by Roulette Wheel Selection (−1 ≤ α ≤ 1).                If (abs(R) ≤ 1)                          Update the position based on Equation (6).              Else                          Update the position based on Equation (5).        Endt = t + 1End

## 3. Improved Multi-Strategy Sand Cat Swarm Optimization

This section details the motivation behind our proposed IMSCSO and three improvement strategies, including the roulette fitness–distance balancing strategy, population perturbation strategy, and best–worst perturbation mechanism. In addition, algorithmic complexity analysis is performed.

### 3.1. Roulette Fitness–Distance Balancing Strategy (RFDB)

SCSO refers to a random agent for position updating during the prey search phase. For SCSO, the key to achieving great optimization results is to strike a balance between exploration and exploitation. On the one hand, the algorithm needs to search extensively for areas with development prospects. On the other hand, the algorithm also needs to perform further deep exploitation in the pre-searched promising areas. The fitness–distance balancing strategy (FDB) [54] is a novel selection strategy that is aimed at discovering one or more candidate solutions that contribute the most to the search process of the algorithm. FDB differs from other selection methods in that the selection process is also executed on the basis of the score of the candidate solution, not only its fitness value. In the score calculation, both features, the fitness function values of the candidate solutions and their distance to the optimal solution, are taken into account. This ensures that the candidate solution with the highest score value is selected to guide the population search more efficiently. On the other hand, this also prevents the selection of a candidate solution that is very close to the optimal solution in the population and avoids falling into a local optimum. The implementation steps of the FDB selection method are as follows.

- Calculate the Euclidean distance between each agent and the optimal solution.
- Normalize the obtained Euclidean distance and fitness.
- Sum the weighted Euclidean distance and fitness according to the following formula.

(7) $$S{\mathrm{core}}_{i}=\omega \times normF_{i}+\left(1-\omega \right)\times normD_{i}$$

where ω is a constant taking the value 0.5. $normF_{i}$ is the normalized fitness and $normD_{i}$ is the normalized distance. In IMSCSO, the individual selected using this strategy in the exploration phase will be used to replace the randomly selected individual, which helps to speed up the convergence. In addition to ensure sufficient exploration capacity, the roulette rule is used for selection instead of using the individual with the first FDB score.

(8) $$X_{RFDB}=Select\{X\}$$

where $X_{RFDB}$ is the agent selected using the roulette strategy.

### 3.2. Population Perturbation Strategy (PPS)

The IMSCSO algorithm moves closer to the optimal individual during the exploitation phase and has a higher probability of moving closer to the optimal individual selected by the FDB strategy during the exploration phase. This will accelerate convergence, but there is also the possibility of falling into a local optimum. In order to improve the quality of the sand cat population, the sand cat individuals are perturbed to help the algorithm jump out of the local optimum. The mathematical formula of this strategy is expressed as follows:

(9) $$X_{i}^{t+1}=\left\{\begin{array}{l} X_{i}^{t}+{(1-\frac{t}{t_{\max}})}^{\frac{2t}{t_{\max}}}\times \left(lb+rand\times (\mathrm{ub}-lb)\right)\times U,rand\leq 0.2\\ X_{i}^{t}+[0.2\times (1-rand)+rand]\times \left(X_{r1}^{t}-X_{r2}^{t}\right),rand>0.2 \end{array}\right.$$

where U is a binary vector including 0 or 1. When a random vector from 0 to 1 is generated and is less than 0.2, the array is changed to 0, and vice versa. $X_{r1}^{t}$ and $X_{r2}^{t}$ are two randomly selected agents in the population.

### 3.3. Best Worst Perturbation Mechanism (BWPM)

In SCSO, the quality of the optimal solution has an important impact on the performance of the algorithm; if the optimal solution falls into local optimality, it will lead to other following individuals to fall into local optimality as well. In order to avoid the algorithm from converging prematurely, the optimal solution needs to be perturbed. The non-exclusive learning search strategy is a novel localized search approach that modifies each dimension of the current solution space along the search space. Unlike other local search strategies, this strategy has the ability to get rid of suboptimal solutions due to the inclusion of stochastic operations. In this paper, we utilize the non-exclusive learning search strategy to perform a further search on the optimal individuals as a way to improve the quality of the optimal solution. The specific formula is expressed as follows:

(10) $$X_{new}(j)=rand\times X_{best}(RS)$$

where $X_{new}(j)$ is the j dimension of the new solution. $X_{best}(RS)$ is a random dimension of the optimal solution. RS is a random number between 1 and dim. This formula is used in the first half of the iteration process to help the optimal individual explore the problem space as it continues.

Another formula is executed in the second half of the iteration, which is used to develop the domain of the optimal solution by perturbing it so that it searches for better locations around it, as shown below:

(11) $$X_{new}(j)=X_{best}(j)-\left(X_{best}(RS)\times rand\right)\times eps-\left(X_{best}(j)-NO\right)$$

where eps is a very small value. NO is a tuning parameter used to adjust the search process, which takes the value of 1 in this paper. In the specific optimization process, each individual is not the worst in all dimensions; if each dimension is adjusted, some better dimensions may be discarded. Therefore, in this paper, we use non-exclusive learning to adjust each dimension of the optimal individual one at a time, so as to retain the better dimensions and improve the convergence speed of the algorithm. Furthermore, SCSO only considers the effect of the optimal individual and ignores the effective information of the worst individual. For the worst individual, it contains some effective information to a certain extent, so it is necessary to adjust the worst individual using the following formula:

(12) $$X_{Worst}=X_{Worst}+randn\times \left(X_{Best}-\left|X_{Worst}\right|\right)-randn\times \left(X_{Mean}-\left|X_{Worst}\right|\right)$$

where $X_{Mean}$ is the weighted average position of the dominant population. Individuals with different qualities have different degrees of influence on the worst individual, so they cannot be simply averaged and need to be weight averaged according to the individual ordering to help the worst individual guide to have more chances to escape from the local optimum. The standard normal distribution of random numbers has a larger variation amplitude compared to the uniform random distribution, which can expand the search space of the individuals, so the above formula can effectively improve the quality of the worst individual.

### 3.4. Implementation of IMSCSO

Step 1. Initialization phase: Initialize the population size Np, the population dimension dim, and the max number of iterations $t_{\max}$. The initialized population is calculated using Equation (1).

Step 2. Roulette fitness–distance balancing strategy: during the exploration phase, FDB scores are calculated using Equation (7) and the roulette strategy is used to select an agent to replace the original random agent.

Step 3. Search for prey: when the parameter |*R*| greater than 1, the sand cat searches its prey using Equations (5) and (8).

Step 4. Hunting prey: when the parameter |*R*| is less than or equal to 1, the sand cat hunts prey using Equation (6).

Step 5. Population perturbation strategy: a perturbation is applied to the population according to Equation (9), and a greedy strategy is utilized to select the offspring.

Step 6. Best–worst variance mechanism: A mutation perturbation is applied to the optimal and worst agents. The specific method is shown in Equations (10)–(12).

Step 7. Update position: The position is updated by comparing the fitness values. If the new agent has better fitness, the new agent replaces the original agent. Conversely, the original agent is retained. If the termination condition is met, the run is stopped. Otherwise, go to Step 2.

The pseudo-code for IMSCSO is given by Algorithm 2.
**Algorithm 2.** Improved Multi-Strategy Sand Cat Swarm OptimizationInitialize the population and algorithm parameters Calculate the fitness of the objective function.While (t ≤ $t_{\max}$)        For each Sand cat                Calculate α obtained by Roulette Wheel Selection (−1 ≤ α ≤ 1).                If (abs(R) ≤ 1)                        Update the position based on Equation (6).                Else                        Calculate and select $X_{RFDB}$ based on Equations (7) and (8).                        Update the position based on Equation (5).                End        End        Update the best position through population perturbation strategy based on Equation (9)        Update the best and worst position based on Equations (10)–(12).t = t + 1End

### 3.5. Complexity Analysis of IMSCSO

The time complexity reflects the processing length needed for an algorithm to resolve a problem when its scale is increasing. As for the SCSO with a population size of *Np*, a problem dimension of D, and a maximum number of iterations of *T*, the time complexity of SCSO can be divided into two main parts: population initialization and individual position update. During the initialization, the time complexity for the fitness calculation is $O\left(Np\times D\right)$. The individual position update involves updating the positions of each individual over T iterations, so the time complexity is $O\left(T\times Np\times D\right)$. Therefore, the total time complexity of SCSO is $O\left(Np\times D+T\times Np\times D\right)$. Removing lower-order terms, the overall time complexity of SCSO can be simplified as $O\left(T\times Np\times D\right)$.

For IMSCSO, the initialization process is $O\left(Np\times D\right)$. The search and prey time complexity is $O\left(T\times Np\times D\right)$, the population perturbation strategy (PPS) time complexity is $O\left(T\times Np\times D\right)$, and the best–worst perturbation mechanism (BWPM) time complexity is $O\left(T\times (1+D)\times D\right)$. Thus, the total complexity of IMSCSO is as below.

$$\begin{array}{l} O\left(IMSCSO\right)=O\left(initialization\;process\right)+O\left(search\;and\;prey\;process\right)+O\left(PPS\right)+O\left(BWPM\right)\\ =O\left(Np\times D\right)+O\left(T\times Np\times D\right)+O\left(T\times Np\times D\right)+O\left(T\times (1+D)\times D\right)\\ =O\left(TD\times \left(2Np+D\right)\right) \end{array}$$

The initialization of populations is short and negligible. Removing the lower-order terms again, the final IMSCSO time complexity is $O\left(TD\times \left(2Np+D\right)\right)$. Although the time complexity of IMSCSO becomes larger, the performance of IMSCSO is significantly improved compared to SCSO, so this issue can be accepted.

## 4. Performance Analysis of EDSCSO in CEC 2017

In this section, we will evaluate the performance of the IMSCSO algorithm proposed in this paper on CEC 2017 test suite. Firstly, the specific details of the benchmark test suite used to test the performance of this algorithm will be presented in a tabular form; secondly, the algorithms and their parameter settings compared with the IMSCSO algorithm are shown. Based on this, the efficacy and soundness of the proposed approach are deliberated upon. This paper analyzes all the experiments based on the MATLAB 2020b platform with a 2.90 GHz Intel Core i7-10700F CPU and 16 GB RAM.

### 4.1. Benchmark Functions

The benchmark test function serves as a crucial tool for evaluating the performance of algorithms, offering a standardized platform to assess and compare various optimization optimizers. In this study, we utilize the CEC2017 test suite to evaluate the performance of the proposed MIRIME algorithm across dimensions of 10, 30, and 50, respectively. With increasing dimensionality, the number of local optimal solutions also increases, enabling the suite to effectively evaluate the algorithm’s global optimization capability. Among the 29 test functions in these three test sets, single-peak, multi-peak, and composite functions are included, through which the performance of the IMSCSO algorithm proposed in this paper can be comprehensively tested. For further details regarding CEC2017, please refer to Table 1.

### 4.2. Parameter Setting of Competitors Algorithm

IMSCSO is compared with eight other swarm intelligence optimization algorithms, including AOA, SSA, DBO, WOA, AO, HHO, GJO, and SCSO. Table 2 presents the parameter settings of these optimizers. The maximum number of iterations and population size are set to 1000 and 30, respectively, and each algorithm is run independently 30 times. Subsequently, the best value (Best), the standard deviation (Std), and average value (Ave) are calculated, reflecting the convergence speed and robustness of the algorithm, respectively.

### 4.3. Effectiveness Analysis of Improvement Strategies

In this section, we will verify the effectiveness of each improvement strategy. In this paper, the following three strategies are proposed: roulette fitness–distance balancing strategy, population perturbation mechanism, and best–worst mutation strategy. In order to comprehensively analyze the three strategies, the algorithm combining the roulette adaptive distance balancing strategy is named IMSCSO-1, the algorithm combining the population perturbation mechanism is named IMSCSO-2, and the algorithm combining the best–worst mutation strategy is named IMSCSO-3. The three derived algorithms and SCSO, as well as IMSCSO containing all three strategies, were tested using the 29 functions of CEC2017 in four dimensions: 10, 30, 50, and 100. The parameters were set as follows: the population size Np = 30 and the maximum number of iterations T = 1000. The results are shown in Table 3 and Table 4.

**Figure 1:**
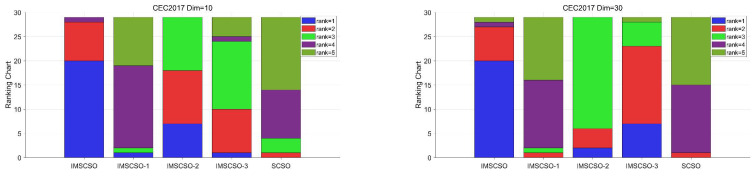

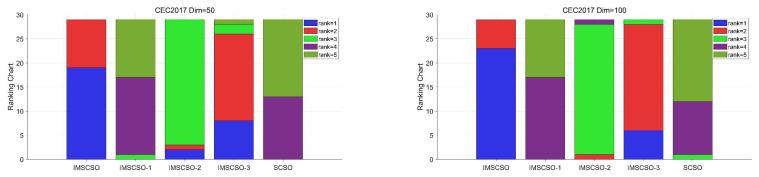
Friedman ranking of each strategy on the CEC2017 test suite.

As shown in Table 3 and Table 4, IMSCSO with three strategies performs the best among all the algorithms involved in the test, while SCSO has the worst performance overall. The rest of the algorithms are ranked from best to worst as IMSCSO-3 > IMSCSO-2 > IMSCSO-1. In order to visualize the performance of different derived algorithms, we illustrate the ranking of different algorithms using stacked bar charts, as shown in Figure 1. We categorize the rankings into six categories: average top rank, average second rank, average third rank, average fourth rank, and average fifth rank. Taking the 10-dimension test as an example, IMSCSO achieved an average top ranking in twenty functions, second in eight functions, and third in one function. As the dimensions increase, IMSCSO still maintains good optimization results. In the case of 30 dimensions, IMSCSO achieved the highest average top rank in twenty functions, second in seven functions and third in one function. In the 50-dimension case, IMSCSO was ranked first in nineteen functions and second in nine functions. In the 100-dimension case, IMSCSO performed even better, ranking first in twenty-three functions and second in six functions. There is no worst ranking in all four dimensions. In general, IMSCSO shows great optimization performance in different dimensions, especially in dealing with high-dimension complex problems, which suggests that our three proposed strategies effectively improve the performance of IMSCSO. For the three derived algorithms and SCSO, IMSCSO-3 is ranked second on most functions. IMSCSO-3 is ranked third in most cases. Although IMSCSO-1 and SCSO have similar average rankings, IMSCSO- outperformed SCSO in solving more than half of the functions.

### 4.4. Comparison with Other Competitive Algorithms

In this section, we utilize the CEC 2017 test suite to evaluate the effectiveness of IMSCSO compared with other competitive algorithms.

#### 4.4.1. Quantitative Analysis

**Figure 2:**
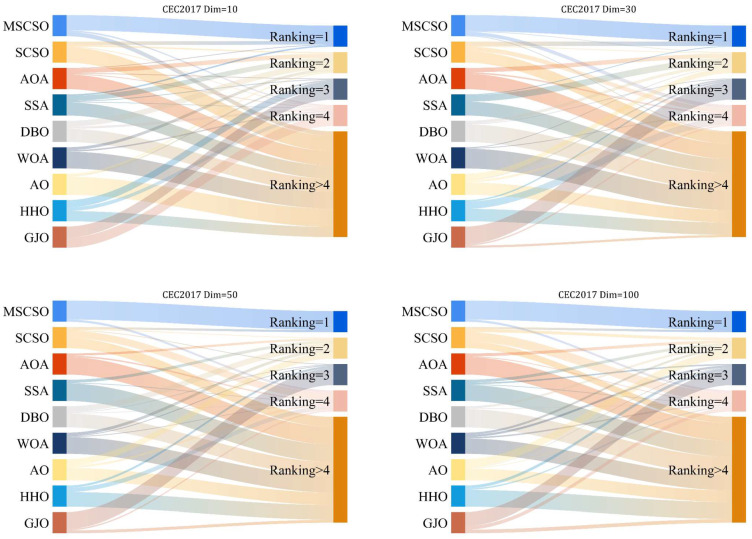
The ranking Sankey of different competitors on CEC2017.

To demonstrate the competitiveness of our proposed IMSCSO, we conducted performance tests using the CEC 2017 suite on four different dimensions: 10, 30, 50, and 100. Table 5, Table 6, Table 7 and Table 8 show the best value (Best), mean (Ave), standard deviation (std), and ranking of IMSCSO and its comparative algorithms for 30 independent runs on different dimensions, respectively. The results show that with the increase in problem dimensions, the performance of other algorithms is greatly affected and they are susceptible to falling into local optimality, while the IMSCSO algorithm shows better stability and robustness. It is worth mentioning that IMSCSO obtains the greatest number of first places in all four dimensions with no underperformance rate. Figure 2 displays the Friedman ranking of IMSCSO and its comparison algorithms in the 10, 30, 50, and 100 dimensions of the CEC2017 test suite with a Sankey diagram. It is worth mentioning that IMSCSO has obtained the most first places in all four dimensions. Specifically, IMSCSO achieved the best performance on the 21 functions tests. SSA, DBO, and HHO were the best performers on two functions each. WOA and GJO gave the best solutions on one function each. When the dimensions were increased to 30, IMSCSO achieved the best ranking on 22 test functions. SA managed to achieve the first place on five functions. SCSO and AO gave the best solution on one function each. The experimental results show that increasing the dimension to 30 does not weaken the performance of IMSCSO. On the contrary, the average ranking of IMSCSO in 30 dimensions also decreases from 1.45 to 1.31. When the dimensionality is further increased to 50 and 100, the average ranking of IMSCSO further decreases compared with other algorithms, which fully verifies the superiority and feasibility of IMSCSO. The ranking of SCSO becomes larger with the increase in dimensionality on the contrary, which again shows that the improvement strategy proposed in this paper can effectively improve the optimization performance of SCSO, especially when dealing with high-dimension problems.

**Figure 3:**
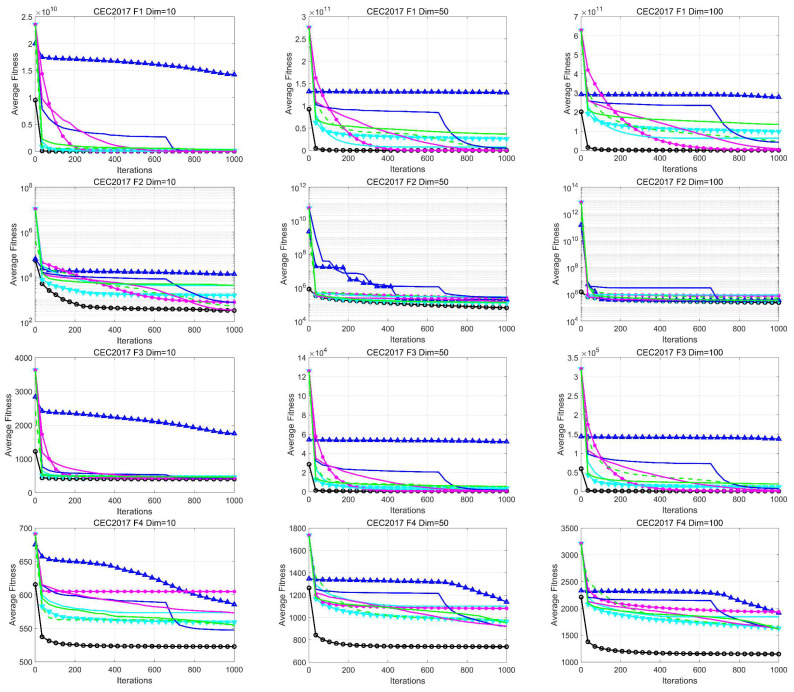

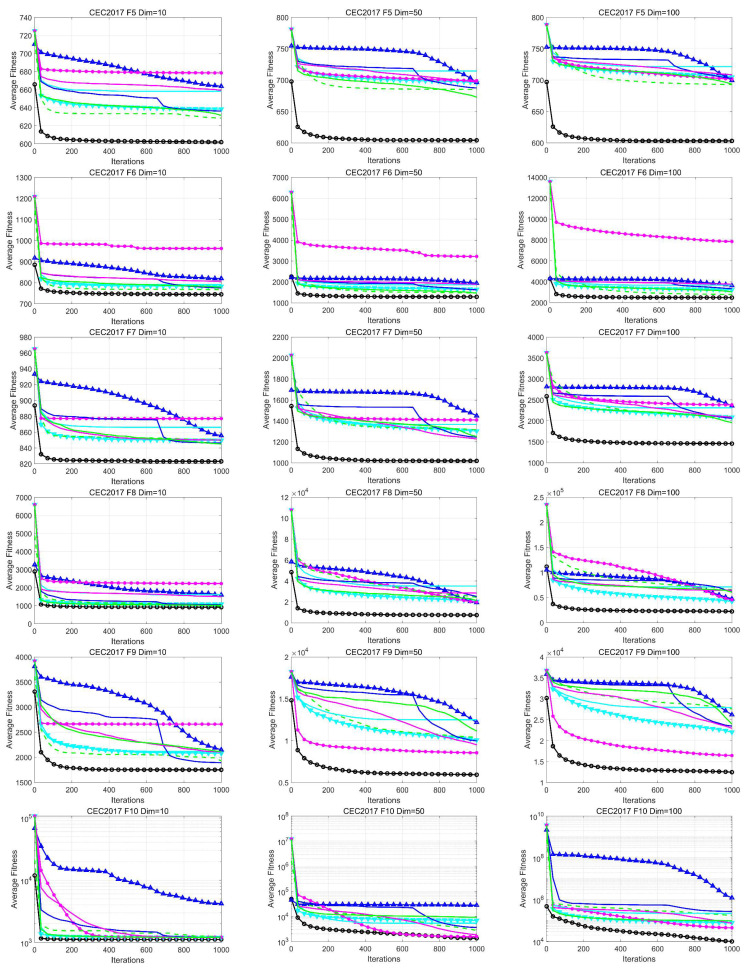

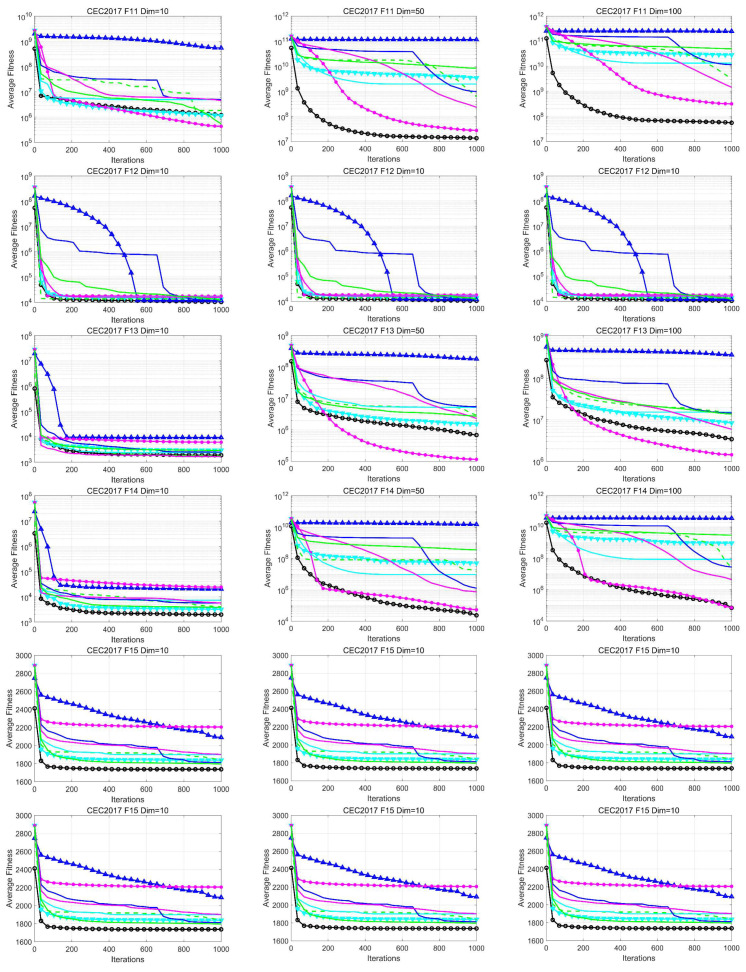

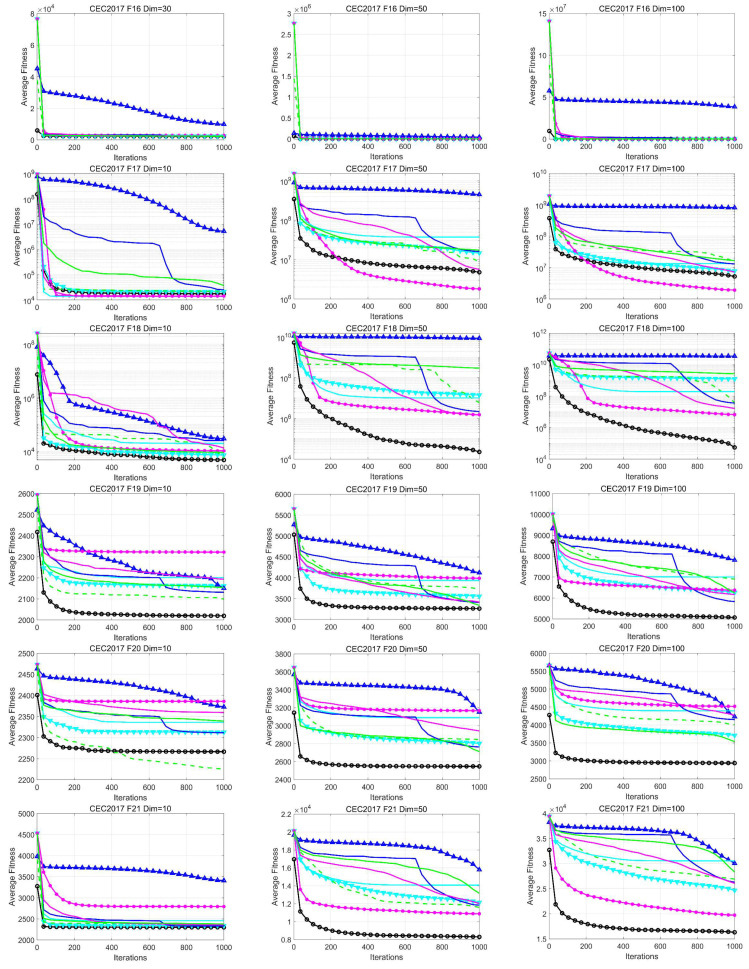

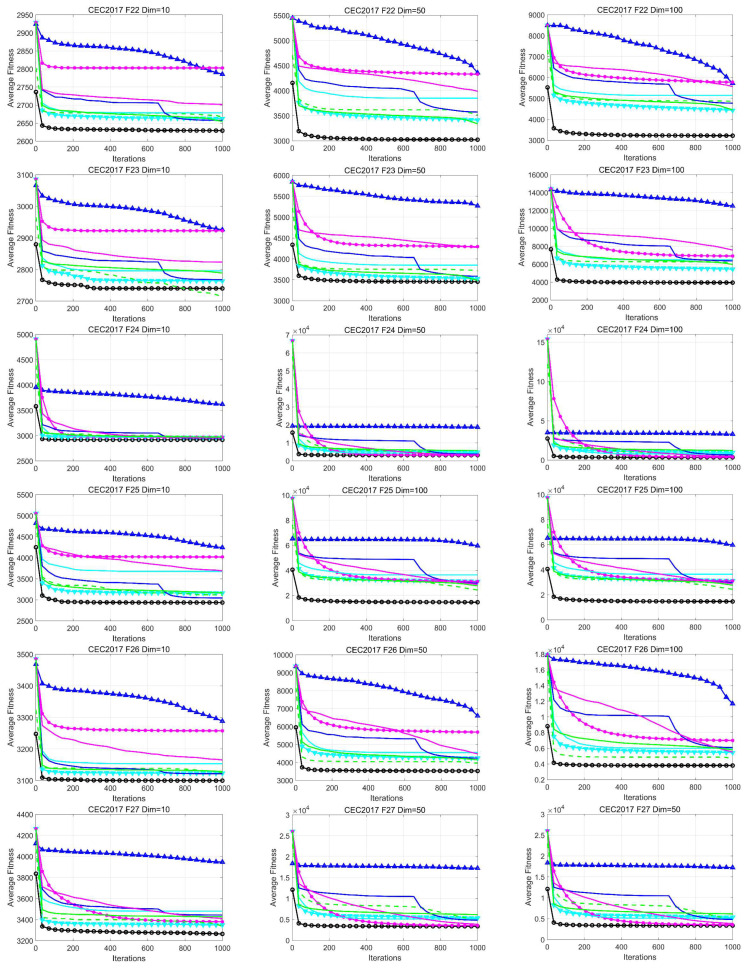

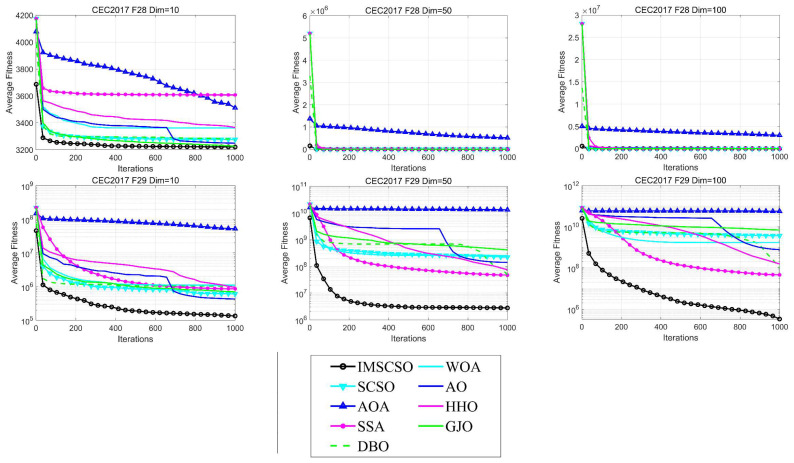
Convergence curve of CEC2017 test function (Dim = 10/50/100).

Figure 3 illustrates the convergence curves of the different algorithms in each dimension. As the number of dimensions increases, the optimization becomes more challenging, and the other algorithms tend to fall into local optima. In contrast, most of the convergence curves of IMSCSO show a continuous downward trend, which indicates that it has strong potential to find the optimal solution and outperforms the comparison algorithms in terms of convergence speed and convergence accuracy. The reasons behind these results are discussed as follows: (1) The roulette fitness–distance balancing strategy enhances the exploitation ability in the exploration phase, guiding the search direction of the IMSCSO population in the exploration phase, while retaining sufficient global exploration ability due to the roulette strategy. (2) The population perturbation mechanism enables the population to be more diverse and improves the convergence accuracy by helping the IMSCSO to get rid of the local optimum. (3) The optimal worst mutation strategy adjusts the optimal individuals to improve the convergence speed accuracy. (4) The best–worst mutation strategy adjusts the optimal individuals to guide the evolutionary direction of the population and enhance the convergence speed. The adjustment of the worst individual helps to enhance the diversity of the population.

**Figure 4:**
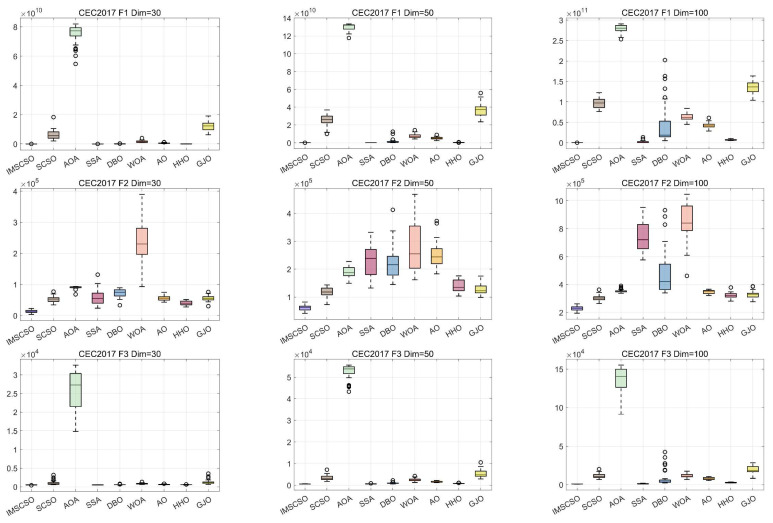

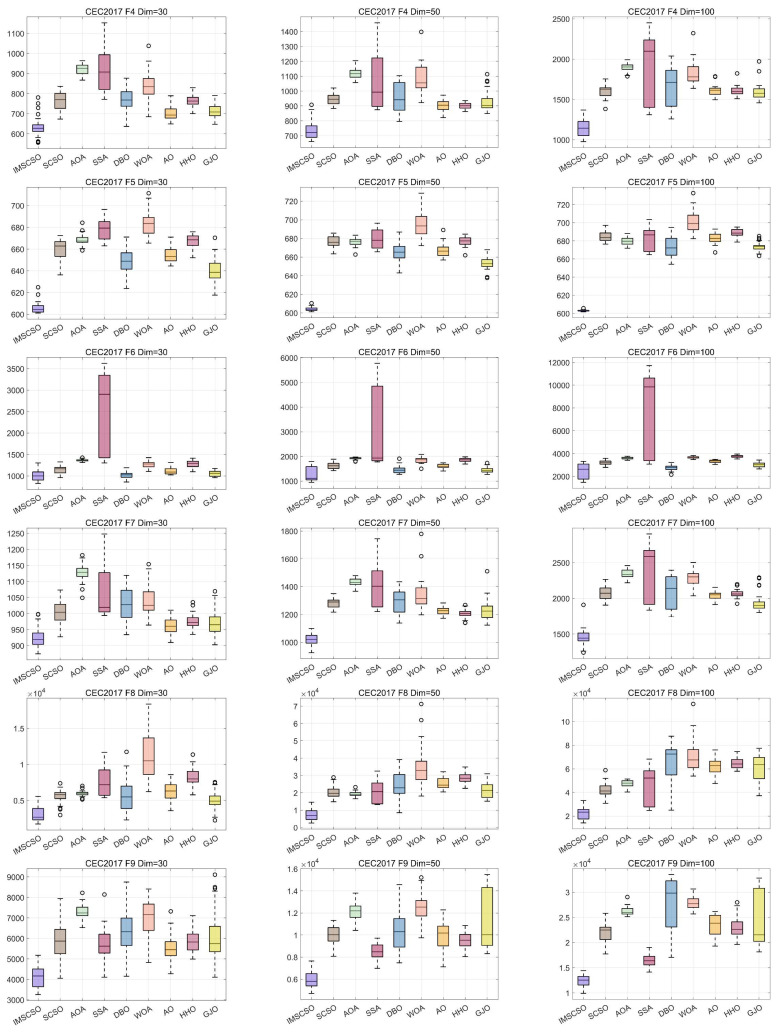

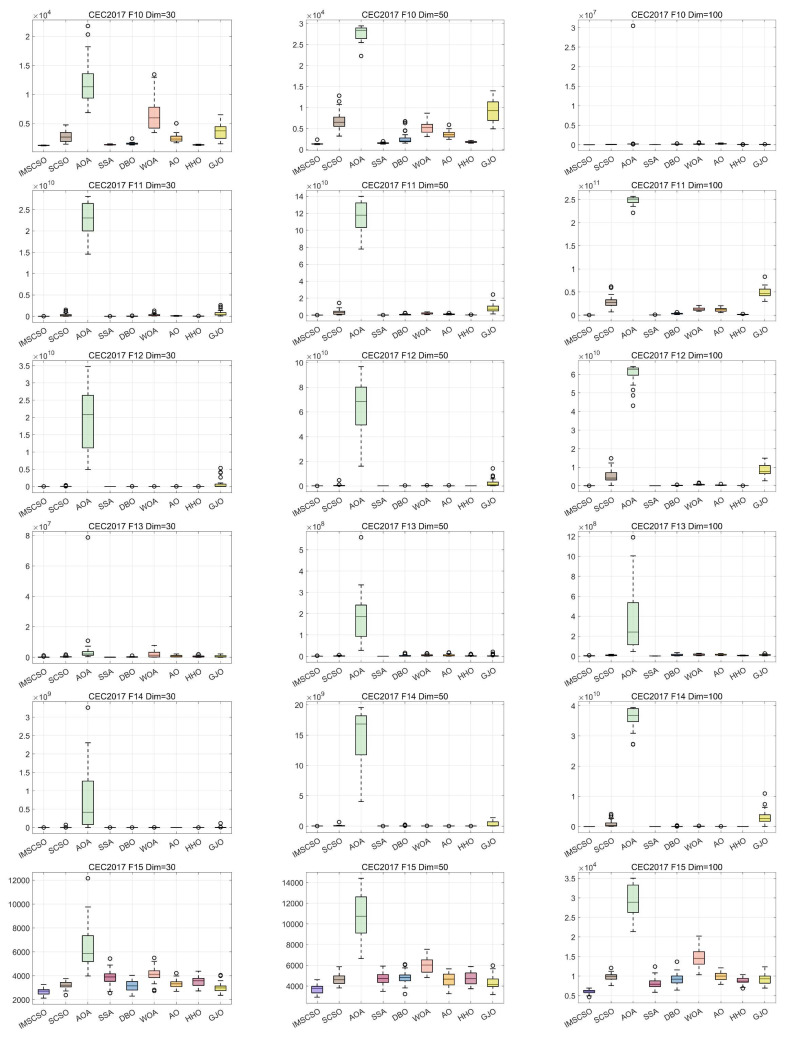

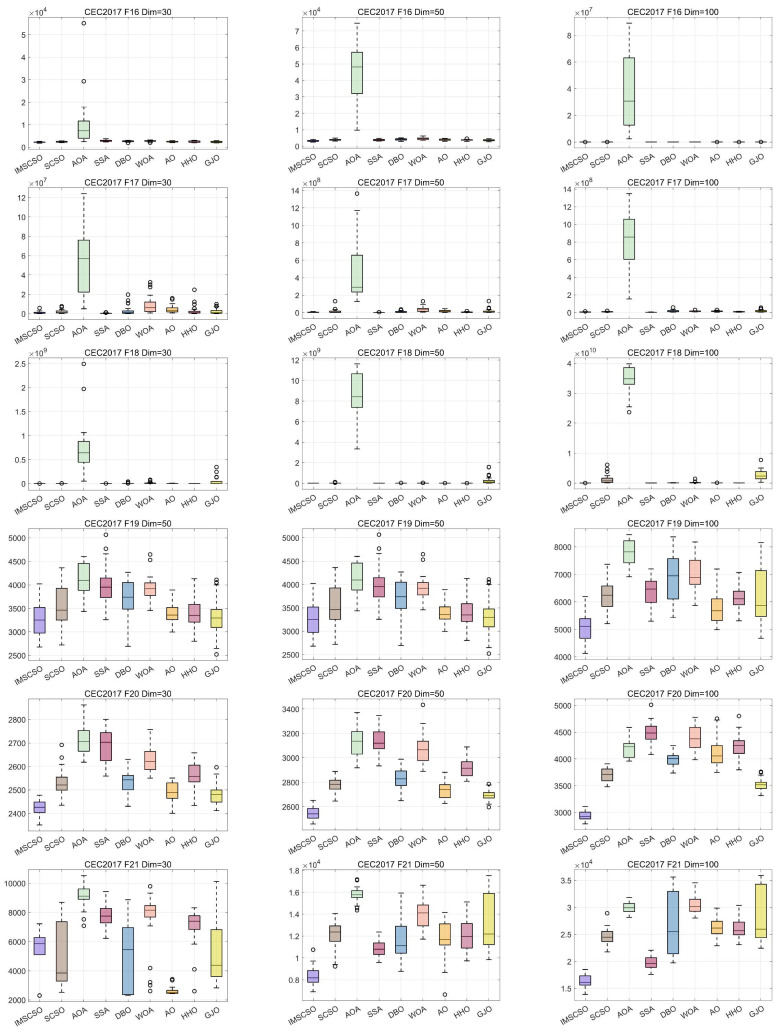

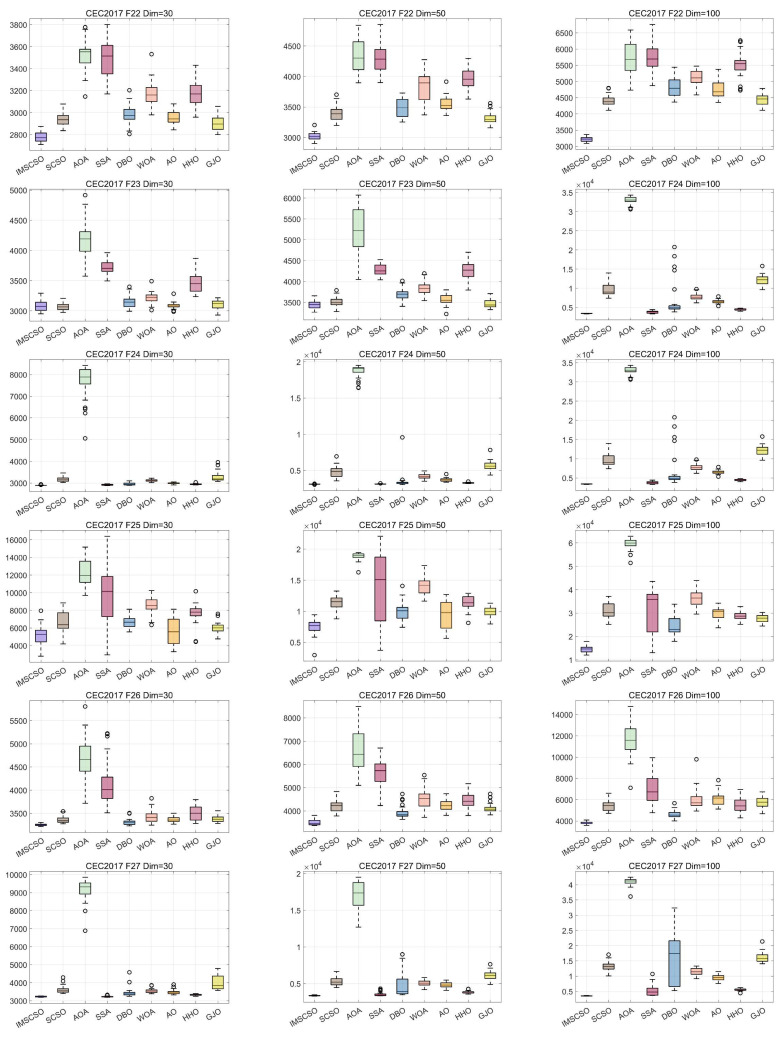

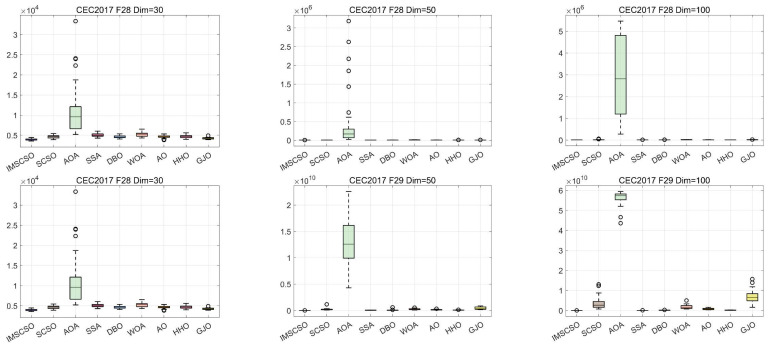
Boxplot analysis for CEC2017 test function (Dim = 30/50/100).

In Figure 4, the performance of the nine algorithms on the three different dimensions of the CEC2017 test set is presented in detail in box-and-line plots, and it is clear that IMSCSO has the best performance. The distribution of solutions for IMSCSO is more centralized and smaller than that of all the other algorithms, which demonstrates the excellent performance of the IMSCSO algorithms in terms of global exploration and local exploitation and verifies their effectiveness and accuracy.

#### 4.4.2. Statistical Analysis

In this section, we will analyze the experimental data using a Wilcoxon test and Friedman test to statistically analyze the performance difference between the IMSCSO algorithm and other algorithms.

To comprehensively highlight the outstanding performance of the proposed algorithm, this section will employ the Wilcoxon rank sum test to verify whether there is a significant difference between the results of each run of IMSCSO and other algorithms at a significance level of p=0.05. When p≤0.05, the hypothesis is rejected, indicating a significant difference between the two algorithms; when p>0.05, the hypothesis is accepted, suggesting no significant difference between the algorithms, i.e., comparable performance. Table 9, Table 10, Table 11 and Table 12, respectively, present the results of IMSCSO and the comparative algorithms on the CEC2017 test suite with dimensions 10, 30, 50, and 100. To highlight the comparative effect, data exceeding 0.05 are displayed in bold, which demonstrate the two algorithms have no differences.

It is clear from the table that the *p*-values for most of the functions in the four dimensions are less than 0.05, which suggests that there is a difference between the optimization results of IMSCSO and the other algorithms. Moreover, the limited occurrence of bolded data, especially in the 50- and 100-dimension test functions, further supports this observation. Furthermore, the number of bold entries decreases as the problem dimensions increase, indicating that the differences between IMSCSO and the other algorithms become more significant. The symbols “+/=/−” are used to indicate whether IMSCSO’s performance is superior, equivalent, or inferior to its competitors. The data in Table 13 show that the performance gap between IMSCSO and other algorithms gradually widens as the dimensionality increases. In conclusion, IMSCSO exhibits the best overall performance, demonstrating the effectiveness of our introduced roulette fitness–distance balancing strategy, population perturbation strategy, and optimal worst variant strategy. These improvements improve the convergence speed and solution accuracy of the algorithm.

We utilized the nonparametric Friedman mean rank test to rank the numerical optimization performance of the IMSCSO algorithm and other optimizers on the CEC2017 test set, and the detailed results are reported in Table 14. IMSCSO consistently ranks first, which highlights that our proposed optimizer significantly outperforms other competing algorithms on the selected test suite.

## 5. Conclusions

Aiming to address the problem that sand cat swarm optimization is susceptible to falling into the local optimum, an improved multi-strategy sand cat swarm optimization is proposed. The algorithm first introduces a roulette fitness–distance balancing strategy in the exploration stage to balance the exploitation and exploration capabilities of the algorithm. In order to solve the problem of low convergence accuracy due to the decrease in population diversity in the later iterations of SCSO, a population perturbation strategy is introduced to improve the optimization ability, which continuously perturbs the population to enhance the population diversity and help the algorithm to jump out of the local optimum effectively. Finally, a best worst mutation strategy is proposed. The mutation of the best individual promotes the exploitation of dominant regions and jumps out of the local optimum. The mutation of the worst individual utilizes the information of the best individual and the dominant population to aid the worst individual in learning effective information during the search process, which further enhances the ability to escape from the local optimum. To test the effectiveness of the proposed method in this paper, we evaluate the performance of IMSCSO using 29 test functions from CEC2017. The experimental results demonstrate its superior performance on test functions of different dimensions. Through two statistical analyses, the Wilcoxon rank sum test and Friedman test, we confirmed the significant advantages of IMSCSO over its competitors. Of course, the method proposed in this paper has some shortcomings, such as high time complexity and no further comparison with other excellent, improved algorithms, such as SHADE variants [55,56], Cuckoo Search variants [57,58], jSO variants [59,60], etc. In future research, we will look at how to reduce the time complexity of the algorithm under the premise of guaranteeing high performance. Moreover, we will try to cooperate with the authors of excellent algorithms to develop high-performance algorithms. Multi-objective versions are also in development. In addition, we plan to apply IMSCSO to practical problems such as path planning, image segmentation, data cluster, parameter optimization, and wireless sensor network coverage.

## Figures and Tables

**Table 1:** Descriptions of CEC-2017 benchmark test functions.

No.	Functions	Search Range	Dim	f_min_
Unimodal functions				
F1	Shifted and Rotated Bent Cigar Function	[−100,100]	10/30/50/100	100
F2	Shifted and Rotated Zakharov Function	[−100,100]	10/30/50/100	300
Simple multimodal functions				
F3	Shifted and Rotated Rosenbrock’s Function	[−100,100]	10/30/50/100	400
F4	Shifted and Rotated Rastrigin’s Function	[−100,100]	10/30/50/100	500
F5	Shifted and Rotated Expanded Scaffer’s F6 Function	[−100,100]	10/30/50/100	600
F6	Shifted and Rotated Lunacek Bi_Rastrigin’s Function	[−100,100]	10/30/50/100	700
F7	Shifted and Rotated Non-Continuous Rastrigin’s Function	[−100,100]	10/30/50/100	800
F8	Shifted and Rotated Levy Function	[−100,100]	10/30/50/100	900
F9	Shifted and Rotated Schwefel’s Function	[−100,100]	10/30/50/100	1000
Hybrid functions				
F10	Hybrid Function 1 (N = 3)	[−100,100]	10/30/50/100	1100
F11	Hybrid Function 2 (N = 3)	[−100,100]	10/30/50/100	1200
F12	Hybrid Function 3 (N = 3)	[−100,100]	10/30/50/100	1300
F13	Hybrid Function 4 (N = 4)	[−100,100]	10/30/50/100	1400
F14	Hybrid Function 5 (N = 4)	[−100,100]	10/30/50/100	1500
F15	Hybrid Function 6 (N = 4)	[−100,100]	10/30/50/100	1600
F16	Hybrid Function 6 (N = 5)	[−100,100]	10/30/50/100	1700
F17	Hybrid Function 6 (N = 5)	[−100,100]	10/30/50/100	1800
F18	Hybrid Function 6 (N = 5)	[−100,100]	10/30/50/100	1900
F19	Hybrid Function 6 (N = 6)	[−100,100]	10/30/50/100	2000
Composition functions				
F20	Composition Function 1 (N = 3)	[−100,100]	10/30/50/100	2100
F21	Composition Function 2 (N = 3)	[−100,100]	10/30/50/100	2200
F22	Composition Function 3 (N = 4)	[−100,100]	10/30/50/100	2300
F23	Composition Function 4 (N = 4)	[−100,100]	10/30/50/100	2400
F24	Composition Function 5 (N = 5)	[−100,100]	10/30/50/100	2500
F25	Composition Function 6 (N = 5)	[−100,100]	10/30/50/100	2600
F26	Composition Function 7 (N = 6)	[−100,100]	10/30/50/100	2700
F27	Composition Function 8 (N = 6)	[−100,100]	10/30/50/100	2800
F28	Composition Function 9 (N = 3)	[−100,100]	10/30/50/100	2900
F29	Composition Function 10 (N = 3)	[−100,100]	10/30/50/100	3000

**Table 2:** Parameter settings of each algorithm.

Algorithms	Name of the Parameter	Value of the Parameter
WOA	a, a2, b	[0,2], [−1,−2], 1
AO	alpha , delta	0.1, 0.1
AOA	Alpha, Mu	5, 0.499
SCSO	S	2
GJO	r	(0, 1)
DBO	p	0.2
SSA	no parameter	No value
HHO	E0, E1, q, r	[−1,1], [0,2], [0,1], [0,1]

**Table 3:** Comparative results of different strategies of IMSCSO (Dim = 10/30).

Function	Index	IMSCSO	IMSCSO-1	IMSCSO-2	IMSCSO-3	SCSO	IMSCSO	IMSCSO-1	IMSCSO-2	IMSCSO-3	SCSO
		Dim = 10					Dim = 30				
F1	Best	1.05 × 10^3^	2.79 × 10^3^	2.55 × 10^3^	1.56 × 10^3^	7.36 × 10^3^	8.77 × 10^4^	1.59 × 10^8^	1.88 × 10^6^	1.69 × 10^5^	2.07 × 10^9^
	Ave	7.31 × 10^3^	1.35 × 10^8^	3.43 × 10^4^	8.93 × 10^3^	1.45 × 10^8^	2.70 × 10^5^	5.51 × 10^9^	2.18 × 10^7^	3.46 × 10^5^	6.22 × 10^9^
	Std	5.63 × 10^3^	3.30 × 10^8^	5.69 × 10^4^	1.06 × 10^4^	3.33 × 10^8^	1.07 × 10^5^	3.40 × 10^9^	1.99 × 10^7^	1.37 × 10^5^	3.38 × 10^9^
	Rank	1	4	3	2	5	1	4	3	2	5
F2	Best	3.00 × 10^2^	3.72 × 10^2^	3.00 × 10^2^	3.01 × 10^2^	3.07 × 10^2^	3.80 × 10^3^	3.14 × 10^4^	1.12 × 10^4^	1.96 × 10^4^	3.42 × 10^4^
	Ave	3.19 × 10^2^	2.05 × 10^3^	3.19 × 10^2^	3.55 × 10^2^	1.49 × 10^3^	1.30 × 10^4^	5.16 × 10^4^	1.97 × 10^4^	3.24 × 10^4^	5.27 × 10^4^
	Std	2.41 × 10^1^	2.32 × 10^3^	1.93 × 10^1^	6.67 × 10^1^	1.56 × 10^3^	4.81 × 10^3^	1.02 × 10^4^	5.54 × 10^3^	6.66 × 10^3^	1.06 × 10^4^
	Rank	1	5	2	3	4	1	4	2	3	5
F3	Best	4.00 × 10^2^	4.05 × 10^2^	4.00 × 10^2^	4.00 × 10^2^	4.00 × 10^2^	4.01 × 10^2^	5.67 × 10^2^	4.85 × 10^2^	4.55 × 10^2^	5.58 × 10^2^
	Ave	4.04 × 10^2^	4.39 × 10^2^	4.15 × 10^2^	4.11 × 10^2^	4.40 × 10^2^	5.04 × 10^2^	1.14 × 10^3^	5.58 × 10^2^	5.09 × 10^2^	1.04 × 10^3^
	Std	2.98 × 10^0^	3.06 × 10^1^	2.53 × 10^1^	1.98 × 10^1^	3.78 × 10^1^	2.95 × 10^1^	7.70 × 10^2^	5.26 × 10^1^	2.64 × 10^1^	6.10 × 10^2^
	Rank	1	4	3	2	5	1	5	3	2	4
F4	Best	5.09 × 10^2^	5.16 × 10^2^	5.12 × 10^2^	5.08 × 10^2^	5.18 × 10^2^	5.56 × 10^2^	6.47 × 10^2^	6.53 × 10^2^	5.54 × 10^2^	6.73 × 10^2^
	Ave	5.23 × 10^2^	5.37 × 10^2^	5.31 × 10^2^	5.26 × 10^2^	5.39 × 10^2^	6.33 × 10^2^	7.53 × 10^2^	7.18 × 10^2^	6.36 × 10^2^	7.62 × 10^2^
	Std	9.17 × 10^0^	1.32 × 10^1^	1.40 × 10^1^	1.23 × 10^1^	1.27 × 10^1^	5.35 × 10^1^	4.22 × 10^1^	3.42 × 10^1^	5.16 × 10^1^	4.64 × 10^1^
	Rank	1	4	3	2	5	1	4	3	2	5
F5	Best	6.00 × 10^2^	6.03 × 10^2^	6.00 × 10^2^	6.00 × 10^2^	6.04 × 10^2^	6.01 × 10^2^	6.44 × 10^2^	6.27 × 10^2^	6.01 × 10^2^	6.36 × 10^2^
	Ave	6.02 × 10^2^	6.14 × 10^2^	6.05 × 10^2^	6.04 × 10^2^	6.18 × 10^2^	6.06 × 10^2^	6.64 × 10^2^	6.47 × 10^2^	6.06 × 10^2^	6.60 × 10^2^
	Std	3.50 × 10^0^	7.38 × 10^0^	5.63 × 10^0^	4.55 × 10^0^	9.40 × 10^0^	5.34 × 10^0^	8.46 × 10^0^	9.71 × 10^0^	4.01 × 10^0^	9.95 × 10^0^
	Rank	1	4	3	2	5	2	5	3	1	4
F6	Best	7.29 × 10^2^	7.24 × 10^2^	7.23 × 10^2^	7.29 × 10^2^	7.34 × 10^2^	8.27 × 10^2^	9.75 × 10^2^	9.62 × 10^2^	8.21 × 10^2^	9.61 × 10^2^
	Ave	7.45 × 10^2^	7.75 × 10^2^	7.49 × 10^2^	7.57 × 10^2^	7.63 × 10^2^	1.02 × 10^3^	1.15 × 10^3^	1.07 × 10^3^	1.07 × 10^3^	1.15 × 10^3^
	Std	1.19 × 10^1^	2.20 × 10^1^	1.43 × 10^1^	2.59 × 10^1^	1.81 × 10^1^	1.43 × 10^2^	9.07 × 10^1^	6.37 × 10^1^	1.61 × 10^2^	8.88 × 10^1^
	Rank	1	5	2	3	4	1	4	2	3	5
F7	Best	8.10 × 10^2^	8.16 × 10^2^	8.11 × 10^2^	8.10 × 10^2^	8.06 × 10^2^	8.75 × 10^2^	9.50 × 10^2^	9.22 × 10^2^	8.69 × 10^2^	9.28 × 10^2^
	Ave	8.23 × 10^2^	8.29 × 10^2^	8.24 × 10^2^	8.26 × 10^2^	8.30 × 10^2^	9.26 × 10^2^	9.95 × 10^2^	9.60 × 10^2^	9.24 × 10^2^	1.00 × 10^3^
	Std	8.25 × 10^0^	9.33 × 10^0^	7.91 × 10^0^	1.02 × 10^1^	7.42 × 10^0^	3.25 × 10^1^	3.05 × 10^1^	1.92 × 10^1^	3.61 × 10^1^	3.19 × 10^1^
	Rank	1	4	2	3	5	2	4	3	1	5
F8	Best	9.00 × 10^2^	9.04 × 10^2^	9.00 × 10^2^	9.00 × 10^2^	9.03 × 10^2^	1.75 × 10^3^	4.70 × 10^3^	2.73 × 10^3^	1.45 × 10^3^	2.98 × 10^3^
	Ave	9.18 × 10^2^	1.08 × 10^3^	9.18 × 10^2^	9.96 × 10^2^	1.07 × 10^3^	3.02 × 10^3^	5.87 × 10^3^	5.07 × 10^3^	3.17 × 10^3^	5.57 × 10^3^
	Std	4.48 × 10^1^	1.41 × 10^2^	3.13 × 10^1^	1.63 × 10^2^	1.56 × 10^2^	9.85 × 10^2^	8.24 × 10^2^	9.70 × 10^2^	1.07 × 10^3^	9.69 × 10^2^
	Rank	2	5	1	3	4	1	5	3	2	4
F9	Best	1.31 × 10^3^	1.03 × 10^3^	1.51 × 10^3^	1.13 × 10^3^	1.57 × 10^3^	3.26 × 10^3^	4.52 × 10^3^	4.29 × 10^3^	3.28 × 10^3^	4.06 × 10^3^
	Ave	1.75 × 10^3^	1.97 × 10^3^	1.89 × 10^3^	1.79 × 10^3^	2.06 × 10^3^	4.13 × 10^3^	5.97 × 10^3^	5.67 × 10^3^	4.14 × 10^3^	5.84 × 10^3^
	Std	2.80 × 10^2^	3.55 × 10^2^	2.85 × 10^2^	3.11 × 10^2^	2.81 × 10^2^	5.35 × 10^2^	7.72 × 10^2^	6.81 × 10^2^	4.37 × 10^2^	7.88 × 10^2^
	Rank	1	4	3	2	5	1	5	3	2	4
F10	Best	1.10 × 10^3^	1.12 × 10^3^	1.10 × 10^3^	1.11 × 10^3^	1.11 × 10^3^	1.14 × 10^3^	1.34 × 10^3^	1.25 × 10^3^	1.16 × 10^3^	1.40 × 10^3^
	Ave	1.12 × 10^3^	1.16 × 10^3^	1.14 × 10^3^	1.12 × 10^3^	1.18 × 10^3^	1.19 × 10^3^	2.51 × 10^3^	1.34 × 10^3^	1.20 × 10^3^	2.76 × 10^3^
	Std	8.35 × 10^0^	4.13 × 10^1^	3.50 × 10^1^	9.00 × 10^0^	5.57 × 10^1^	3.58 × 10^1^	1.01 × 10^3^	6.88 × 10^1^	3.56 × 10^1^	1.07 × 10^3^
	Rank	1	4	3	2	5	1	4	3	2	5
F11	Best	6.61 × 10^4^	1.49 × 10^4^	1.20 × 10^4^	1.22 × 10^4^	4.07 × 10^3^	3.50 × 10^5^	5.32 × 10^6^	1.92 × 10^6^	4.02 × 10^5^	1.18 × 10^7^
	Ave	1.21 × 10^6^	9.40 × 10^5^	1.16 × 10^6^	1.34 × 10^6^	1.15 × 10^6^	3.97 × 10^6^	2.33 × 10^8^	2.25 × 10^7^	3.44 × 10^6^	2.90 × 10^8^
	Std	1.20 × 10^6^	1.04 × 10^6^	2.17 × 10^6^	1.36 × 10^6^	1.72 × 10^6^	4.16 × 10^6^	3.48 × 10^8^	1.78 × 10^7^	2.75 × 10^6^	3.71 × 10^8^
	Rank	4	1	3	5	2	2	4	3	1	5
F12	Best	1.32 × 10^3^	3.16 × 10^3^	1.64 × 10^3^	1.32 × 10^3^	2.90 × 10^3^	3.47 × 10^3^	4.31 × 10^4^	2.08 × 10^4^	2.84 × 10^3^	3.05 × 10^4^
	Ave	1.03 × 10^4^	1.34 × 10^4^	1.17 × 10^4^	9.42 × 10^3^	1.33 × 10^4^	1.57 × 10^5^	4.63 × 10^7^	1.05 × 10^6^	2.39 × 10^5^	3.05 × 10^7^
	Std	7.30 × 10^3^	9.03 × 10^3^	9.03 × 10^3^	8.66 × 10^3^	1.00 × 10^4^	4.04 × 10^5^	8.53 × 10^7^	4.10 × 10^6^	8.16 × 10^5^	6.85 × 10^7^
	Rank	2	5	3	1	4	1	5	3	2	4
F13	Best	1.41 × 10^3^	1.45 × 10^3^	1.44 × 10^3^	1.40 × 10^3^	1.46 × 10^3^	7.34 × 10^3^	4.80 × 10^3^	6.52 × 10^3^	8.05 × 10^3^	7.66 × 10^3^
	Ave	1.88 × 10^3^	2.56 × 10^3^	2.19 × 10^3^	2.53 × 10^3^	2.93 × 10^3^	1.28 × 10^5^	4.31 × 10^5^	1.46 × 10^5^	1.97 × 10^5^	3.34 × 10^5^
	Std	1.16 × 10^3^	1.55 × 10^3^	1.45 × 10^3^	1.70 × 10^3^	1.82 × 10^3^	2.18 × 10^5^	5.83 × 10^5^	2.14 × 10^5^	2.64 × 10^5^	4.14 × 10^5^
	Rank	1	4	2	3	5	1	5	2	3	4
F14	Best	1.50 × 10^3^	1.56 × 10^3^	1.52 × 10^3^	1.52 × 10^3^	1.55 × 10^3^	1.98 × 10^3^	1.40 × 10^4^	9.11 × 10^3^	1.94 × 10^3^	2.80 × 10^4^
	Ave	1.97 × 10^3^	4.26 × 10^3^	1.86 × 10^3^	2.83 × 10^3^	3.17 × 10^3^	9.72 × 10^3^	2.49 × 10^6^	4.36 × 10^4^	8.92 × 10^3^	2.98 × 10^6^
	Std	8.90 × 10^2^	2.62 × 10^3^	5.46 × 10^2^	1.29 × 10^3^	1.40 × 10^3^	7.85 × 10^3^	7.04 × 10^6^	5.35 × 10^4^	8.32 × 10^3^	1.30 × 10^7^
	Rank	2	5	1	3	4	2	4	3	1	5
F15	Best	1.60 × 10^3^	1.64 × 10^3^	1.60 × 10^3^	1.60 × 10^3^	1.60 × 10^3^	2.14 × 10^3^	2.37 × 10^3^	2.35 × 10^3^	2.16 × 10^3^	2.38 × 10^3^
	Ave	1.74 × 10^3^	1.81 × 10^3^	1.73 × 10^3^	1.76 × 10^3^	1.82 × 10^3^	2.69 × 10^3^	3.36 × 10^3^	2.80 × 10^3^	2.76 × 10^3^	3.23 × 10^3^
	Std	9.35 × 10^1^	1.21 × 10^2^	1.14 × 10^2^	1.32 × 10^2^	1.44 × 10^2^	2.80 × 10^2^	4.46 × 10^2^	2.99 × 10^2^	3.23 × 10^2^	3.08 × 10^2^
	Rank	2	4	1	3	5	1	5	3	2	4
F16	Best	1.71 × 10^3^	1.74 × 10^3^	1.72 × 10^3^	1.70 × 10^3^	1.73 × 10^3^	1.79 × 10^3^	1.92 × 10^3^	1.90 × 10^3^	1.79 × 10^3^	2.05 × 10^3^
	Ave	1.74 × 10^3^	1.78 × 10^3^	1.75 × 10^3^	1.75 × 10^3^	1.77 × 10^3^	2.21 × 10^3^	2.36 × 10^3^	2.36 × 10^3^	2.28 × 10^3^	2.42 × 10^3^
	Std	2.41 × 10^1^	2.33 × 10^1^	2.14 × 10^1^	2.89 × 10^1^	1.93 × 10^1^	1.68 × 10^2^	2.51 × 10^2^	2.42 × 10^2^	2.16 × 10^2^	2.23 × 10^2^
	Rank	1	5	3	2	4	1	4	3	2	5
F17	Best	4.37 × 10^3^	2.63 × 10^3^	3.35 × 10^3^	2.20 × 10^3^	2.61 × 10^3^	8.06 × 10^4^	1.12 × 10^5^	1.41 × 10^5^	1.42 × 10^5^	1.47 × 10^5^
	Ave	1.76 × 10^4^	2.11 × 10^4^	2.02 × 10^4^	2.06 × 10^4^	2.14 × 10^4^	1.10 × 10^6^	2.06 × 10^6^	1.27 × 10^6^	1.80 × 10^6^	1.99 × 10^6^
	Std	1.30 × 10^4^	1.40 × 10^4^	1.23 × 10^4^	1.32 × 10^4^	1.61 × 10^4^	1.15 × 10^6^	2.37 × 10^6^	1.20 × 10^6^	2.47 × 10^6^	2.11 × 10^6^
	Rank	1	4	2	3	5	1	5	2	3	4
F18	Best	1.90 × 10^3^	1.92 × 10^3^	1.92 × 10^3^	1.90 × 10^3^	1.93 × 10^3^	2.31 × 10^3^	3.77 × 10^4^	1.09 × 10^4^	2.30 × 10^3^	1.40 × 10^4^
	Ave	4.74 × 10^3^	8.25 × 10^3^	5.12 × 10^3^	8.57 × 10^3^	7.30 × 10^3^	1.41 × 10^4^	1.98 × 10^6^	1.54 × 10^5^	1.85 × 10^4^	1.32 × 10^6^
	Std	4.59 × 10^3^	5.55 × 10^3^	5.03 × 10^3^	6.34 × 10^3^	5.79 × 10^3^	1.45 × 10^4^	1.86 × 10^6^	1.93 × 10^5^	1.76 × 10^4^	1.36 × 10^6^
	Rank	1	4	2	5	3	1	5	3	2	4
F19	Best	2.00 × 10^3^	2.03 × 10^3^	2.01 × 10^3^	2.00 × 10^3^	2.04 × 10^3^	2.22 × 10^3^	2.36 × 10^3^	2.24 × 10^3^	2.19 × 10^3^	2.29 × 10^3^
	Ave	2.02 × 10^3^	2.14 × 10^3^	2.06 × 10^3^	2.07 × 10^3^	2.14 × 10^3^	2.47 × 10^3^	2.71 × 10^3^	2.57 × 10^3^	2.52 × 10^3^	2.70 × 10^3^
	Std	2.54 × 10^1^	6.49 × 10^1^	3.32 × 10^1^	7.34 × 10^1^	6.33 × 10^1^	1.72 × 10^2^	2.04 × 10^2^	1.89 × 10^2^	1.98 × 10^2^	2.06 × 10^2^
	Rank	1	4	2	3	5	1	5	3	2	4
F20	Best	2.20 × 10^3^	2.20 × 10^3^	2.20 × 10^3^	2.20 × 10^3^	2.20 × 10^3^	2.35 × 10^3^	2.40 × 10^3^	2.39 × 10^3^	2.36 × 10^3^	2.43 × 10^3^
	Ave	2.27 × 10^3^	2.29 × 10^3^	2.26 × 10^3^	2.31 × 10^3^	2.29 × 10^3^	2.42 × 10^3^	2.51 × 10^3^	2.47 × 10^3^	2.42 × 10^3^	2.53 × 10^3^
	Std	6.25 × 10^1^	6.04 × 10^1^	6.23 × 10^1^	5.05 × 10^1^	5.85 × 10^1^	3.19 × 10^1^	4.75 × 10^1^	3.63 × 10^1^	3.71 × 10^1^	5.52 × 10^1^
	Rank	2	3	1	5	4	2	4	3	1	5
F21	Best	2.22 × 10^3^	2.25 × 10^3^	2.23 × 10^3^	2.23 × 10^3^	2.26 × 10^3^	2.30 × 10^3^	2.62 × 10^3^	2.31 × 10^3^	2.30 × 10^3^	2.53 × 10^3^
	Ave	2.30 × 10^3^	2.32 × 10^3^	2.30 × 10^3^	2.32 × 10^3^	2.32 × 10^3^	5.23 × 10^3^	4.61 × 10^3^	3.01 × 10^3^	5.06 × 10^3^	5.08 × 10^3^
	Std	1.49 × 10^1^	3.10 × 10^1^	1.41 × 10^1^	1.08 × 10^2^	2.94 × 10^1^	1.71 × 10^3^	2.18 × 10^3^	1.67 × 10^3^	1.79 × 10^3^	2.19 × 10^3^
	Rank	1	5	2	4	3	5	2	1	3	4
F22	Best	2.61 × 10^3^	2.61 × 10^3^	2.61 × 10^3^	2.62 × 10^3^	2.62 × 10^3^	2.71 × 10^3^	2.81 × 10^3^	2.78 × 10^3^	2.74 × 10^3^	2.84 × 10^3^
	Ave	2.63 × 10^3^	2.64 × 10^3^	2.63 × 10^3^	2.64 × 10^3^	2.64 × 10^3^	2.78 × 10^3^	2.92 × 10^3^	2.87 × 10^3^	2.80 × 10^3^	2.93 × 10^3^
	Std	1.06 × 10^1^	1.63 × 10^1^	1.30 × 10^1^	1.12 × 10^1^	1.51 × 10^1^	4.52 × 10^1^	6.45 × 10^1^	5.48 × 10^1^	3.71 × 10^1^	5.61 × 10^1^
	Rank	2	4	1	3	5	1	4	3	2	5
F23	Best	2.50 × 10^3^	2.51 × 10^3^	2.50 × 10^3^	2.50 × 10^3^	2.50 × 10^3^	2.95 × 10^3^	2.97 × 10^3^	2.95 × 10^3^	2.96 × 10^3^	2.98 × 10^3^
	Ave	2.74 × 10^3^	2.74 × 10^3^	2.70 × 10^3^	2.74 × 10^3^	2.74 × 10^3^	3.08 × 10^3^	3.08 × 10^3^	3.03 × 10^3^	3.11 × 10^3^	3.08 × 10^3^
	Std	8.25 × 10^1^	7.38 × 10^1^	1.11 × 10^2^	8.25 × 10^1^	8.28 × 10^1^	8.20 × 10^1^	6.24 × 10^1^	6.15 × 10^1^	8.50 × 10^1^	5.90 × 10^1^
	Rank	2	4	1	3	5	4	3	1	5	2
F24	Best	2.60 × 10^3^	2.90 × 10^3^	2.90 × 10^3^	2.90 × 10^3^	2.91 × 10^3^	2.88 × 10^3^	3.01 × 10^3^	2.88 × 10^3^	2.88 × 10^3^	3.03 × 10^3^
	Ave	2.92 × 10^3^	2.95 × 10^3^	2.92 × 10^3^	2.93 × 10^3^	2.95 × 10^3^	2.90 × 10^3^	3.11 × 10^3^	2.94 × 10^3^	2.90 × 10^3^	3.16 × 10^3^
	Std	6.38 × 10^1^	3.10 × 10^1^	2.40 × 10^1^	2.27 × 10^1^	2.13 × 10^1^	1.35 × 10^1^	8.53 × 10^1^	2.34 × 10^1^	1.90 × 10^1^	1.04 × 10^2^
	Rank	1	5	2	3	4	1	4	3	2	5
F25	Best	2.60 × 10^3^	2.83 × 10^3^	2.80 × 10^3^	2.60 × 10^3^	2.60 × 10^3^	2.81 × 10^3^	4.41 × 10^3^	2.95 × 10^3^	2.81 × 10^3^	4.20 × 10^3^
	Ave	2.94 × 10^3^	3.04 × 10^3^	2.97 × 10^3^	2.99 × 10^3^	3.14 × 10^3^	4.95 × 10^3^	6.66 × 10^3^	4.97 × 10^3^	4.84 × 10^3^	6.59 × 10^3^
	Std	1.42 × 10^2^	1.24 × 10^2^	7.53 × 10^1^	2.84 × 10^2^	2.98 × 10^2^	1.36 × 10^3^	9.87 × 10^2^	1.27 × 10^3^	1.36 × 10^3^	1.23 × 10^3^
	Rank	1	4	2	3	5	2	5	3	1	4
F26	Best	3.09 × 10^3^	3.09 × 10^3^	3.09 × 10^3^	3.09 × 10^3^	3.09 × 10^3^	3.22 × 10^3^	3.27 × 10^3^	3.23 × 10^3^	3.22 × 10^3^	3.27 × 10^3^
	Ave	3.10 × 10^3^	3.11 × 10^3^	3.10 × 10^3^	3.11 × 10^3^	3.10 × 10^3^	3.25 × 10^3^	3.38 × 10^3^	3.32 × 10^3^	3.25 × 10^3^	3.37 × 10^3^
	Std	1.60 × 10^1^	2.19 × 10^1^	1.49 × 10^1^	2.15 × 10^1^	1.84 × 10^1^	2.08 × 10^1^	6.70 × 10^1^	4.84 × 10^1^	2.91 × 10^1^	7.46 × 10^1^
	Rank	2	4	1	5	3	1	5	3	2	4
F27	Best	3.10 × 10^3^	3.17 × 10^3^	3.10 × 10^3^	3.10 × 10^3^	3.10 × 10^3^	3.20 × 10^3^	3.35 × 10^3^	3.26 × 10^3^	3.12 × 10^3^	3.41 × 10^3^
	Ave	3.26 × 10^3^	3.36 × 10^3^	3.33 × 10^3^	3.31 × 10^3^	3.33 × 10^3^	3.23 × 10^3^	3.69 × 10^3^	3.31 × 10^3^	3.22 × 10^3^	3.62 × 10^3^
	Std	1.34 × 10^2^	1.06 × 10^2^	1.12 × 10^2^	1.30 × 10^2^	1.07 × 10^2^	2.31 × 10^1^	2.52 × 10^2^	2.66 × 10^1^	2.70 × 10^1^	2.09 × 10^2^
	Rank	1	5	3	2	4	2	5	3	1	4
F28	Best	3.15 × 10^3^	3.18 × 10^3^	3.14 × 10^3^	3.16 × 10^3^	3.15 × 10^3^	3.58 × 10^3^	4.09 × 10^3^	4.00 × 10^3^	3.66 × 10^3^	3.87 × 10^3^
	Ave	3.22 × 10^3^	3.26 × 10^3^	3.22 × 10^3^	3.24 × 10^3^	3.26 × 10^3^	3.95 × 10^3^	4.57 × 10^3^	4.32 × 10^3^	3.99 × 10^3^	4.59 × 10^3^
	Std	4.73 × 10^1^	6.40 × 10^1^	5.19 × 10^1^	6.65 × 10^1^	7.21 × 10^1^	2.35 × 10^2^	3.65 × 10^2^	2.56 × 10^2^	1.92 × 10^2^	4.00 × 10^2^
	Rank	1	4	2	3	5	1	4	3	2	5
F29	Best	4.72 × 10^3^	6.44 × 10^3^	6.75 × 10^3^	5.71 × 10^3^	4.07 × 10^3^	2.55 × 10^4^	1.31 × 10^6^	1.43 × 10^6^	1.85 × 10^4^	1.76 × 10^6^
	Ave	1.34 × 10^5^	8.41 × 10^5^	3.68 × 10^5^	2.59 × 10^5^	6.06 × 10^5^	1.16 × 10^5^	1.47 × 10^7^	4.92 × 10^6^	1.35 × 10^5^	1.63 × 10^7^
	Std	2.71 × 10^5^	1.29 × 10^6^	5.57 × 10^5^	4.59 × 10^5^	7.84 × 10^5^	7.95 × 10^4^	1.49 × 10^7^	2.41 × 10^6^	1.05 × 10^5^	1.51 × 10^7^
	Rank	1	5	3	2	4	1	4	3	2	5

**Table 4:** Comparative results of different strategies of IMSCSO (Dim = 50/100).

Function	Index	IMSCSO	IMSCSO-1IMSCSO-2		IMSCSO-3	SCSO	IMSCSO	IMSCSO-1IMSCSO-2		IMSCSO-3	SCSO
		Dim = 50					Dim = 100				
F1	Best	5.25 × 10^5^	1.06 × 10^10^	6.08 × 10^7^	9.08 × 10^5^	9.95 × 10^9^	1.12 × 10^7^	6.18 × 10^10^	2.03 × 10^9^	1.10 × 10^7^	7.62 × 10^10^
	Ave	1.76 × 10^6^	2.66 × 10^10^	2.35 × 10^8^	1.75 × 10^6^	2.61 × 10^10^	1.52 × 10^7^	9.47 × 10^10^	4.56 × 10^9^	1.90 × 10^7^	9.75 × 10^10^
	Std	6.96 × 10^5^	6.95 × 10^9^	9.73 × 10^7^	4.69 × 10^5^	6.63 × 10^9^	3.22 × 10^6^	1.50 × 10^10^	1.70 × 10^9^	4.17 × 10^6^	1.35 × 10^10^
	Rank	2	5	3	1	4	1	4	3	2	5
F2	Best	4.12 × 10^4^	7.90 × 10^4^	4.60 × 10^4^	6.13 × 10^4^	7.28 × 10^4^	1.94 × 10^5^	2.62 × 10^5^	2.02 × 10^5^	2.47 × 10^5^	2.65 × 10^5^
	Ave	6.02 × 10^4^	1.12 × 10^5^	7.11 × 10^4^	9.23 × 10^4^	1.17 × 10^5^	2.30 × 10^5^	2.97 × 10^5^	2.56 × 10^5^	2.72 × 10^5^	3.05 × 10^5^
	Std	9.72 × 10^3^	1.98 × 10^4^	1.41 × 10^4^	1.48 × 10^4^	1.86 × 10^4^	1.61 × 10^4^	1.86 × 10^4^	2.73 × 10^4^	1.54 × 10^4^	1.95 × 10^4^
	Rank	1	4	2	3	5	1	4	2	3	5
F3	Best	4.75 × 10^2^	1.60 × 10^3^	5.85 × 10^2^	4.69 × 10^2^	1.73 × 10^3^	6.26 × 10^2^	5.25 × 10^3^	1.48 × 10^3^	6.54 × 10^2^	6.63 × 10^3^
	Ave	5.49 × 10^2^	3.52 × 10^3^	7.48 × 10^2^	5.73 × 10^2^	3.46 × 10^3^	7.65 × 10^2^	1.06 × 10^4^	2.01 × 10^3^	7.77 × 10^2^	1.13 × 10^4^
	Std	4.70 × 10^1^	1.59 × 10^3^	8.49 × 10^1^	5.20 × 10^1^	1.21 × 10^3^	7.65 × 10^1^	3.27 × 10^3^	4.19 × 10^2^	5.81 × 10^1^	3.05 × 10^3^
	Rank	1	5	3	2	4	1	4	3	2	5
F4	Best	6.61 × 10^2^	7.79 × 10^2^	8.26 × 10^2^	6.60 × 10^2^	8.83 × 10^2^	9.77 × 10^2^	1.51 × 10^3^	1.43 × 10^3^	8.90 × 10^2^	1.38 × 10^3^
	Ave	7.38 × 10^2^	9.31 × 10^2^	8.82 × 10^2^	7.60 × 10^2^	9.44 × 10^2^	1.15 × 10^3^	1.60 × 10^3^	1.51 × 10^3^	1.16 × 10^3^	1.61 × 10^3^
	Std	6.43 × 10^1^	4.56 × 10^1^	3.85 × 10^1^	7.58 × 10^1^	4.10 × 10^1^	1.18 × 10^2^	4.84 × 10^1^	5.47 × 10^1^	1.44 × 10^2^	8.45 × 10^1^
	Rank	1	4	3	2	5	1	4	3	2	5
F5	Best	6.01 × 10^2^	6.65 × 10^2^	6.53 × 10^2^	6.02 × 10^2^	6.63 × 10^2^	6.02 × 10^2^	6.73 × 10^2^	6.70 × 10^2^	6.02 × 10^2^	6.77 × 10^2^
	Ave	6.04 × 10^2^	6.74 × 10^2^	6.68 × 10^2^	6.05 × 10^2^	6.76 × 10^2^	6.03 × 10^2^	6.86 × 10^2^	6.80 × 10^2^	6.03 × 10^2^	6.85 × 10^2^
	Std	2.09 × 10^0^	5.18 × 10^0^	8.21 × 10^0^	2.75 × 10^0^	6.18 × 10^0^	8.15 × 10^−1^	5.96 × 10^0^	3.67 × 10^0^	9.31 × 10^−1^	5.97 × 10^0^
	Rank	1	4	3	2	5	1	5	3	2	4
F6	Best	9.47 × 10^2^	1.37 × 10^3^	1.35 × 10^3^	1.01 × 10^3^	1.42 × 10^3^	1.47 × 10^3^	2.79 × 10^3^	2.90 × 10^3^	1.40 × 10^3^	2.79 × 10^3^
	Ave	1.29 × 10^3^	1.68 × 10^3^	1.56 × 10^3^	1.36 × 10^3^	1.63 × 10^3^	2.48 × 10^3^	3.36 × 10^3^	3.25 × 10^3^	2.51 × 10^3^	3.20 × 10^3^
	Std	3.11 × 10^2^	1.37 × 10^2^	1.20 × 10^2^	3.02 × 10^2^	1.26 × 10^2^	6.34 × 10^2^	1.64 × 10^2^	1.58 × 10^2^	6.82 × 10^2^	1.96 × 10^2^
	Rank	1	5	3	2	4	1	5	4	2	3
F7	Best	9.24 × 10^2^	1.15 × 10^3^	1.16 × 10^3^	9.58 × 10^2^	1.22 × 10^3^	1.24 × 10^3^	1.84 × 10^3^	1.81 × 10^3^	1.25 × 10^3^	1.90 × 10^3^
	Ave	1.02 × 10^3^	1.26 × 10^3^	1.21 × 10^3^	1.02 × 10^3^	1.28 × 10^3^	1.46 × 10^3^	2.05 × 10^3^	1.97 × 10^3^	1.44 × 10^3^	2.07 × 10^3^
	Std	4.11 × 10^1^	4.85 × 10^1^	2.89 × 10^1^	3.41 × 10^1^	3.26 × 10^1^	1.20 × 10^2^	8.75 × 10^1^	7.16 × 10^1^	1.35 × 10^2^	9.05 × 10^1^
	Rank	2	4	3	1	5	2	4	3	1	5
F8	Best	2.52 × 10^3^	1.50 × 10^4^	1.17 × 10^4^	3.31 × 10^3^	1.49 × 10^4^	1.42 × 10^4^	2.81 × 10^4^	3.24 × 10^4^	1.48 × 10^4^	3.08 × 10^4^
	Ave	7.35 × 10^3^	1.93 × 10^4^	1.64 × 10^4^	8.25 × 10^3^	2.03 × 10^4^	2.23 × 10^4^	4.36 × 10^4^	4.05 × 10^4^	2.23 × 10^4^	4.22 × 10^4^
	Std	3.41 × 10^3^	3.08 × 10^3^	2.64 × 10^3^	3.30 × 10^3^	3.22 × 10^3^	4.97 × 10^3^	7.88 × 10^3^	5.07 × 10^3^	4.05 × 10^3^	6.03 × 10^3^
	Rank	1	4	3	2	5	1	5	3	2	4
F9	Best	4.70 × 10^3^	7.88 × 10^3^	6.68 × 10^3^	5.13 × 10^3^	8.08 × 10^3^	9.90 × 10^3^	1.84 × 10^4^	1.72 × 10^4^	1.03 × 10^4^	1.77 × 10^4^
	Ave	5.94 × 10^3^	9.77 × 10^3^	9.37 × 10^3^	6.05 × 10^3^	1.01 × 10^4^	1.25 × 10^4^	2.13 × 10^4^	1.99 × 10^4^	1.32 × 10^4^	2.20 × 10^4^
	Std	7.39 × 10^2^	8.45 × 10^2^	1.06 × 10^3^	5.53 × 10^2^	7.55 × 10^2^	1.24 × 10^3^	1.69 × 10^3^	1.63 × 10^3^	1.35 × 10^3^	1.98 × 10^3^
	Rank	1	4	3	2	5	1	4	3	2	5
F10	Best	1.17 × 10^3^	2.85 × 10^3^	1.33 × 10^3^	1.22 × 10^3^	3.20 × 10^3^	3.68 × 10^3^	4.33 × 10^4^	1.47 × 10^4^	8.30 × 10^3^	3.55 × 10^4^
	Ave	1.34 × 10^3^	7.10 × 10^3^	1.76 × 10^3^	1.66 × 10^3^	6.87 × 10^3^	9.83 × 10^3^	7.80 × 10^4^	2.82 × 10^4^	2.29 × 10^4^	7.75 × 10^4^
	Std	2.01 × 10^2^	2.64 × 10^3^	3.73 × 10^2^	6.70 × 10^2^	2.20 × 10^3^	3.45 × 10^3^	1.61 × 10^4^	6.96 × 10^3^	7.95 × 10^3^	1.86 × 10^4^
	Rank	1	5	3	2	4	1	5	3	2	4
F11	Best	3.58 × 10^6^	4.57 × 10^8^	4.12 × 10^7^	1.76 × 10^6^	2.30 × 10^8^	1.39 × 10^7^	8.79 × 10^9^	4.42 × 10^8^	1.51 × 10^7^	6.87 × 10^9^
	Ave	1.35 × 10^7^	4.54 × 10^9^	2.26 × 10^8^	1.69 × 10^7^	3.43 × 10^9^	5.54 × 10^7^	2.70 × 10^10^	1.31 × 10^9^	6.12 × 10^7^	2.82 × 10^10^
	Std	7.44 × 10^6^	3.26 × 10^9^	1.38 × 10^8^	1.18 × 10^7^	2.95 × 10^9^	2.53 × 10^7^	1.05 × 10^10^	5.31 × 10^8^	2.77 × 10^7^	1.22 × 10^10^
	Rank	1	5	3	2	4	1	4	3	2	5
F12	Best	1.25 × 10^4^	1.01 × 10^7^	2.36 × 10^5^	4.77 × 10^4^	2.43 × 10^7^	7.75 × 10^4^	6.19 × 10^8^	3.19 × 10^6^	8.99 × 10^4^	1.26 × 10^8^
	Ave	1.01 × 10^5^	1.12 × 10^9^	5.60 × 10^6^	1.81 × 10^5^	4.29 × 10^8^	1.72 × 10^5^	5.01 × 10^9^	1.59 × 10^7^	2.28 × 10^5^	5.71 × 10^9^
	Std	6.45 × 10^4^	2.23 × 10^9^	7.15 × 10^6^	1.58 × 10^5^	8.25 × 10^8^	5.97 × 10^4^	3.37 × 10^9^	1.27 × 10^7^	1.06 × 10^5^	3.85 × 10^9^
	Rank	1	5	3	2	4	1	4	3	2	5
F13	Best	1.09 × 10^5^	1.58 × 10^5^	8.20 × 10^4^	3.96 × 10^4^	3.80 × 10^4^	1.15 × 10^6^	1.58 × 10^6^	1.43 × 10^6^	9.70 × 10^5^	2.39 × 10^6^
	Ave	6.81 × 10^5^	1.20 × 10^6^	9.29 × 10^5^	8.48 × 10^5^	1.51 × 10^6^	3.41 × 10^6^	8.74 × 10^6^	6.94 × 10^6^	4.29 × 10^6^	8.39 × 10^6^
	Std	5.94 × 10^5^	1.13 × 10^6^	7.88 × 10^5^	7.42 × 10^5^	1.45 × 10^6^	1.81 × 10^6^	4.62 × 10^6^	3.19 × 10^6^	2.33 × 10^6^	4.25 × 10^6^
	Rank	1	4	3	2	5	1	5	3	2	4
F14	Best	2.68 × 10^3^	3.37 × 10^4^	2.28 × 10^4^	6.40 × 10^3^	3.04 × 10^4^	9.37 × 10^3^	6.97 × 10^7^	1.78 × 10^5^	1.96 × 10^4^	2.44 × 10^7^
	Ave	2.33 × 10^4^	1.37 × 10^8^	1.25 × 10^5^	2.48 × 10^4^	5.01 × 10^7^	6.78 × 10^4^	1.20 × 10^9^	2.22 × 10^6^	6.33 × 10^4^	9.32 × 10^8^
	Std	1.66 × 10^4^	4.09 × 10^8^	1.16 × 10^5^	1.26 × 10^4^	1.20 × 10^8^	3.50 × 10^4^	1.32 × 10^9^	2.84 × 10^6^	3.88 × 10^4^	1.15 × 10^9^
	Rank	1	5	3	2	4	2	5	3	1	4
F15	Best	2.92 × 10^3^	3.57 × 10^3^	2.85 × 10^3^	2.57 × 10^3^	3.81 × 10^3^	4.63 × 10^3^	7.05 × 10^3^	7.63 × 10^3^	4.89 × 10^3^	7.56 × 10^3^
	Ave	3.72 × 10^3^	4.68 × 10^3^	4.22 × 10^3^	3.66 × 10^3^	4.62 × 10^3^	6.04 × 10^3^	9.75 × 10^3^	9.45 × 10^3^	5.99 × 10^3^	9.78 × 10^3^
	Std	4.42 × 10^2^	6.29 × 10^2^	5.89 × 10^2^	5.58 × 10^2^	4.80 × 10^2^	5.25 × 10^2^	1.37 × 10^3^	8.46 × 10^2^	6.26 × 10^2^	9.94 × 10^2^
	Rank	2	5	3	1	4	2	4	3	1	5
F16	Best	2.45 × 10^3^	3.13 × 10^3^	2.71 × 10^3^	2.69 × 10^3^	3.09 × 10^3^	3.27 × 10^3^	6.11 × 10^3^	4.37 × 10^3^	4.14 × 10^3^	5.92 × 10^3^
	Ave	3.26 × 10^3^	3.90 × 10^3^	3.51 × 10^3^	3.29 × 10^3^	3.91 × 10^3^	4.79 × 10^3^	1.37 × 10^4^	6.90 × 10^3^	5.15 × 10^3^	1.34 × 10^4^
	Std	4.65 × 10^2^	4.08 × 10^2^	4.51 × 10^2^	2.98 × 10^2^	4.45 × 10^2^	5.40 × 10^2^	1.14 × 10^4^	9.24 × 10^2^	4.86 × 10^2^	1.18 × 10^4^
	Rank	1	4	3	2	5	1	5	3	2	4
F17	Best	7.43 × 10^5^	1.51 × 10^6^	6.26 × 10^5^	6.20 × 10^5^	5.13 × 10^5^	1.74 × 10^6^	3.75 × 10^6^	2.71 × 10^6^	1.50 × 10^6^	2.37 × 10^6^
	Ave	4.75 × 10^6^	9.84 × 10^6^	3.96 × 10^6^	5.10 × 10^6^	1.46 × 10^7^	5.19 × 10^6^	1.15 × 10^7^	6.66 × 10^6^	6.57 × 10^6^	7.73 × 10^6^
	Std	2.65 × 10^6^	1.27 × 10^7^	2.22 × 10^6^	3.42 × 10^6^	2.45 × 10^7^	2.63 × 10^6^	5.38 × 10^6^	3.03 × 10^6^	3.03 × 10^6^	3.96 × 10^6^
	Rank	2	4	1	3	5	1	5	3	2	4
F18	Best	2.71 × 10^3^	6.30 × 10^4^	4.55 × 10^4^	4.60 × 10^3^	1.07 × 10^5^	1.36 × 10^4^	3.62 × 10^7^	1.40 × 10^6^	1.98 × 10^4^	2.99 × 10^7^
	Ave	2.22 × 10^4^	5.99 × 10^7^	1.22 × 10^6^	2.44 × 10^4^	1.40 × 10^7^	5.45 × 10^4^	8.43 × 10^8^	1.60 × 10^7^	5.90 × 10^4^	1.23 × 10^9^
	Std	1.21 × 10^4^	9.89 × 10^7^	1.28 × 10^6^	1.43 × 10^4^	2.87 × 10^7^	3.44 × 10^4^	9.36 × 10^8^	8.58 × 10^6^	4.08 × 10^4^	1.45 × 10^9^
	Rank	1	5	3	2	4	1	4	3	2	5
F19	Best	2.68 × 10^3^	3.03 × 10^3^	2.58 × 10^3^	2.69 × 10^3^	2.72 × 10^3^	4.12 × 10^3^	4.78 × 10^3^	4.89 × 10^3^	4.08 × 10^3^	5.21 × 10^3^
	Ave	3.26 × 10^3^	3.57 × 10^3^	3.34 × 10^3^	3.19 × 10^3^	3.54 × 10^3^	5.07 × 10^3^	5.89 × 10^3^	5.78 × 10^3^	5.00 × 10^3^	6.24 × 10^3^
	Std	3.43 × 10^2^	3.37 × 10^2^	4.03 × 10^2^	3.20 × 10^2^	4.52 × 10^2^	5.50 × 10^2^	6.50 × 10^2^	5.59 × 10^2^	4.70 × 10^2^	5.38 × 10^2^
	Rank	2	5	3	1	4	2	4	3	1	5
F20	Best	2.46 × 10^3^	2.60 × 10^3^	2.59 × 10^3^	2.49 × 10^3^	2.65 × 10^3^	2.79 × 10^3^	3.36 × 10^3^	3.34 × 10^3^	2.82 × 10^3^	3.48 × 10^3^
	Ave	2.55 × 10^3^	2.78 × 10^3^	2.72 × 10^3^	2.55 × 10^3^	2.78 × 10^3^	2.94 × 10^3^	3.64 × 10^3^	3.54 × 10^3^	2.98 × 10^3^	3.69 × 10^3^
	Std	4.96 × 10^1^	7.87 × 10^1^	6.19 × 10^1^	4.12 × 10^1^	6.43 × 10^1^	8.31 × 10^1^	1.59 × 10^2^	1.59 × 10^2^	9.03 × 10^1^	1.28 × 10^2^
	Rank	1	5	3	2	4	1	4	3	2	5
F21	Best	6.91 × 10^3^	9.74 × 10^3^	2.45 × 10^3^	6.60 × 10^3^	9.23 × 10^3^	1.39 × 10^4^	2.14 × 10^4^	2.09 × 10^4^	1.34 × 10^4^	2.18 × 10^4^
	Ave	8.30 × 10^3^	1.17 × 10^4^	1.10 × 10^4^	8.22 × 10^3^	1.21 × 10^4^	1.63 × 10^4^	2.48 × 10^4^	2.39 × 10^4^	1.59 × 10^4^	2.47 × 10^4^
	Std	8.73 × 10^2^	9.87 × 10^2^	1.94 × 10^3^	7.17 × 10^2^	1.22 × 10^3^	1.26 × 10^3^	1.32 × 10^3^	1.81 × 10^3^	1.20 × 10^3^	1.43 × 10^3^
	Rank	2	4	3	1	5	2	5	3	1	4
F22	Best	2.90 × 10^3^	3.21 × 10^3^	3.06 × 10^3^	2.92 × 10^3^	3.20 × 10^3^	3.09 × 10^3^	4.07 × 10^3^	3.80 × 10^3^	3.10 × 10^3^	4.11 × 10^3^
	Ave	3.02 × 10^3^	3.36 × 10^3^	3.25 × 10^3^	3.03 × 10^3^	3.39 × 10^3^	3.21 × 10^3^	4.40 × 10^3^	4.21 × 10^3^	3.23 × 10^3^	4.41 × 10^3^
	Std	6.31 × 10^1^	9.28 × 10^1^	1.28 × 10^2^	5.75 × 10^1^	1.21 × 10^2^	7.24 × 10^1^	1.85 × 10^2^	2.17 × 10^2^	6.37 × 10^1^	1.82 × 10^2^
	Rank	1	4	3	2	5	1	4	3	2	5
F23	Best	3.26 × 10^3^	3.28 × 10^3^	3.08 × 10^3^	3.24 × 10^3^	3.28 × 10^3^	3.75 × 10^3^	4.93 × 10^3^	4.59 × 10^3^	3.76 × 10^3^	4.92 × 10^3^
	Ave	3.45 × 10^3^	3.49 × 10^3^	3.37 × 10^3^	3.53 × 10^3^	3.51 × 10^3^	3.94 × 10^3^	5.55 × 10^3^	5.04 × 10^3^	3.96 × 10^3^	5.42 × 10^3^
	Std	1.15 × 10^2^	9.79 × 10^1^	1.03 × 10^2^	1.42 × 10^2^	1.08 × 10^2^	1.14 × 10^2^	2.94 × 10^2^	2.81 × 10^2^	1.07 × 10^2^	3.06 × 10^2^
	Rank	2	3	1	5	4	1	5	3	2	4
F24	Best	3.03 × 10^3^	3.83 × 10^3^	3.16 × 10^3^	3.03 × 10^3^	3.56 × 10^3^	3.28 × 10^3^	6.45 × 10^3^	4.09 × 10^3^	3.31 × 10^3^	7.44 × 10^3^
	Ave	3.10 × 10^3^	4.75 × 10^3^	3.26 × 10^3^	3.09 × 10^3^	4.82 × 10^3^	3.40 × 10^3^	9.33 × 10^3^	4.46 × 10^3^	3.40 × 10^3^	9.55 × 10^3^
	Std	2.69 × 10^1^	6.01 × 10^2^	7.81 × 10^1^	2.85 × 10^1^	7.32 × 10^2^	5.27 × 10^1^	1.42 × 10^3^	2.38 × 10^2^	6.50 × 10^1^	1.53 × 10^3^
	Rank	2	4	3	1	5	1	4	3	2	5
F25	Best	2.93 × 10^3^	6.41 × 10^3^	3.92 × 10^3^	2.91 × 10^3^	8.77 × 10^3^	1.20 × 10^4^	2.31 × 10^4^	9.61 × 10^3^	1.09 × 10^4^	2.52 × 10^4^
	Ave	7.45 × 10^3^	1.05 × 10^4^	8.20 × 10^3^	7.34 × 10^3^	1.13 × 10^4^	1.46 × 10^4^	3.21 × 10^4^	2.64 × 10^4^	1.48 × 10^4^	3.12 × 10^4^
	Std	1.28 × 10^3^	1.89 × 10^3^	2.58 × 10^3^	1.53 × 10^3^	1.23 × 10^3^	1.50 × 10^3^	3.16 × 10^3^	5.44 × 10^3^	1.67 × 10^3^	3.03 × 10^3^
	Rank	2	4	3	1	5	1	5	3	2	4
F26	Best	3.37 × 10^3^	3.73 × 10^3^	3.53 × 10^3^	3.35 × 10^3^	3.79 × 10^3^	3.58 × 10^3^	4.66 × 10^3^	4.06 × 10^3^	3.56 × 10^3^	4.72 × 10^3^
	Ave	3.53 × 10^3^	4.14 × 10^3^	3.88 × 10^3^	3.58 × 10^3^	4.22 × 10^3^	3.80 × 10^3^	5.39 × 10^3^	4.57 × 10^3^	3.81 × 10^3^	5.43 × 10^3^
	Std	1.36 × 10^2^	2.26 × 10^2^	2.06 × 10^2^	1.07 × 10^2^	2.53 × 10^2^	1.21 × 10^2^	3.95 × 10^2^	2.83 × 10^2^	1.30 × 10^2^	4.63 × 10^2^
	Rank	1	4	3	2	5	1	4	3	2	5
F27	Best	3.28 × 10^3^	4.15 × 10^3^	3.40 × 10^3^	3.29 × 10^3^	4.47 × 10^3^	3.41 × 10^3^	9.14 × 10^3^	4.12 × 10^3^	3.42 × 10^3^	1.01 × 10^4^
	Ave	3.35 × 10^3^	5.32 × 10^3^	3.60 × 10^3^	3.36 × 10^3^	5.32 × 10^3^	3.52 × 10^3^	1.23 × 10^4^	4.69 × 10^3^	3.52 × 10^3^	1.33 × 10^4^
	Std	4.81 × 10^1^	7.12 × 10^2^	1.19 × 10^2^	4.76 × 10^1^	6.05 × 10^2^	5.00 × 10^1^	1.74 × 10^3^	3.93 × 10^2^	4.46 × 10^1^	1.55 × 10^3^
	Rank	1	4	3	2	5	1	4	3	2	5
F28	Best	3.78 × 10^3^	5.27 × 10^3^	4.66 × 10^3^	3.79 × 10^3^	4.99 × 10^3^	6.53 × 10^3^	1.06 × 10^4^	1.02 × 10^4^	6.15 × 10^3^	1.10 × 10^4^
	Ave	4.62 × 10^3^	6.75 × 10^3^	5.92 × 10^3^	4.50 × 10^3^	6.63 × 10^3^	7.45 × 10^3^	1.47 × 10^4^	1.21 × 10^4^	7.31 × 10^3^	1.64 × 10^4^
	Std	3.51 × 10^2^	8.26 × 10^2^	5.74 × 10^2^	3.86 × 10^2^	8.53 × 10^2^	5.77 × 10^2^	4.22 × 10^3^	1.22 × 10^3^	5.58 × 10^2^	1.02 × 10^4^
	Rank	2	5	3	1	4	2	4	3	1	5
F29	Best	8.79 × 10^5^	8.01 × 10^7^	3.11 × 10^7^	1.08 × 10^6^	7.44 × 10^7^	1.38 × 10^5^	4.73 × 10^8^	1.04 × 10^8^	1.04 × 10^5^	7.17 × 10^8^
	Ave	2.77 × 10^6^	2.01 × 10^8^	7.29 × 10^7^	2.82 × 10^6^	2.20 × 10^8^	3.30 × 10^5^	3.81 × 10^9^	2.40 × 10^8^	3.93 × 10^5^	3.62 × 10^9^
	Std	1.77 × 10^6^	1.15 × 10^8^	3.32 × 10^7^	1.75 × 10^6^	1.92 × 10^8^	1.58 × 10^5^	2.40 × 10^9^	1.21 × 10^8^	1.63 × 10^5^	3.05 × 10^9^
	Rank	1	4	3	2	5	1	5	3	2	4

**Table 5:** Comparative results of different algorithms (Dim = 10).

Function	Index	MISCSO	SCSO	AOA	SSA	DBO	WOA	AO	HHO	GJO
F1	Best	1.05 × 10^3^	7.36 × 10^3^	5.63 × 10^9^	1.07 × 10^2^	1.02 × 10^2^	1.38 × 10^6^	1.80 × 10^5^	1.73 × 10^5^	1.48 × 10^5^
	Ave	7.31 × 10^3^	1.45 × 10^8^	1.43 × 10^10^	2.02 × 10^3^	7.88 × 10^5^	2.40 × 10^7^	1.02 × 10^6^	4.81 × 10^5^	2.66 × 10^8^
	Std	5.63 × 10^3^	3.33 × 10^8^	4.95 × 10^9^	1.97 × 10^3^	3.30 × 10^6^	6.47 × 10^7^	8.53 × 10^5^	2.45 × 10^5^	3.42 × 10^8^
	Rank	2	7	9	1	4	6	5	3	8
F2	Best	3.00 × 10^2^	3.07 × 10^2^	7.53 × 10^3^	3.00 × 10^2^	3.00 × 10^2^	4.96 × 10^2^	3.33 × 10^2^	3.02 × 10^2^	4.67 × 10^2^
	Ave	3.19 × 10^2^	1.49 × 10^3^	1.33 × 10^4^	7.34 × 10^2^	4.91 × 10^2^	4.12 × 10^3^	7.08 × 10^2^	3.10 × 10^2^	4.27 × 10^3^
	Std	2.41 × 10^1^	1.56 × 10^3^	1.76 × 10^3^	2.36 × 10^3^	1.01 × 10^3^	4.86 × 10^3^	2.32 × 10^2^	2.11 × 10^1^	3.42 × 10^3^
	Rank	2	6	9	5	3	7	4	1	8
F3	Best	4.00 × 10^2^	4.00 × 10^2^	6.90 × 10^2^	4.00 × 10^2^	4.01 × 10^2^	4.03 × 10^2^	4.01 × 10^2^	4.00 × 10^2^	4.07 × 10^2^
	Ave	4.04 × 10^2^	4.40 × 10^2^	1.74 × 10^3^	4.06 × 10^2^	4.18 × 10^2^	4.59 × 10^2^	4.15 × 10^2^	4.33 × 10^2^	4.41 × 10^2^
	Std	2.98 × 10^0^	3.78 × 10^1^	6.80 × 10^2^	1.05 × 10^1^	2.95 × 10^1^	5.66 × 10^1^	1.98 × 10^1^	3.44 × 10^1^	3.32 × 10^1^
	Rank	1	6	9	2	4	8	3	5	7
F4	Best	5.09 × 10^2^	5.18 × 10^2^	5.33 × 10^2^	5.23 × 10^2^	5.03 × 10^2^	5.25 × 10^2^	5.08 × 10^2^	5.24 × 10^2^	5.08 × 10^2^
	Ave	5.23 × 10^2^	5.39 × 10^2^	5.66 × 10^2^	5.85 × 10^2^	5.33 × 10^2^	5.54 × 10^2^	5.27 × 10^2^	5.53 × 10^2^	5.35 × 10^2^
	Std	9.17 × 10^0^	1.27 × 10^1^	1.94 × 10^1^	3.81 × 10^1^	1.24 × 10^1^	2.04 × 10^1^	1.09 × 10^1^	2.14 × 10^1^	1.25 × 10^1^
	Rank	1	5	8	9	3	7	2	6	4
F5	Best	6.00 × 10^2^	6.04 × 10^2^	6.29 × 10^2^	6.37 × 10^2^	6.00 × 10^2^	6.09 × 10^2^	6.04 × 10^2^	6.19 × 10^2^	6.00 × 10^2^
	Ave	6.02 × 10^2^	6.18 × 10^2^	6.44 × 10^2^	6.59 × 10^2^	6.08 × 10^2^	6.38 × 10^2^	6.16 × 10^2^	6.39 × 10^2^	6.12 × 10^2^
	Std	3.50 × 10^0^	9.40 × 10^0^	6.22 × 10^0^	1.19 × 10^1^	7.87 × 10^0^	1.47 × 10^1^	5.59 × 10^0^	1.03 × 10^1^	7.97 × 10^0^
	Rank	1	5	8	9	2	6	4	7	3
F6	Best	7.29 × 10^2^	7.34 × 10^2^	7.77 × 10^2^	8.05 × 10^2^	7.12 × 10^2^	7.38 × 10^2^	7.23 × 10^2^	7.38 × 10^2^	7.29 × 10^2^
	Ave	7.45 × 10^2^	7.63 × 10^2^	8.00 × 10^2^	9.43 × 10^2^	7.48 × 10^2^	7.72 × 10^2^	7.56 × 10^2^	7.88 × 10^2^	7.59 × 10^2^
	Std	1.19 × 10^1^	1.81 × 10^1^	7.56 × 10^0^	1.54 × 10^2^	2.25 × 10^1^	1.99 × 10^1^	1.85 × 10^1^	2.10 × 10^1^	1.60 × 10^1^
	Rank	1	5	8	9	2	6	3	7	4
F7	Best	8.10 × 10^2^	8.06 × 10^2^	8.26 × 10^2^	8.27 × 10^2^	8.15 × 10^2^	8.17 × 10^2^	8.10 × 10^2^	8.13 × 10^2^	8.12 × 10^2^
	Ave	8.23 × 10^2^	8.30 × 10^2^	8.36 × 10^2^	8.57 × 10^2^	8.29 × 10^2^	8.46 × 10^2^	8.26 × 10^2^	8.30 × 10^2^	8.25 × 10^2^
	Std	8.25 × 10^0^	7.42 × 10^0^	5.90 × 10^0^	2.03 × 10^1^	9.31 × 10^0^	1.89 × 10^1^	7.98 × 10^0^	7.79 × 10^0^	7.67 × 10^0^
	Rank	1	5	7	9	4	8	3	6	2
F8	Best	9.00 × 10^2^	9.03 × 10^2^	1.28 × 10^3^	1.44 × 10^3^	9.01 × 10^2^	1.04 × 10^3^	9.24 × 10^2^	9.99 × 10^2^	9.01 × 10^2^
	Ave	9.18 × 10^2^	1.07 × 10^3^	1.58 × 10^3^	2.21 × 10^3^	9.64 × 10^2^	1.59 × 10^3^	1.04 × 10^3^	1.48 × 10^3^	1.01 × 10^3^
	Std	4.48 × 10^1^	1.56 × 10^2^	1.08 × 10^2^	6.40 × 10^2^	9.99 × 10^1^	4.45 × 10^2^	8.54 × 10^1^	2.57 × 10^2^	9.34 × 10^1^
	Rank	1	5	7	9	2	8	4	6	3
F9	Best	1.31 × 10^3^	1.57 × 10^3^	1.81 × 10^3^	1.91 × 10^3^	1.25 × 10^3^	1.27 × 10^3^	1.26 × 10^3^	1.58 × 10^3^	1.45 × 10^3^
	Ave	1.75 × 10^3^	2.06 × 10^3^	2.13 × 10^3^	2.64 × 10^3^	1.91 × 10^3^	2.10 × 10^3^	1.87 × 10^3^	2.09 × 10^3^	2.09 × 10^3^
	Std	2.80 × 10^2^	2.81 × 10^2^	2.11 × 10^2^	3.56 × 10^2^	2.94 × 10^2^	3.60 × 10^2^	3.03 × 10^2^	2.72 × 10^2^	4.01 × 10^2^
	Rank	1	4	8	9	3	7	2	5	6
F10	Best	1.10 × 10^3^	1.11 × 10^3^	1.19 × 10^3^	1.12 × 10^3^	1.11 × 10^3^	1.12 × 10^3^	1.12 × 10^3^	1.11 × 10^3^	1.12 × 10^3^
	Ave	1.12 × 10^3^	1.18 × 10^3^	4.19 × 10^3^	1.19 × 10^3^	1.20 × 10^3^	1.21 × 10^3^	1.19 × 10^3^	1.18 × 10^3^	1.18 × 10^3^
	Std	8.35 × 10^0^	5.57 × 10^1^	2.02 × 10^3^	4.69 × 10^1^	8.18 × 10^1^	8.09 × 10^1^	5.98 × 10^1^	6.78 × 10^1^	5.34 × 10^1^
	Rank	1	4	9	6	7	8	5	2	3
F11	Best	6.61 × 10^4^	4.07 × 10^3^	8.60 × 10^6^	2.32 × 10^4^	3.39 × 10^3^	8.68 × 10^3^	4.61 × 10^4^	2.84 × 10^4^	1.10 × 10^4^
	Ave	1.21 × 10^6^	1.15 × 10^6^	5.49 × 10^8^	4.34 × 10^5^	1.82 × 10^6^	4.94 × 10^6^	5.05 × 10^6^	4.52 × 10^6^	5.45 × 10^5^
	Std	1.20 × 10^6^	1.72 × 10^6^	5.55 × 10^8^	3.77 × 10^5^	4.81 × 10^6^	5.47 × 10^6^	4.88 × 10^6^	4.80 × 10^6^	6.89 × 10^5^
	Rank	4	3	9	1	5	7	8	6	2
F12	Best	1.32 × 10^3^	2.90 × 10^3^	3.49 × 10^3^	2.47 × 10^3^	1.65 × 10^3^	2.63 × 10^3^	2.57 × 10^3^	2.34 × 10^3^	2.45 × 10^3^
	Ave	1.03 × 10^4^	1.33 × 10^4^	1.17 × 10^4^	1.74 × 10^4^	1.31 × 10^4^	1.64 × 10^4^	1.14 × 10^4^	1.58 × 10^4^	1.18 × 10^4^
	Std	7.30 × 10^3^	1.00 × 10^4^	8.29 × 10^3^	1.48 × 10^4^	1.28 × 10^4^	1.42 × 10^4^	9.59 × 10^3^	1.39 × 10^4^	8.80 × 10^3^
	Rank	1	6	3	9	5	8	2	7	4
F13	Best	1.41 × 10^3^	1.46 × 10^3^	1.47 × 10^3^	1.47 × 10^3^	1.46 × 10^3^	1.48 × 10^3^	1.47 × 10^3^	1.48 × 10^3^	1.47 × 10^3^
	Ave	1.88 × 10^3^	2.93 × 10^3^	9.46 × 10^3^	6.10 × 10^3^	1.70 × 10^3^	2.34 × 10^3^	2.48 × 10^3^	1.63 × 10^3^	2.88 × 10^3^
	Std	1.16 × 10^3^	1.82 × 10^3^	8.97 × 10^3^	5.37 × 10^3^	4.91 × 10^2^	1.33 × 10^3^	1.06 × 10^3^	1.66 × 10^2^	1.68 × 10^3^
	Rank	3	7	9	8	2	4	5	1	6
F14	Best	1.50 × 10^3^	1.55 × 10^3^	1.70 × 10^4^	2.04 × 10^3^	1.65 × 10^3^	1.66 × 10^3^	2.27 × 10^3^	1.78 × 10^3^	1.59 × 10^3^
	Ave	1.97 × 10^3^	3.17 × 10^3^	2.02 × 10^4^	2.41 × 10^4^	4.05 × 10^3^	7.29 × 10^3^	5.59 × 10^3^	5.61 × 10^3^	3.81 × 10^3^
	Std	8.90 × 10^2^	1.40 × 10^3^	2.17 × 10^3^	2.74 × 10^4^	6.03 × 10^3^	5.75 × 10^3^	3.27 × 10^3^	2.32 × 10^3^	1.62 × 10^3^
	Rank	1	2	8	9	4	7	5	6	3
F15	Best	1.60 × 10^3^	1.60 × 10^3^	1.86 × 10^3^	1.83 × 10^3^	1.61 × 10^3^	1.68 × 10^3^	1.62 × 10^3^	1.65 × 10^3^	1.63 × 10^3^
	Ave	1.74 × 10^3^	1.82 × 10^3^	2.07 × 10^3^	2.19 × 10^3^	1.82 × 10^3^	1.88 × 10^3^	1.79 × 10^3^	1.88 × 10^3^	1.78 × 10^3^
	Std	9.35 × 10^1^	1.44 × 10^2^	1.05 × 10^2^	2.25 × 10^2^	1.45 × 10^2^	1.25 × 10^2^	1.26 × 10^2^	1.47 × 10^2^	1.41 × 10^2^
	Rank	1	4	8	9	5	6	3	7	2
F16	Best	1.71 × 10^3^	1.73 × 10^3^	1.75 × 10^3^	1.81 × 10^3^	1.73 × 10^3^	1.74 × 10^3^	1.74 × 10^3^	1.74 × 10^3^	1.74 × 10^3^
	Ave	1.74 × 10^3^	1.77 × 10^3^	1.86 × 10^3^	2.05 × 10^3^	1.78 × 10^3^	1.82 × 10^3^	1.78 × 10^3^	1.81 × 10^3^	1.77 × 10^3^
	Std	2.41 × 10^1^	1.93 × 10^1^	1.02 × 10^2^	2.08 × 10^2^	3.72 × 10^1^	6.05 × 10^1^	2.73 × 10^1^	5.78 × 10^1^	1.93 × 10^1^
	Rank	1	2	8	9	5	7	4	6	3
F17	Best	4.37 × 10^3^	2.61 × 10^3^	3.25 × 10^3^	2.86 × 10^3^	2.17 × 10^3^	2.21 × 10^3^	4.13 × 10^3^	2.31 × 10^3^	8.25 × 10^3^
	Ave	1.76 × 10^4^	2.14 × 10^4^	5.25 × 10^6^	1.41 × 10^4^	2.21 × 10^4^	1.38 × 10^4^	2.55 × 10^4^	1.40 × 10^4^	3.72 × 10^4^
	Std	1.30 × 10^4^	1.61 × 10^4^	2.87 × 10^7^	8.28 × 10^3^	1.64 × 10^4^	8.88 × 10^3^	1.55 × 10^4^	9.33 × 10^3^	1.13 × 10^4^
	Rank	4	5	9	3	6	1	7	2	8
F18	Best	1.90 × 10^3^	1.93 × 10^3^	2.34 × 10^3^	2.20 × 10^3^	1.96 × 10^3^	1.96 × 10^3^	2.01 × 10^3^	2.48 × 10^3^	1.94 × 10^3^
	Ave	4.74 × 10^3^	7.30 × 10^3^	2.98 × 10^4^	1.04 × 10^4^	5.56 × 10^3^	1.96 × 10^4^	2.50 × 10^4^	1.32 × 10^4^	9.05 × 10^3^
	Std	4.59 × 10^3^	5.79 × 10^3^	1.60 × 10^4^	6.50 × 10^3^	7.38 × 10^3^	1.87 × 10^4^	7.12 × 10^4^	8.93 × 10^3^	5.81 × 10^3^
	Rank	1	3	9	5	2	7	8	6	4
F19	Best	2.00 × 10^3^	2.04 × 10^3^	2.05 × 10^3^	2.06 × 10^3^	2.02 × 10^3^	2.05 × 10^3^	2.04 × 10^3^	2.04 × 10^3^	2.05 × 10^3^
	Ave	2.02 × 10^3^	2.14 × 10^3^	2.13 × 10^3^	2.30 × 10^3^	2.08 × 10^3^	2.18 × 10^3^	2.11 × 10^3^	2.17 × 10^3^	2.13 × 10^3^
	Std	2.54 × 10^1^	6.33 × 10^1^	4.68 × 10^1^	1.20 × 10^2^	4.58 × 10^1^	7.98 × 10^1^	5.18 × 10^1^	7.84 × 10^1^	5.54 × 10^1^
	Rank	1	6	4	9	2	8	3	7	5
F20	Best	2.20 × 10^3^	2.20 × 10^3^	2.23 × 10^3^	2.20 × 10^3^	2.20 × 10^3^	2.21 × 10^3^	2.21 × 10^3^	2.20 × 10^3^	2.20 × 10^3^
	Ave	2.27 × 10^3^	2.29 × 10^3^	2.35 × 10^3^	2.37 × 10^3^	2.21 × 10^3^	2.32 × 10^3^	2.29 × 10^3^	2.34 × 10^3^	2.32 × 10^3^
	Std	6.25 × 10^1^	5.85 × 10^1^	3.72 × 10^1^	5.10 × 10^1^	4.92 × 10^0^	5.54 × 10^1^	5.49 × 10^1^	4.86 × 10^1^	3.73 × 10^1^
	Rank	2	4	8	9	1	5	3	7	6
F21	Best	2.22 × 10^3^	2.26 × 10^3^	2.79 × 10^3^	2.30 × 10^3^	2.30 × 10^3^	2.29 × 10^3^	2.30 × 10^3^	2.27 × 10^3^	2.23 × 10^3^
	Ave	2.30 × 10^3^	2.32 × 10^3^	3.39 × 10^3^	2.77 × 10^3^	2.31 × 10^3^	2.44 × 10^3^	2.31 × 10^3^	2.34 × 10^3^	2.36 × 10^3^
	Std	1.49 × 10^1^	2.94 × 10^1^	3.72 × 10^2^	7.41 × 10^2^	1.39 × 10^1^	3.69 × 10^2^	3.81 × 10^0^	1.69 × 10^2^	6.75 × 10^1^
	Rank	1	4	9	8	3	7	2	5	6
F22	Best	2.61 × 10^3^	2.62 × 10^3^	2.66 × 10^3^	2.67 × 10^3^	2.62 × 10^3^	2.63 × 10^3^	2.62 × 10^3^	2.64 × 10^3^	2.61 × 10^3^
	Ave	2.63 × 10^3^	2.64 × 10^3^	2.77 × 10^3^	2.78 × 10^3^	2.65 × 10^3^	2.66 × 10^3^	2.64 × 10^3^	2.68 × 10^3^	2.63 × 10^3^
	Std	1.06 × 10^1^	1.51 × 10^1^	5.18 × 10^1^	7.58 × 10^1^	1.40 × 10^1^	2.57 × 10^1^	1.15 × 10^1^	3.38 × 10^1^	1.11 × 10^1^
	Rank	1	4	8	9	5	6	3	7	2
F23	Best	2.50 × 10^3^	2.50 × 10^3^	2.74 × 10^3^	2.50 × 10^3^	2.50 × 10^3^	2.56 × 10^3^	2.50 × 10^3^	2.50 × 10^3^	2.74 × 10^3^
	Ave	2.74 × 10^3^	2.74 × 10^3^	2.91 × 10^3^	2.90 × 10^3^	2.69 × 10^3^	2.78 × 10^3^	2.75 × 10^3^	2.80 × 10^3^	2.77 × 10^3^
	Std	8.25 × 10^1^	8.28 × 10^1^	9.62 × 10^1^	1.10 × 10^2^	1.15 × 10^2^	4.78 × 10^1^	6.88 × 10^1^	9.29 × 10^1^	1.48 × 10^1^
	Rank	2	3	9	8	1	6	4	7	5
F24	Best	2.60 × 10^3^	2.91 × 10^3^	3.11 × 10^3^	2.90 × 10^3^	2.90 × 10^3^	2.90 × 10^3^	2.90 × 10^3^	2.90 × 10^3^	2.91 × 10^3^
	Ave	2.92 × 10^3^	2.95 × 10^3^	3.60 × 10^3^	2.95 × 10^3^	2.93 × 10^3^	2.95 × 10^3^	2.93 × 10^3^	2.94 × 10^3^	2.95 × 10^3^
	Std	6.38 × 10^1^	2.13 × 10^1^	3.08 × 10^2^	2.08 × 10^1^	2.58 × 10^1^	2.22 × 10^1^	2.28 × 10^1^	3.20 × 10^1^	4.18 × 10^1^
	Rank	1	5	9	6	3	8	2	4	7
F25	Best	2.60 × 10^3^	2.60 × 10^3^	3.32 × 10^3^	2.80 × 10^3^	2.80 × 10^3^	2.88 × 10^3^	2.62 × 10^3^	2.82 × 10^3^	2.90 × 10^3^
	Ave	2.94 × 10^3^	3.14 × 10^3^	4.22 × 10^3^	4.00 × 10^3^	3.09 × 10^3^	3.66 × 10^3^	3.03 × 10^3^	3.67 × 10^3^	3.15 × 10^3^
	Std	1.42 × 10^2^	2.98 × 10^2^	3.71 × 10^2^	5.95 × 10^2^	1.16 × 10^2^	5.75 × 10^2^	1.71 × 10^2^	6.27 × 10^2^	2.67 × 10^2^
	Rank	1	4	9	8	3	6	2	7	5
F26	Best	3.09 × 10^3^	3.09 × 10^3^	3.15 × 10^3^	3.13 × 10^3^	3.09 × 10^3^	3.10 × 10^3^	3.10 × 10^3^	3.10 × 10^3^	3.09 × 10^3^
	Ave	3.10 × 10^3^	3.10 × 10^3^	3.27 × 10^3^	3.24 × 10^3^	3.10 × 10^3^	3.13 × 10^3^	3.10 × 10^3^	3.15 × 10^3^	3.11 × 10^3^
	Std	1.60 × 10^1^	1.84 × 10^1^	6.35 × 10^1^	8.47 × 10^1^	1.55 × 10^1^	4.36 × 10^1^	3.90 × 10^0^	3.93 × 10^1^	1.79 × 10^1^
	Rank	1	3	9	8	4	6	2	7	5
F27	Best	3.10 × 10^3^	3.10 × 10^3^	3.72 × 10^3^	3.10 × 10^3^	3.11 × 10^3^	3.22 × 10^3^	3.26 × 10^3^	3.18 × 10^3^	3.18 × 10^3^
	Ave	3.26 × 10^3^	3.33 × 10^3^	3.93 × 10^3^	3.36 × 10^3^	3.32 × 10^3^	3.46 × 10^3^	3.42 × 10^3^	3.39 × 10^3^	3.41 × 10^3^
	Std	1.34 × 10^2^	1.07 × 10^2^	9.83 × 10^1^	1.06 × 10^2^	1.14 × 10^2^	1.58 × 10^2^	5.10 × 10^1^	1.28 × 10^2^	1.36 × 10^2^
	Rank	1	3	9	4	2	8	7	5	6
F28	Best	3.15 × 10^3^	3.15 × 10^3^	3.28 × 10^3^	3.31 × 10^3^	3.15 × 10^3^	3.19 × 10^3^	3.16 × 10^3^	3.23 × 10^3^	3.16 × 10^3^
	Ave	3.22 × 10^3^	3.26 × 10^3^	3.49 × 10^3^	3.59 × 10^3^	3.25 × 10^3^	3.34 × 10^3^	3.23 × 10^3^	3.35 × 10^3^	3.21 × 10^3^
	Std	4.73 × 10^1^	7.21 × 10^1^	1.39 × 10^2^	1.90 × 10^2^	6.66 × 10^1^	1.02 × 10^2^	3.79 × 10^1^	9.45 × 10^1^	3.88 × 10^1^
	Rank	2	5	8	9	4	6	3	7	1
F29	Best	4.72 × 10^3^	4.07 × 10^3^	2.87 × 10^6^	5.42 × 10^3^	5.22 × 10^3^	7.09 × 10^3^	8.32 × 10^3^	1.33 × 10^4^	8.15 × 10^3^
	Ave	1.34 × 10^5^	6.06 × 10^5^	5.24 × 10^7^	8.58 × 10^5^	9.96 × 10^5^	1.10 × 10^6^	4.28 × 10^5^	9.39 × 10^5^	6.99 × 10^5^
	Std	2.71 × 10^5^	7.84 × 10^5^	6.42 × 10^7^	1.46 × 10^6^	1.34 × 10^6^	1.02 × 10^6^	6.33 × 10^5^	9.97 × 10^5^	1.38 × 10^6^
	Rank	1	3	9	5	7	8	2	6	4

**Table 6:** Comparative results of different algorithms (Dim = 30).

Function	Index	MISCSO	SCSO	AOA	SSA	DBO	WOA	AO	HHO	GJO
F1	Best	8.77 × 10^4^	2.07 × 10^9^	5.47 × 10^10^	2.37 × 10^3^	2.85 × 10^2^	8.38 × 10^8^	2.16 × 10^8^	1.82 × 10^7^	6.29 × 10^9^
	Ave	2.70 × 10^5^	6.22 × 10^9^	7.46 × 10^10^	6.83 × 10^3^	4.97 × 10^7^	1.60 × 10^9^	5.55 × 10^8^	3.07 × 10^7^	1.22 × 10^10^
	Std	1.07 × 10^5^	3.38 × 10^9^	7.06 × 10^9^	3.04 × 10^3^	5.47 × 10^7^	7.04 × 10^8^	2.60 × 10^8^	7.26 × 10^6^	3.25 × 10^9^
	Rank	2	7	9	1	4	6	5	3	8
F2	Best	3.80 × 10^3^	3.42 × 10^4^	6.81 × 10^4^	2.38 × 10^4^	3.31 × 10^4^	9.32 × 10^4^	4.29 × 10^4^	2.75 × 10^4^	3.02 × 10^4^
	Ave	1.30 × 10^4^	5.27 × 10^4^	9.00 × 10^4^	5.83 × 10^4^	7.22 × 10^4^	2.39 × 10^5^	5.57 × 10^4^	3.99 × 10^4^	5.54 × 10^4^
	Std	4.81 × 10^3^	1.06 × 10^4^	4.82 × 10^3^	2.45 × 10^4^	1.31 × 10^4^	7.32 × 10^4^	7.46 × 10^3^	6.99 × 10^3^	9.41 × 10^3^
	Rank	1	3	8	6	7	9	5	2	4
F3	Best	4.01 × 10^2^	5.58 × 10^2^	1.48 × 10^4^	4.67 × 10^2^	5.01 × 10^2^	6.08 × 10^2^	5.50 × 10^2^	4.90 × 10^2^	6.54 × 10^2^
	Ave	5.04 × 10^2^	1.04 × 10^3^	2.62 × 10^4^	5.06 × 10^2^	5.77 × 10^2^	8.30 × 10^2^	6.42 × 10^2^	5.53 × 10^2^	1.22 × 10^3^
	Std	2.95 × 10^1^	6.10 × 10^2^	5.01 × 10^3^	1.79 × 10^1^	6.48 × 10^1^	1.51 × 10^2^	7.81 × 10^1^	4.00 × 10^1^	6.44 × 10^2^
	Rank	1	7	9	2	4	6	5	3	8
F4	Best	5.56 × 10^2^	6.73 × 10^2^	8.67 × 10^2^	7.72 × 10^2^	6.36 × 10^2^	6.85 × 10^2^	6.49 × 10^2^	7.00 × 10^2^	6.47 × 10^2^
	Ave	6.33 × 10^2^	7.62 × 10^2^	9.22 × 10^2^	9.13 × 10^2^	7.64 × 10^2^	8.43 × 10^2^	7.02 × 10^2^	7.62 × 10^2^	7.12 × 10^2^
	Std	5.35 × 10^1^	4.64 × 10^1^	2.64 × 10^1^	1.03 × 10^2^	6.14 × 10^1^	7.42 × 10^1^	3.46 × 10^1^	3.05 × 10^1^	3.88 × 10^1^
	Rank	1	4	9	8	6	7	2	5	3
F5	Best	6.01 × 10^2^	6.36 × 10^2^	6.59 × 10^2^	6.63 × 10^2^	6.24 × 10^2^	6.65 × 10^2^	6.44 × 10^2^	6.52 × 10^2^	6.18 × 10^2^
	Ave	6.06 × 10^2^	6.60 × 10^2^	6.69 × 10^2^	6.78 × 10^2^	6.48 × 10^2^	6.84 × 10^2^	6.54 × 10^2^	6.67 × 10^2^	6.40 × 10^2^
	Std	5.34 × 10^0^	9.95 × 10^0^	5.18 × 10^0^	1.02 × 10^1^	1.18 × 10^1^	1.16 × 10^1^	6.39 × 10^0^	7.01 × 10^0^	1.09 × 10^1^
	Rank	1	5	7	8	3	9	4	6	2
F6	Best	8.27 × 10^2^	9.61 × 10^2^	1.31 × 10^3^	1.31 × 10^3^	8.60 × 10^2^	1.11 × 10^3^	1.02 × 10^3^	1.10 × 10^3^	9.58 × 10^2^
	Ave	1.02 × 10^3^	1.15 × 10^3^	1.36 × 10^3^	2.58 × 10^3^	1.02 × 10^3^	1.27 × 10^3^	1.11 × 10^3^	1.28 × 10^3^	1.06 × 10^3^
	Std	1.43 × 10^2^	8.88 × 10^1^	2.55 × 10^1^	8.97 × 10^2^	8.79 × 10^1^	7.90 × 10^1^	7.93 × 10^1^	8.38 × 10^1^	6.58 × 10^1^
	Rank	1	5	8	9	2	6	4	7	3
F7	Best	8.75 × 10^2^	9.28 × 10^2^	1.05 × 10^3^	9.94 × 10^2^	9.34 × 10^2^	9.64 × 10^2^	9.10 × 10^2^	9.35 × 10^2^	9.03 × 10^2^
	Ave	9.26 × 10^2^	1.00 × 10^3^	1.13 × 10^3^	1.06 × 10^3^	1.03 × 10^3^	1.04 × 10^3^	9.60 × 10^2^	9.76 × 10^2^	9.72 × 10^2^
	Std	3.25 × 10^1^	3.19 × 10^1^	2.86 × 10^1^	8.12 × 10^1^	5.03 × 10^1^	4.70 × 10^1^	2.67 × 10^1^	2.25 × 10^1^	4.22 × 10^1^
	Rank	1	5	9	8	6	7	2	4	3
F8	Best	1.75 × 10^3^	2.98 × 10^3^	5.13 × 10^3^	5.38 × 10^3^	2.30 × 10^3^	6.21 × 10^3^	3.58 × 10^3^	5.78 × 10^3^	2.24 × 10^3^
	Ave	3.02 × 10^3^	5.57 × 10^3^	5.99 × 10^3^	7.61 × 10^3^	5.77 × 10^3^	1.13 × 10^4^	6.26 × 10^3^	8.32 × 10^3^	4.98 × 10^3^
	Std	9.85 × 10^2^	9.69 × 10^2^	3.66 × 10^2^	2.05 × 10^3^	2.18 × 10^3^	3.24 × 10^3^	1.41 × 10^3^	1.19 × 10^3^	1.32 × 10^3^
	Rank	1	3	5	7	4	9	6	8	2
F9	Best	3.26 × 10^3^	4.06 × 10^3^	6.53 × 10^3^	4.11 × 10^3^	4.15 × 10^3^	4.83 × 10^3^	4.27 × 10^3^	5.00 × 10^3^	4.11 × 10^3^
	Ave	4.13 × 10^3^	5.84 × 10^3^	7.29 × 10^3^	5.74 × 10^3^	6.32 × 10^3^	7.00 × 10^3^	5.49 × 10^3^	5.87 × 10^3^	6.18 × 10^3^
	Std	5.35 × 10^2^	7.88 × 10^2^	3.48 × 10^2^	7.87 × 10^2^	1.01 × 10^3^	9.15 × 10^2^	6.47 × 10^2^	5.57 × 10^2^	1.28 × 10^3^
	Rank	1	4	9	3	7	8	2	5	6
F10	Best	1.14 × 10^3^	1.40 × 10^3^	6.87 × 10^3^	1.26 × 10^3^	1.28 × 10^3^	3.40 × 10^3^	1.66 × 10^3^	1.20 × 10^3^	1.45 × 10^3^
	Ave	1.19 × 10^3^	2.76 × 10^3^	1.19 × 10^4^	1.34 × 10^3^	1.54 × 10^3^	6.55 × 10^3^	2.45 × 10^3^	1.30 × 10^3^	3.64 × 10^3^
	Std	3.58 × 10^1^	1.07 × 10^3^	3.60 × 10^3^	6.06 × 10^1^	1.93 × 10^2^	2.72 × 10^3^	6.94 × 10^2^	4.94 × 10^1^	1.28 × 10^3^
	Rank	1	6	9	3	4	8	5	2	7
F11	Best	3.50 × 10^5^	1.18 × 10^7^	1.46 × 10^10^	9.77 × 10^5^	3.59 × 10^5^	3.73 × 10^7^	9.75 × 10^6^	4.31 × 10^6^	5.70 × 10^7^
	Ave	3.97 × 10^6^	2.90 × 10^8^	2.28 × 10^10^	3.35 × 10^6^	2.66 × 10^7^	2.90 × 10^8^	8.89 × 10^7^	2.56 × 10^7^	7.08 × 10^8^
	Std	4.16 × 10^6^	3.71 × 10^8^	3.94 × 10^9^	2.15 × 10^6^	3.28 × 10^7^	2.69 × 10^8^	6.16 × 10^7^	1.65 × 10^7^	6.69 × 10^8^
	Rank	2	7	9	1	4	6	5	3	8
F12	Best	3.47 × 10^3^	3.05 × 10^4^	4.93 × 10^9^	3.17 × 10^4^	1.44 × 10^4^	1.35 × 10^5^	3.99 × 10^5^	2.36 × 10^5^	1.67 × 10^5^
	Ave	1.57 × 10^5^	3.05 × 10^7^	1.94 × 10^10^	1.10 × 10^5^	4.47 × 10^6^	1.63 × 10^6^	2.29 × 10^6^	6.60 × 10^5^	7.92 × 10^8^
	Std	4.04 × 10^5^	6.85 × 10^7^	8.38 × 10^9^	5.93 × 10^4^	9.52 × 10^6^	1.39 × 10^6^	3.81 × 10^6^	3.80 × 10^5^	1.43 × 10^9^
	Rank	2	7	9	1	6	4	5	3	8
F13	Best	7.34 × 10^3^	7.66 × 10^3^	4.24 × 10^5^	4.08 × 10^3^	1.82 × 10^4^	5.98 × 10^4^	1.10 × 10^5^	2.90 × 10^4^	3.39 × 10^4^
	Ave	1.28 × 10^5^	3.34 × 10^5^	5.27 × 10^6^	3.45 × 10^4^	1.99 × 10^5^	2.01 × 10^6^	7.83 × 10^5^	5.91 × 10^5^	6.33 × 10^5^
	Std	2.18 × 10^5^	4.14 × 10^5^	1.41 × 10^7^	3.13 × 10^4^	2.14 × 10^5^	2.26 × 10^6^	5.75 × 10^5^	5.07 × 10^5^	5.82 × 10^5^
	Rank	2	4	9	1	3	8	7	5	6
F14	Best	1.98 × 10^3^	2.80 × 10^4^	9.31 × 10^3^	1.40 × 10^4^	5.81 × 10^3^	1.00 × 10^5^	3.16 × 10^4^	3.02 × 10^4^	1.47 × 10^4^
	Ave	9.72 × 10^3^	2.98 × 10^6^	6.95 × 10^8^	6.88 × 10^4^	1.04 × 10^5^	8.11 × 10^5^	1.26 × 10^5^	1.09 × 10^5^	5.64 × 10^6^
	Std	7.85 × 10^3^	1.30 × 10^7^	7.90 × 10^8^	6.38 × 10^4^	1.47 × 10^5^	1.10 × 10^6^	6.40 × 10^4^	5.57 × 10^4^	2.10 × 10^7^
	Rank	1	7	9	2	3	6	5	4	8
F15	Best	2.14 × 10^3^	2.38 × 10^3^	3.99 × 10^3^	2.55 × 10^3^	2.31 × 10^3^	2.74 × 10^3^	2.70 × 10^3^	2.74 × 10^3^	2.37 × 10^3^
	Ave	2.69 × 10^3^	3.23 × 10^3^	6.46 × 10^3^	3.87 × 10^3^	3.17 × 10^3^	4.11 × 10^3^	3.32 × 10^3^	3.56 × 10^3^	3.00 × 10^3^
	Std	2.80 × 10^2^	3.08 × 10^2^	1.86 × 10^3^	6.23 × 10^2^	4.49 × 10^2^	6.38 × 10^2^	3.59 × 10^2^	3.83 × 10^2^	4.04 × 10^2^
	Rank	1	4	9	7	3	8	5	6	2
F16	Best	1.79 × 10^3^	2.05 × 10^3^	2.46 × 10^3^	2.30 × 10^3^	1.91 × 10^3^	2.00 × 10^3^	2.06 × 10^3^	1.95 × 10^3^	1.94 × 10^3^
	Ave	2.21 × 10^3^	2.42 × 10^3^	9.92 × 10^3^	2.85 × 10^3^	2.63 × 10^3^	2.74 × 10^3^	2.47 × 10^3^	2.48 × 10^3^	2.36 × 10^3^
	Std	1.68 × 10^2^	2.23 × 10^2^	1.04 × 10^4^	3.18 × 10^2^	2.81 × 10^2^	2.90 × 10^2^	2.14 × 10^2^	3.29 × 10^2^	2.74 × 10^2^
	Rank	1	3	9	8	6	7	4	5	2
F17	Best	8.06 × 10^4^	1.47 × 10^5^	5.07 × 10^6^	9.94 × 10^4^	8.90 × 10^4^	7.29 × 10^5^	5.69 × 10^5^	8.16 × 10^4^	8.71 × 10^4^
	Ave	1.10 × 10^6^	1.99 × 10^6^	5.27 × 10^7^	3.67 × 10^5^	3.01 × 10^6^	8.91 × 10^6^	4.59 × 10^6^	2.73 × 10^6^	2.27 × 10^6^
	Std	1.15 × 10^6^	2.11 × 10^6^	3.36 × 10^7^	2.92 × 10^5^	4.66 × 10^6^	8.80 × 10^6^	4.11 × 10^6^	4.97 × 10^6^	2.61 × 10^6^
	Rank	2	3	9	1	6	8	7	5	4
F18	Best	2.31 × 10^3^	1.40 × 10^4^	4.94 × 10^7^	2.83 × 10^5^	2.27 × 10^3^	4.23 × 10^4^	7.99 × 10^4^	9.31 × 10^4^	3.92 × 10^4^
	Ave	1.41 × 10^4^	1.32 × 10^6^	7.02 × 10^8^	6.46 × 10^5^	3.60 × 10^6^	1.19 × 10^7^	2.19 × 10^6^	8.70 × 10^5^	4.79 × 10^7^
	Std	1.45 × 10^4^	1.36 × 10^6^	5.04 × 10^8^	3.26 × 10^5^	9.16 × 10^6^	1.74 × 10^7^	1.75 × 10^6^	6.32 × 10^5^	8.42 × 10^7^
	Rank	1	4	9	2	6	7	5	3	8
F19	Best	2.22 × 10^3^	2.29 × 10^3^	2.50 × 10^3^	2.69 × 10^3^	2.25 × 10^3^	2.47 × 10^3^	2.25 × 10^3^	2.37 × 10^3^	2.33 × 10^3^
	Ave	2.47 × 10^3^	2.70 × 10^3^	2.88 × 10^3^	3.11 × 10^3^	2.72 × 10^3^	2.93 × 10^3^	2.56 × 10^3^	2.82 × 10^3^	2.61 × 10^3^
	Std	1.72 × 10^2^	2.06 × 10^2^	2.14 × 10^2^	2.48 × 10^2^	1.91 × 10^2^	1.81 × 10^2^	2.04 × 10^2^	2.23 × 10^2^	1.84 × 10^2^
	Rank	1	4	7	9	5	8	2	6	3
F20	Best	2.35 × 10^3^	2.43 × 10^3^	2.62 × 10^3^	2.56 × 10^3^	2.43 × 10^3^	2.55 × 10^3^	2.40 × 10^3^	2.43 × 10^3^	2.41 × 10^3^
	Ave	2.42 × 10^3^	2.53 × 10^3^	2.72 × 10^3^	2.69 × 10^3^	2.54 × 10^3^	2.63 × 10^3^	2.49 × 10^3^	2.57 × 10^3^	2.48 × 10^3^
	Std	3.19 × 10^1^	5.52 × 10^1^	6.18 × 10^1^	7.55 × 10^1^	5.13 × 10^1^	5.79 × 10^1^	3.98 × 10^1^	5.54 × 10^1^	4.70 × 10^1^
	Rank	1	4	9	8	5	7	3	6	2
F21	Best	2.30 × 10^3^	2.53 × 10^3^	7.08 × 10^3^	6.22 × 10^3^	2.33 × 10^3^	2.61 × 10^3^	2.43 × 10^3^	2.60 × 10^3^	2.83 × 10^3^
	Ave	5.23 × 10^3^	5.08 × 10^3^	9.13 × 10^3^	7.78 × 10^3^	5.13 × 10^3^	7.64 × 10^3^	2.60 × 10^3^	7.08 × 10^3^	5.36 × 10^3^
	Std	1.71 × 10^3^	2.19 × 10^3^	7.01 × 10^2^	8.29 × 10^2^	2.30 × 10^3^	1.85 × 10^3^	2.44 × 10^2^	1.17 × 10^3^	2.14 × 10^3^
	Rank	4	2	9	8	3	7	1	6	5
F22	Best	2.71 × 10^3^	2.84 × 10^3^	3.15 × 10^3^	3.17 × 10^3^	2.81 × 10^3^	2.98 × 10^3^	2.84 × 10^3^	2.96 × 10^3^	2.80 × 10^3^
	Ave	2.78 × 10^3^	2.93 × 10^3^	3.54 × 10^3^	3.49 × 10^3^	2.98 × 10^3^	3.18 × 10^3^	2.95 × 10^3^	3.19 × 10^3^	2.90 × 10^3^
	Std	4.52 × 10^1^	5.61 × 10^1^	1.41 × 10^2^	1.73 × 10^2^	8.36 × 10^1^	1.13 × 10^2^	5.75 × 10^1^	1.13 × 10^2^	6.83 × 10^1^
	Rank	1	3	9	8	5	6	4	7	2
F23	Best	2.95 × 10^3^	2.98 × 10^3^	3.58 × 10^3^	3.50 × 10^3^	3.00 × 10^3^	3.01 × 10^3^	2.99 × 10^3^	3.24 × 10^3^	2.93 × 10^3^
	Ave	3.08 × 10^3^	3.08 × 10^3^	4.20 × 10^3^	3.72 × 10^3^	3.15 × 10^3^	3.22 × 10^3^	3.09 × 10^3^	3.46 × 10^3^	3.11 × 10^3^
	Std	8.20 × 10^1^	5.90 × 10^1^	2.97 × 10^2^	1.05 × 10^2^	1.08 × 10^2^	9.20 × 10^1^	5.60 × 10^1^	1.47 × 10^2^	7.51 × 10^1^
	Rank	2	1	9	8	5	6	3	7	4
F24	Best	2.88 × 10^3^	3.03 × 10^3^	5.06 × 10^3^	2.88 × 10^3^	2.89 × 10^3^	3.02 × 10^3^	2.91 × 10^3^	2.91 × 10^3^	3.07 × 10^3^
	Ave	2.90 × 10^3^	3.16 × 10^3^	7.67 × 10^3^	2.92 × 10^3^	2.96 × 10^3^	3.11 × 10^3^	2.99 × 10^3^	2.95 × 10^3^	3.28 × 10^3^
	Std	1.35 × 10^1^	1.04 × 10^2^	7.93 × 10^2^	2.28 × 10^1^	6.07 × 10^1^	4.78 × 10^1^	3.11 × 10^1^	2.61 × 10^1^	2.23 × 10^2^
	Rank	1	7	9	2	4	6	5	3	8
F25	Best	2.81 × 10^3^	4.20 × 10^3^	9.71 × 10^3^	2.96 × 10^3^	5.57 × 10^3^	6.35 × 10^3^	3.32 × 10^3^	4.44 × 10^3^	4.76 × 10^3^
	Ave	4.95 × 10^3^	6.59 × 10^3^	1.24 × 10^4^	9.55 × 10^3^	6.64 × 10^3^	8.51 × 10^3^	5.68 × 10^3^	7.67 × 10^3^	6.00 × 10^3^
	Std	1.36 × 10^3^	1.23 × 10^3^	1.59 × 10^3^	3.43 × 10^3^	6.27 × 10^2^	9.00 × 10^2^	1.56 × 10^3^	1.10 × 10^3^	6.23 × 10^2^
	Rank	1	4	9	8	5	7	2	6	3
F26	Best	3.22 × 10^3^	3.27 × 10^3^	3.72 × 10^3^	3.51 × 10^3^	3.23 × 10^3^	3.25 × 10^3^	3.27 × 10^3^	3.28 × 10^3^	3.28 × 10^3^
	Ave	3.25 × 10^3^	3.37 × 10^3^	4.68 × 10^3^	4.13 × 10^3^	3.31 × 10^3^	3.43 × 10^3^	3.37 × 10^3^	3.50 × 10^3^	3.39 × 10^3^
	Std	2.08 × 10^1^	7.46 × 10^1^	4.60 × 10^2^	4.78 × 10^2^	6.53 × 10^1^	1.34 × 10^2^	5.69 × 10^1^	1.58 × 10^2^	7.34 × 10^1^
	Rank	1	3	9	8	2	6	4	7	5
F27	Best	3.20 × 10^3^	3.41 × 10^3^	6.89 × 10^3^	3.20 × 10^3^	3.25 × 10^3^	3.39 × 10^3^	3.32 × 10^3^	3.25 × 10^3^	3.57 × 10^3^
	Ave	3.23 × 10^3^	3.62 × 10^3^	9.15 × 10^3^	3.23 × 10^3^	3.45 × 10^3^	3.55 × 10^3^	3.48 × 10^3^	3.33 × 10^3^	4.01 × 10^3^
	Std	2.31 × 10^1^	2.09 × 10^2^	6.06 × 10^2^	3.48 × 10^1^	2.56 × 10^2^	1.13 × 10^2^	1.28 × 10^2^	3.34 × 10^1^	3.88 × 10^2^
	Rank	1	7	9	2	4	6	5	3	8
F28	Best	3.58 × 10^3^	3.87 × 10^3^	5.20 × 10^3^	4.28 × 10^3^	4.05 × 10^3^	4.34 × 10^3^	3.81 × 10^3^	3.96 × 10^3^	3.87 × 10^3^
	Ave	3.95 × 10^3^	4.59 × 10^3^	1.12 × 10^4^	5.06 × 10^3^	4.57 × 10^3^	5.18 × 10^3^	4.62 × 10^3^	4.65 × 10^3^	4.23 × 10^3^
	Std	2.35 × 10^2^	4.00 × 10^2^	6.77 × 10^3^	4.21 × 10^2^	2.96 × 10^2^	5.03 × 10^2^	3.46 × 10^2^	4.02 × 10^2^	2.17 × 10^2^
	Rank	1	4	9	7	3	8	5	6	2
F29	Best	2.55 × 10^4^	1.76 × 10^6^	1.67 × 10^9^	2.64 × 10^5^	1.62 × 10^4^	5.80 × 10^6^	1.10 × 10^6^	3.53 × 10^5^	3.05 × 10^6^
	Ave	1.16 × 10^5^	1.63 × 10^7^	3.84 × 10^9^	2.17 × 10^6^	3.89 × 10^6^	4.16 × 10^7^	2.25 × 10^7^	5.06 × 10^6^	3.51 × 10^7^
	Std	7.95 × 10^4^	1.51 × 10^7^	1.79 × 10^9^	1.26 × 10^6^	5.02 × 10^6^	3.40 × 10^7^	2.45 × 10^7^	4.25 × 10^6^	3.64 × 10^7^
	Rank	1	5	9	2	3	8	6	4	7

**Table 7:** Comparative results of different algorithms (Dim = 50).

Function	Index	MISCSO	SCSO	AOA	SSA	DBO	WOA	AO	HHO	GJO
F1	Best	5.25 × 10^5^	9.95 × 10^9^	1.18 × 10^11^	2.53 × 10^4^	2.16 × 10^8^	4.17 × 10^9^	2.30 × 10^9^	1.21 × 10^8^	2.36 × 10^10^
	Ave	1.76 × 10^6^	2.61 × 10^10^	1.30 × 10^11^	5.42 × 10^4^	1.76 × 10^9^	7.60 × 10^9^	5.19 × 10^9^	2.76 × 10^8^	3.67 × 10^10^
	Std	6.96 × 10^5^	6.63 × 10^9^	4.06 × 10^9^	1.62 × 10^4^	2.70 × 10^9^	2.34 × 10^9^	1.44 × 10^9^	1.06 × 10^8^	7.37 × 10^9^
	Rank	2	7	9	1	4	6	5	3	8
F2	Best	4.12 × 10^4^	7.28 × 10^4^	1.49 × 10^5^	1.32 × 10^5^	1.45 × 10^5^	1.62 × 10^5^	1.83 × 10^5^	1.03 × 10^5^	9.86 × 10^4^
	Ave	6.02 × 10^4^	1.17 × 10^5^	1.90 × 10^5^	2.31 × 10^5^	2.24 × 10^5^	2.74 × 10^5^	2.51 × 10^5^	1.39 × 10^5^	1.27 × 10^5^
	Std	9.72 × 10^3^	1.86 × 10^4^	2.13 × 10^4^	5.52 × 10^4^	5.79 × 10^4^	8.74 × 10^4^	4.56 × 10^4^	2.17 × 10^4^	1.69 × 10^4^
	Rank	1	2	5	7	6	9	8	4	3
F3	Best	4.75 × 10^2^	1.73 × 10^3^	4.33 × 10^4^	5.22 × 10^2^	5.90 × 10^2^	1.23 × 10^3^	1.09 × 10^3^	6.87 × 10^2^	2.87 × 10^3^
	Ave	5.49 × 10^2^	3.46 × 10^3^	5.21 × 10^4^	6.05 × 10^2^	9.35 × 10^2^	2.42 × 10^3^	1.63 × 10^3^	8.29 × 10^2^	5.44 × 10^3^
	Std	4.70 × 10^1^	1.21 × 10^3^	3.67 × 10^3^	8.05 × 10^1^	3.04 × 10^2^	6.89 × 10^2^	3.09 × 10^2^	9.17 × 10^1^	1.85 × 10^3^
	Rank	1	7	9	2	4	6	5	3	8
F4	Best	6.61 × 10^2^	8.83 × 10^2^	1.06 × 10^3^	8.75 × 10^2^	7.97 × 10^2^	9.22 × 10^2^	8.22 × 10^2^	8.62 × 10^2^	8.49 × 10^2^
	Ave	7.38 × 10^2^	9.44 × 10^2^	1.12 × 10^3^	1.06 × 10^3^	9.58 × 10^2^	1.08 × 10^3^	9.01 × 10^2^	9.02 × 10^2^	9.31 × 10^2^
	Std	6.43 × 10^1^	4.10 × 10^1^	3.35 × 10^1^	1.79 × 10^2^	1.01 × 10^2^	1.01 × 10^2^	4.06 × 10^1^	1.92 × 10^1^	6.96 × 10^1^
	Rank	1	5	9	7	6	8	2	3	4
F5	Best	6.01 × 10^2^	6.63 × 10^2^	6.63 × 10^2^	6.66 × 10^2^	6.43 × 10^2^	6.72 × 10^2^	6.57 × 10^2^	6.62 × 10^2^	6.38 × 10^2^
	Ave	6.04 × 10^2^	6.76 × 10^2^	6.76 × 10^2^	6.79 × 10^2^	6.65 × 10^2^	6.94 × 10^2^	6.67 × 10^2^	6.77 × 10^2^	6.53 × 10^2^
	Std	2.09 × 10^0^	6.18 × 10^0^	4.43 × 10^0^	9.87 × 10^0^	1.02 × 10^1^	1.30 × 10^1^	7.60 × 10^0^	4.83 × 10^0^	6.34 × 10^0^
	Rank	1	5	6	8	3	9	4	7	2
F6	Best	9.47 × 10^2^	1.42 × 10^3^	1.79 × 10^3^	1.78 × 10^3^	1.27 × 10^3^	1.50 × 10^3^	1.41 × 10^3^	1.69 × 10^3^	1.27 × 10^3^
	Ave	1.29 × 10^3^	1.63 × 10^3^	1.91 × 10^3^	3.20 × 10^3^	1.46 × 10^3^	1.84 × 10^3^	1.60 × 10^3^	1.86 × 10^3^	1.44 × 10^3^
	Std	3.11 × 10^2^	1.26 × 10^2^	4.90 × 10^1^	1.59 × 10^3^	1.49 × 10^2^	1.07 × 10^2^	9.19 × 10^1^	7.56 × 10^1^	1.06 × 10^2^
	Rank	1	5	8	9	3	6	4	7	2
F7	Best	9.24 × 10^2^	1.22 × 10^3^	1.37 × 10^3^	1.22 × 10^3^	1.14 × 10^3^	1.20 × 10^3^	1.17 × 10^3^	1.14 × 10^3^	1.12 × 10^3^
	Ave	1.02 × 10^3^	1.28 × 10^3^	1.43 × 10^3^	1.39 × 10^3^	1.30 × 10^3^	1.35 × 10^3^	1.23 × 10^3^	1.21 × 10^3^	1.23 × 10^3^
	Std	4.11 × 10^1^	3.26 × 10^1^	3.06 × 10^1^	1.46 × 10^2^	8.61 × 10^1^	1.18 × 10^2^	2.74 × 10^1^	3.03 × 10^1^	7.30 × 10^1^
	Rank	1	5	9	8	6	7	3	2	4
F8	Best	2.52 × 10^3^	1.49 × 10^4^	1.66 × 10^4^	1.32 × 10^4^	8.55 × 10^3^	1.81 × 10^4^	2.05 × 10^4^	2.26 × 10^4^	1.51 × 10^4^
	Ave	7.35 × 10^3^	2.03 × 10^4^	1.93 × 10^4^	1.99 × 10^4^	2.43 × 10^4^	3.51 × 10^4^	2.52 × 10^4^	2.84 × 10^4^	2.12 × 10^4^
	Std	3.41 × 10^3^	3.22 × 10^3^	1.46 × 10^3^	6.16 × 10^3^	7.95 × 10^3^	1.18 × 10^4^	3.38 × 10^3^	2.92 × 10^3^	4.23 × 10^3^
	Rank	1	4	2	3	6	9	7	8	5
F9	Best	4.70 × 10^3^	8.08 × 10^3^	1.04 × 10^4^	6.98 × 10^3^	7.48 × 10^3^	9.75 × 10^3^	7.13 × 10^3^	8.05 × 10^3^	8.31 × 10^3^
	Ave	5.94 × 10^3^	1.01 × 10^4^	1.22 × 10^4^	8.54 × 10^3^	1.04 × 10^4^	1.25 × 10^4^	9.97 × 10^3^	9.49 × 10^3^	1.11 × 10^4^
	Std	7.39 × 10^2^	7.55 × 10^2^	8.05 × 10^2^	6.46 × 10^2^	1.87 × 10^3^	1.17 × 10^3^	1.27 × 10^3^	7.23 × 10^2^	2.69 × 10^3^
	Rank	1	5	8	2	6	9	4	3	7
F10	Best	1.17 × 10^3^	3.20 × 10^3^	2.23 × 10^4^	1.29 × 10^3^	1.52 × 10^3^	3.11 × 10^3^	2.41 × 10^3^	1.50 × 10^3^	4.92 × 10^3^
	Ave	1.34 × 10^3^	6.87 × 10^3^	2.77 × 10^4^	1.54 × 10^3^	2.73 × 10^3^	5.34 × 10^3^	3.59 × 10^3^	1.81 × 10^3^	9.16 × 10^3^
	Std	2.01 × 10^2^	2.20 × 10^3^	1.65 × 10^3^	1.48 × 10^2^	1.49 × 10^3^	1.42 × 10^3^	7.59 × 10^2^	1.44 × 10^2^	2.63 × 10^3^
	Rank	1	7	9	2	4	6	5	3	8
F11	Best	3.58 × 10^6^	2.30 × 10^8^	7.79 × 10^10^	4.36 × 10^6^	4.56 × 10^7^	4.49 × 10^8^	2.05 × 10^8^	5.96 × 10^7^	1.29 × 10^9^
	Ave	1.35 × 10^7^	3.43 × 10^9^	1.15 × 10^11^	2.76 × 10^7^	6.15 × 10^8^	1.95 × 10^9^	9.83 × 10^8^	2.30 × 10^8^	8.28 × 10^9^
	Std	7.44 × 10^6^	2.95 × 10^9^	1.79 × 10^10^	1.99 × 10^7^	7.06 × 10^8^	9.27 × 10^8^	5.80 × 10^8^	1.11 × 10^8^	4.94 × 10^9^
	Rank	1	7	9	2	4	6	5	3	8
F12	Best	1.25 × 10^4^	2.43 × 10^7^	1.60 × 10^10^	2.71 × 10^4^	2.90 × 10^5^	2.09 × 10^7^	1.30 × 10^7^	1.77 × 10^6^	7.75 × 10^7^
	Ave	1.01 × 10^5^	4.29 × 10^8^	6.44 × 10^10^	1.90 × 10^5^	5.02 × 10^7^	1.30 × 10^8^	7.60 × 10^7^	4.92 × 10^6^	2.32 × 10^9^
	Std	6.45 × 10^4^	8.25 × 10^8^	2.04 × 10^10^	1.28 × 10^5^	6.87 × 10^7^	1.10 × 10^8^	8.89 × 10^7^	1.97 × 10^6^	3.09 × 10^9^
	Rank	1	7	9	2	4	6	5	3	8
F13	Best	1.09 × 10^5^	3.80 × 10^4^	2.82 × 10^7^	2.49 × 10^4^	9.48 × 10^4^	5.72 × 10^5^	1.88 × 10^5^	3.45 × 10^4^	2.46 × 10^5^
	Ave	6.81 × 10^5^	1.51 × 10^6^	1.79 × 10^8^	1.14 × 10^5^	2.93 × 10^6^	5.22 × 10^6^	5.43 × 10^6^	2.30 × 10^6^	2.72 × 10^6^
	Std	5.94 × 10^5^	1.45 × 10^6^	1.12 × 10^8^	7.07 × 10^4^	3.66 × 10^6^	3.66 × 10^6^	5.15 × 10^6^	2.21 × 10^6^	4.28 × 10^6^
	Rank	2	3	9	1	6	7	8	4	5
F14	Best	2.68 × 10^3^	3.04 × 10^4^	4.02 × 10^9^	1.42 × 10^4^	2.66 × 10^4^	3.37 × 10^5^	3.97 × 10^5^	2.95 × 10^5^	7.32 × 10^4^
	Ave	2.33 × 10^4^	5.01 × 10^7^	1.46 × 10^10^	5.28 × 10^4^	1.47 × 10^7^	9.38 × 10^6^	1.26 × 10^6^	7.26 × 10^5^	3.50 × 10^8^
	Std	1.66 × 10^4^	1.20 × 10^8^	4.90 × 10^9^	4.83 × 10^4^	4.50 × 10^7^	1.16 × 10^7^	7.89 × 10^5^	2.95 × 10^5^	4.38 × 10^8^
	Rank	1	7	9	2	6	5	4	3	8
F15	Best	2.92 × 10^3^	3.81 × 10^3^	6.65 × 10^3^	3.48 × 10^3^	3.22 × 10^3^	4.82 × 10^3^	3.25 × 10^3^	3.73 × 10^3^	3.17 × 10^3^
	Ave	3.72 × 10^3^	4.62 × 10^3^	1.09 × 10^4^	4.73 × 10^3^	4.78 × 10^3^	6.00 × 10^3^	4.59 × 10^3^	4.70 × 10^3^	4.30 × 10^3^
	Std	4.42 × 10^2^	4.80 × 10^2^	2.25 × 10^3^	5.63 × 10^2^	6.19 × 10^2^	7.55 × 10^2^	6.33 × 10^2^	5.98 × 10^2^	6.51 × 10^2^
	Rank	1	4	9	6	7	8	3	5	2
F16	Best	2.45 × 10^3^	3.09 × 10^3^	9.82 × 10^3^	3.17 × 10^3^	3.00 × 10^3^	3.51 × 10^3^	3.02 × 10^3^	2.87 × 10^3^	2.78 × 10^3^
	Ave	3.26 × 10^3^	3.91 × 10^3^	4.42 × 10^4^	3.90 × 10^3^	4.12 × 10^3^	4.59 × 10^3^	3.92 × 10^3^	3.74 × 10^3^	3.62 × 10^3^
	Std	4.65 × 10^2^	4.45 × 10^2^	1.81 × 10^4^	4.59 × 10^2^	4.92 × 10^2^	6.91 × 10^2^	4.56 × 10^2^	3.63 × 10^2^	3.95 × 10^2^
	Rank	1	5	9	4	7	8	6	3	2
F17	Best	7.43 × 10^5^	5.13 × 10^5^	1.28 × 10^8^	5.02 × 10^5^	8.34 × 10^5^	4.78 × 10^6^	1.49 × 10^6^	8.75 × 10^5^	1.52 × 10^6^
	Ave	4.75 × 10^6^	1.46 × 10^7^	4.47 × 10^8^	1.79 × 10^6^	8.79 × 10^6^	3.70 × 10^7^	1.60 × 10^7^	5.57 × 10^6^	1.72 × 10^7^
	Std	2.65 × 10^6^	2.45 × 10^7^	3.09 × 10^8^	1.16 × 10^6^	9.00 × 10^6^	2.92 × 10^7^	1.00 × 10^7^	4.09 × 10^6^	2.52 × 10^7^
	Rank	2	5	9	1	4	8	6	3	7
F18	Best	2.71 × 10^3^	1.07 × 10^5^	3.35 × 10^9^	5.54 × 10^4^	1.95 × 10^4^	7.24 × 10^5^	2.29 × 10^5^	3.01 × 10^5^	1.24 × 10^5^
	Ave	2.22 × 10^4^	1.40 × 10^7^	8.61 × 10^9^	1.51 × 10^6^	5.96 × 10^6^	1.02 × 10^7^	2.19 × 10^6^	1.46 × 10^6^	2.94 × 10^8^
	Std	1.21 × 10^4^	2.87 × 10^7^	2.07 × 10^9^	1.30 × 10^6^	7.16 × 10^6^	9.77 × 10^6^	2.38 × 10^6^	1.24 × 10^6^	4.14 × 10^8^
	Rank	1	7	9	3	5	6	4	2	8
F19	Best	2.68 × 10^3^	2.72 × 10^3^	3.44 × 10^3^	3.26 × 10^3^	2.69 × 10^3^	3.46 × 10^3^	3.00 × 10^3^	2.80 × 10^3^	2.52 × 10^3^
	Ave	3.26 × 10^3^	3.54 × 10^3^	4.10 × 10^3^	3.97 × 10^3^	3.71 × 10^3^	3.92 × 10^3^	3.39 × 10^3^	3.40 × 10^3^	3.32 × 10^3^
	Std	3.43 × 10^2^	4.52 × 10^2^	3.54 × 10^2^	4.20 × 10^2^	3.60 × 10^2^	2.69 × 10^2^	2.43 × 10^2^	3.02 × 10^2^	3.93 × 10^2^
	Rank	1	5	9	8	6	7	3	4	2
F20	Best	2.46 × 10^3^	2.65 × 10^3^	2.92 × 10^3^	2.93 × 10^3^	2.65 × 10^3^	2.89 × 10^3^	2.63 × 10^3^	2.81 × 10^3^	2.60 × 10^3^
	Ave	2.55 × 10^3^	2.78 × 10^3^	3.13 × 10^3^	3.15 × 10^3^	2.82 × 10^3^	3.07 × 10^3^	2.74 × 10^3^	2.92 × 10^3^	2.69 × 10^3^
	Std	4.96 × 10^1^	6.43 × 10^1^	1.17 × 10^2^	1.02 × 10^2^	8.64 × 10^1^	1.14 × 10^2^	6.68 × 10^1^	7.66 × 10^1^	4.37 × 10^1^
	Rank	1	4	8	9	5	7	3	6	2
F21	Best	6.91 × 10^3^	9.23 × 10^3^	1.44 × 10^4^	9.58 × 10^3^	8.78 × 10^3^	1.17 × 10^4^	6.65 × 10^3^	9.73 × 10^3^	9.85 × 10^3^
	Ave	8.30 × 10^3^	1.21 × 10^4^	1.58 × 10^4^	1.09 × 10^4^	1.16 × 10^4^	1.41 × 10^4^	1.18 × 10^4^	1.20 × 10^4^	1.32 × 10^4^
	Std	8.73 × 10^2^	1.22 × 10^3^	6.65 × 10^2^	7.41 × 10^2^	1.56 × 10^3^	1.18 × 10^3^	1.63 × 10^3^	1.25 × 10^3^	2.49 × 10^3^
	Rank	1	6	9	2	3	8	4	5	7
F22	Best	2.90 × 10^3^	3.20 × 10^3^	3.90 × 10^3^	3.90 × 10^3^	3.26 × 10^3^	3.37 × 10^3^	3.36 × 10^3^	3.63 × 10^3^	3.16 × 10^3^
	Ave	3.02 × 10^3^	3.39 × 10^3^	4.34 × 10^3^	4.31 × 10^3^	3.50 × 10^3^	3.83 × 10^3^	3.55 × 10^3^	3.97 × 10^3^	3.32 × 10^3^
	Std	6.31 × 10^1^	1.21 × 10^2^	2.69 × 10^2^	2.42 × 10^2^	1.46 × 10^2^	2.36 × 10^2^	1.15 × 10^2^	1.83 × 10^2^	9.64 × 10^1^
	Rank	1	3	9	8	4	6	5	7	2
F23	Best	3.26 × 10^3^	3.28 × 10^3^	4.05 × 10^3^	4.04 × 10^3^	3.41 × 10^3^	3.54 × 10^3^	3.22 × 10^3^	3.79 × 10^3^	3.33 × 10^3^
	Ave	3.45 × 10^3^	3.51 × 10^3^	5.25 × 10^3^	4.27 × 10^3^	3.69 × 10^3^	3.83 × 10^3^	3.57 × 10^3^	4.25 × 10^3^	3.47 × 10^3^
	Std	1.15 × 10^2^	1.08 × 10^2^	5.70 × 10^2^	1.25 × 10^2^	1.31 × 10^2^	1.59 × 10^2^	1.33 × 10^2^	2.52 × 10^2^	1.03 × 10^2^
	Rank	1	3	9	8	5	6	4	7	2
F24	Best	3.03 × 10^3^	3.56 × 10^3^	1.64 × 10^4^	3.07 × 10^3^	3.05 × 10^3^	3.52 × 10^3^	3.38 × 10^3^	3.17 × 10^3^	4.38 × 10^3^
	Ave	3.10 × 10^3^	4.82 × 10^3^	1.87 × 10^4^	3.12 × 10^3^	3.49 × 10^3^	4.18 × 10^3^	3.70 × 10^3^	3.28 × 10^3^	5.65 × 10^3^
	Std	2.69 × 10^1^	7.32 × 10^2^	8.68 × 10^2^	3.09 × 10^1^	1.16 × 10^3^	3.34 × 10^2^	2.27 × 10^2^	7.37 × 10^1^	6.93 × 10^2^
	Rank	1	7	9	2	4	6	5	3	8
F25	Best	2.93 × 10^3^	8.77 × 10^3^	1.63 × 10^4^	3.71 × 10^3^	7.42 × 10^3^	1.16 × 10^4^	5.65 × 10^3^	8.12 × 10^3^	7.96 × 10^3^
	Ave	7.45 × 10^3^	1.13 × 10^4^	1.88 × 10^4^	1.34 × 10^4^	9.94 × 10^3^	1.42 × 10^4^	9.39 × 10^3^	1.13 × 10^4^	9.92 × 10^3^
	Std	1.28 × 10^3^	1.23 × 10^3^	6.06 × 10^2^	5.68 × 10^3^	1.48 × 10^3^	1.49 × 10^3^	2.21 × 10^3^	1.13 × 10^3^	8.27 × 10^2^
	Rank	1	6	9	7	4	8	2	5	3
F26	Best	3.37 × 10^3^	3.79 × 10^3^	5.10 × 10^3^	4.23 × 10^3^	3.64 × 10^3^	3.72 × 10^3^	3.81 × 10^3^	3.80 × 10^3^	3.83 × 10^3^
	Ave	3.53 × 10^3^	4.22 × 10^3^	6.58 × 10^3^	5.67 × 10^3^	3.93 × 10^3^	4.53 × 10^3^	4.22 × 10^3^	4.46 × 10^3^	4.12 × 10^3^
	Std	1.36 × 10^2^	2.53 × 10^2^	8.53 × 10^2^	6.29 × 10^2^	2.61 × 10^2^	4.35 × 10^2^	2.16 × 10^2^	3.66 × 10^2^	2.16 × 10^2^
	Rank	1	4	9	8	2	7	5	6	3
F27	Best	3.28 × 10^3^	4.47 × 10^3^	1.27 × 10^4^	3.33 × 10^3^	3.47 × 10^3^	4.17 × 10^3^	4.08 × 10^3^	3.58 × 10^3^	4.87 × 10^3^
	Ave	3.35 × 10^3^	5.32 × 10^3^	1.72 × 10^4^	3.54 × 10^3^	4.92 × 10^3^	5.09 × 10^3^	4.81 × 10^3^	3.83 × 10^3^	6.13 × 10^3^
	Std	4.81 × 10^1^	6.05 × 10^2^	1.84 × 10^3^	2.75 × 10^2^	1.85 × 10^3^	3.98 × 10^2^	3.57 × 10^2^	1.92 × 10^2^	6.38 × 10^2^
	Rank	1	7	9	2	5	6	4	3	8
F28	Best	3.78 × 10^3^	4.99 × 10^3^	2.63 × 10^4^	5.05 × 10^3^	4.80 × 10^3^	6.33 × 10^3^	5.63 × 10^3^	4.87 × 10^3^	5.06 × 10^3^
	Ave	4.62 × 10^3^	6.63 × 10^3^	5.19 × 10^5^	6.62 × 10^3^	6.47 × 10^3^	8.98 × 10^3^	6.81 × 10^3^	6.20 × 10^3^	6.19 × 10^3^
	Std	3.51 × 10^2^	8.53 × 10^2^	8.44 × 10^5^	8.62 × 10^2^	1.07 × 10^3^	1.46 × 10^3^	9.45 × 10^2^	8.40 × 10^2^	9.41 × 10^2^
	Rank	1	6	9	5	4	8	7	3	2
F29	Best	8.79 × 10^5^	7.44 × 10^7^	4.27 × 10^9^	3.41 × 10^7^	4.17 × 10^6^	9.44 × 10^7^	6.29 × 10^7^	3.95 × 10^7^	1.37 × 10^8^
	Ave	2.77 × 10^6^	2.20 × 10^8^	1.32 × 10^10^	4.75 × 10^7^	4.88 × 10^7^	2.53 × 10^8^	1.41 × 10^8^	7.41 × 10^7^	4.15 × 10^8^
	Std	1.77 × 10^6^	1.92 × 10^8^	4.81 × 10^9^	1.02 × 10^7^	1.04 × 10^8^	1.03 × 10^8^	5.81 × 10^7^	2.38 × 10^7^	2.31 × 10^8^
	Rank	1	6	9	2	3	7	5	4	8

**Table 8:** Comparative results of different algorithms (Dim = 100).

Function	Index	MISCSO	SCSO	AOA	SSA	DBO	WOA	AO	HHO	GJO
F1	Best	1.12 × 10^7^	7.62 × 10^10^	2.53 × 10^11^	9.15 × 10^5^	4.88 × 10^9^	4.49 × 10^10^	2.84 × 10^10^	5.09 × 10^9^	1.04 × 10^11^
	Ave	1.52 × 10^7^	9.75 × 10^10^	2.79 × 10^11^	1.80 × 10^9^	4.62 × 10^10^	6.30 × 10^10^	4.20 × 10^10^	7.12 × 10^9^	1.35 × 10^11^
	Std	3.22 × 10^6^	1.35 × 10^10^	9.93 × 10^9^	3.02 × 10^9^	5.46 × 10^10^	8.54 × 10^9^	6.57 × 10^9^	1.19 × 10^9^	1.39 × 10^10^
	Rank	1	7	9	2	5	6	4	3	8
F2	Best	1.94 × 10^5^	2.65 × 10^5^	3.38 × 10^5^	5.76 × 10^5^	3.39 × 10^5^	4.62 × 10^5^	3.22 × 10^5^	2.81 × 10^5^	2.77 × 10^5^
	Ave	2.30 × 10^5^	3.05 × 10^5^	3.54 × 10^5^	7.41 × 10^5^	4.91 × 10^5^	8.52 × 10^5^	3.48 × 10^5^	3.22 × 10^5^	3.28 × 10^5^
	Std	1.61 × 10^4^	1.95 × 10^4^	1.15 × 10^4^	1.05 × 10^5^	1.69 × 10^5^	1.27 × 10^5^	1.23 × 10^4^	1.98 × 10^4^	2.60 × 10^4^
	Rank	1	2	6	8	7	9	5	3	4
F3	Best	6.26 × 10^2^	6.63 × 10^3^	9.12 × 10^4^	7.39 × 10^2^	2.26 × 10^3^	6.78 × 10^3^	5.26 × 10^3^	2.04 × 10^3^	8.16 × 10^3^
	Ave	7.65 × 10^2^	1.13 × 10^4^	1.38 × 10^5^	1.18 × 10^3^	8.48 × 10^3^	1.14 × 10^4^	7.77 × 10^3^	2.60 × 10^3^	1.93 × 10^4^
	Std	7.65 × 10^1^	3.05 × 10^3^	1.57 × 10^4^	3.76 × 10^2^	1.08 × 10^4^	2.45 × 10^3^	1.64 × 10^3^	3.44 × 10^2^	4.96 × 10^3^
	Rank	1	6	9	2	5	7	4	3	8
F4	Best	9.77 × 10^2^	1.38 × 10^3^	1.79 × 10^3^	1.31 × 10^3^	1.26 × 10^3^	1.64 × 10^3^	1.50 × 10^3^	1.51 × 10^3^	1.46 × 10^3^
	Ave	1.15 × 10^3^	1.61 × 10^3^	1.90 × 10^3^	1.92 × 10^3^	1.65 × 10^3^	1.82 × 10^3^	1.60 × 10^3^	1.60 × 10^3^	1.59 × 10^3^
	Std	1.18 × 10^2^	8.45 × 10^1^	5.02 × 10^1^	4.19 × 10^2^	2.53 × 10^2^	1.46 × 10^2^	6.77 × 10^1^	6.34 × 10^1^	1.07 × 10^2^
	Rank	1	5	8	9	6	7	3	4	2
F5	Best	6.02 × 10^2^	6.77 × 10^2^	6.72 × 10^2^	6.65 × 10^2^	6.54 × 10^2^	6.82 × 10^2^	6.67 × 10^2^	6.79 × 10^2^	6.63 × 10^2^
	Ave	6.03 × 10^2^	6.85 × 10^2^	6.79 × 10^2^	6.82 × 10^2^	6.73 × 10^2^	7.01 × 10^2^	6.83 × 10^2^	6.88 × 10^2^	6.74 × 10^2^
	Std	8.15 × 10^−1^	5.97 × 10^0^	4.36 × 10^0^	1.24 × 10^1^	1.17 × 10^1^	1.18 × 10^1^	5.54 × 10^0^	4.18 × 10^0^	5.29 × 10^0^
	Rank	1	7	4	5	2	9	6	8	3
F6	Best	1.47 × 10^3^	2.79 × 10^3^	3.42 × 10^3^	3.08 × 10^3^	2.15 × 10^3^	3.48 × 10^3^	3.05 × 10^3^	3.54 × 10^3^	2.65 × 10^3^
	Ave	2.48 × 10^3^	3.20 × 10^3^	3.62 × 10^3^	7.84 × 10^3^	2.76 × 10^3^	3.66 × 10^3^	3.32 × 10^3^	3.75 × 10^3^	3.02 × 10^3^
	Std	6.34 × 10^2^	1.96 × 10^2^	9.21 × 10^1^	3.53 × 10^3^	2.33 × 10^2^	9.02 × 10^1^	1.27 × 10^2^	1.19 × 10^2^	1.89 × 10^2^
	Rank	1	4	6	9	2	7	5	8	3
F7	Best	1.24 × 10^3^	1.90 × 10^3^	2.22 × 10^3^	1.83 × 10^3^	1.74 × 10^3^	2.04 × 10^3^	1.91 × 10^3^	1.92 × 10^3^	1.80 × 10^3^
	Ave	1.46 × 10^3^	2.07 × 10^3^	2.34 × 10^3^	2.36 × 10^3^	2.08 × 10^3^	2.29 × 10^3^	2.04 × 10^3^	2.07 × 10^3^	1.94 × 10^3^
	Std	1.20 × 10^2^	9.05 × 10^1^	6.39 × 10^1^	3.76 × 10^2^	2.41 × 10^2^	1.07 × 10^2^	5.54 × 10^1^	5.30 × 10^1^	1.29 × 10^2^
	Rank	1	5	8	9	6	7	3	4	2
F8	Best	1.42 × 10^4^	3.08 × 10^4^	4.05 × 10^4^	2.48 × 10^4^	2.50 × 10^4^	5.38 × 10^4^	4.76 × 10^4^	5.79 × 10^4^	3.73 × 10^4^
	Ave	2.23 × 10^4^	4.22 × 10^4^	4.75 × 10^4^	4.56 × 10^4^	6.56 × 10^4^	7.13 × 10^4^	6.18 × 10^4^	6.47 × 10^4^	6.06 × 10^4^
	Std	4.97 × 10^3^	6.03 × 10^3^	2.68 × 10^3^	1.52 × 10^4^	1.61 × 10^4^	1.49 × 10^4^	6.37 × 10^3^	5.03 × 10^3^	1.16 × 10^4^
	Rank	1	2	4	3	8	9	6	7	5
F9	Best	9.90 × 10^3^	1.77 × 10^4^	2.51 × 10^4^	1.41 × 10^4^	1.71 × 10^4^	2.57 × 10^4^	1.93 × 10^4^	1.96 × 10^4^	1.82 × 10^4^
	Ave	1.25 × 10^4^	2.20 × 10^4^	2.62 × 10^4^	1.64 × 10^4^	2.76 × 10^4^	2.79 × 10^4^	2.35 × 10^4^	2.30 × 10^4^	2.39 × 10^4^
	Std	1.24 × 10^3^	1.98 × 10^3^	8.47 × 10^2^	1.23 × 10^3^	5.24 × 10^3^	1.35 × 10^3^	1.97 × 10^3^	1.98 × 10^3^	4.97 × 10^3^
	Rank	1	3	7	2	8	9	5	4	6
F10	Best	3.68 × 10^3^	3.55 × 10^4^	1.33 × 10^5^	1.63 × 10^4^	9.77 × 10^4^	1.29 × 10^5^	1.49 × 10^5^	3.05 × 10^4^	7.21 × 10^4^
	Ave	9.83 × 10^3^	7.75 × 10^4^	1.21 × 10^6^	4.44 × 10^4^	1.70 × 10^5^	2.17 × 10^5^	2.67 × 10^5^	7.26 × 10^4^	9.72 × 10^4^
	Std	3.45 × 10^3^	1.86 × 10^4^	5.52 × 10^6^	1.63 × 10^4^	5.80 × 10^4^	1.04 × 10^5^	7.05 × 10^4^	2.16 × 10^4^	1.99 × 10^4^
	Rank	1	4	9	2	6	7	8	3	5
F11	Best	1.39 × 10^7^	6.87 × 10^9^	2.21 × 10^11^	1.24 × 10^8^	1.36 × 10^9^	8.28 × 10^9^	5.22 × 10^9^	6.51 × 10^8^	2.98 × 10^10^
	Ave	5.54 × 10^7^	2.82 × 10^10^	2.49 × 10^11^	3.16 × 10^8^	3.18 × 10^9^	1.33 × 10^10^	1.14 × 10^10^	1.45 × 10^9^	4.98 × 10^10^
	Std	2.53 × 10^7^	1.22 × 10^10^	8.21 × 10^9^	2.06 × 10^8^	1.13 × 10^9^	3.54 × 10^9^	3.74 × 10^9^	4.25 × 10^8^	1.15 × 10^10^
	Rank	1	7	9	2	4	6	5	3	8
F12	Best	7.75 × 10^4^	1.26 × 10^8^	4.30 × 10^10^	2.62 × 10^4^	1.09 × 10^7^	2.66 × 10^8^	9.96 × 10^7^	1.30 × 10^7^	2.65 × 10^9^
	Ave	1.72 × 10^5^	5.71 × 10^9^	6.05 × 10^10^	7.78 × 10^4^	1.49 × 10^8^	6.41 × 10^8^	2.96 × 10^8^	1.84 × 10^7^	8.40 × 10^9^
	Std	5.97 × 10^4^	3.85 × 10^9^	5.04 × 10^9^	4.19 × 10^4^	1.41 × 10^8^	2.95 × 10^8^	1.67 × 10^8^	4.04 × 10^6^	3.11 × 10^9^
	Rank	2	7	9	1	4	6	5	3	8
F13	Best	1.15 × 10^6^	2.39 × 10^6^	4.56 × 10^7^	4.95 × 10^5^	1.22 × 10^6^	3.98 × 10^6^	5.71 × 10^6^	2.14 × 10^6^	3.63 × 10^6^
	Ave	3.41 × 10^6^	8.39 × 10^6^	3.72 × 10^8^	1.43 × 10^6^	1.26 × 10^7^	1.53 × 10^7^	1.47 × 10^7^	5.92 × 10^6^	1.38 × 10^7^
	Std	1.81 × 10^6^	4.25 × 10^6^	3.16 × 10^8^	7.05 × 10^5^	8.23 × 10^6^	6.83 × 10^6^	5.40 × 10^6^	2.42 × 10^6^	5.59 × 10^6^
	Rank	2	4	9	1	5	8	7	3	6
F14	Best	9.37 × 10^3^	2.44 × 10^7^	2.72 × 10^10^	1.82 × 10^4^	7.85 × 10^4^	1.44 × 10^7^	6.23 × 10^6^	2.37 × 10^6^	2.42 × 10^7^
	Ave	6.78 × 10^4^	9.32 × 10^8^	3.59 × 10^10^	7.00 × 10^4^	2.57 × 10^7^	8.61 × 10^7^	2.76 × 10^7^	4.34 × 10^6^	2.99 × 10^9^
	Std	3.50 × 10^4^	1.15 × 10^9^	3.50 × 10^9^	4.65 × 10^4^	6.92 × 10^7^	6.36 × 10^7^	1.68 × 10^7^	9.64 × 10^5^	2.24 × 10^9^
	Rank	1	7	9	2	4	6	5	3	8
F15	Best	4.63 × 10^3^	7.56 × 10^3^	2.14 × 10^4^	5.85 × 10^3^	6.40 × 10^3^	1.03 × 10^4^	7.87 × 10^3^	6.86 × 10^3^	6.91 × 10^3^
	Ave	6.04 × 10^3^	9.78 × 10^3^	2.91 × 10^4^	8.07 × 10^3^	9.29 × 10^3^	1.48 × 10^4^	9.96 × 10^3^	8.83 × 10^3^	9.16 × 10^3^
	Std	5.25 × 10^2^	9.94 × 10^2^	4.35 × 10^3^	1.33 × 10^3^	1.53 × 10^3^	2.36 × 10^3^	1.06 × 10^3^	8.58 × 10^2^	1.22 × 10^3^
	Rank	1	6	9	2	5	8	7	3	4
F16	Best	3.27 × 10^3^	5.92 × 10^3^	2.44 × 10^6^	4.52 × 10^3^	6.09 × 10^3^	7.28 × 10^3^	5.87 × 10^3^	5.84 × 10^3^	5.88 × 10^3^
	Ave	4.79 × 10^3^	1.34 × 10^4^	3.85 × 10^7^	5.91 × 10^3^	8.59 × 10^3^	1.14 × 10^4^	9.15 × 10^3^	6.82 × 10^3^	2.14 × 10^4^
	Std	5.40 × 10^2^	1.18 × 10^4^	2.75 × 10^7^	7.72 × 10^2^	1.26 × 10^3^	2.83 × 10^3^	2.06 × 10^3^	5.73 × 10^2^	2.82 × 10^4^
	Rank	1	7	9	2	4	6	5	3	8
F17	Best	1.74 × 10^6^	2.37 × 10^6^	1.54 × 10^8^	5.28 × 10^5^	3.92 × 10^6^	5.49 × 10^6^	2.56 × 10^6^	3.40 × 10^6^	3.76 × 10^6^
	Ave	5.19 × 10^6^	7.73 × 10^6^	8.10 × 10^8^	1.90 × 10^6^	1.56 × 10^7^	1.33 × 10^7^	1.30 × 10^7^	7.14 × 10^6^	1.66 × 10^7^
	Std	2.63 × 10^6^	3.96 × 10^6^	3.28 × 10^8^	1.21 × 10^6^	1.02 × 10^7^	5.50 × 10^6^	5.59 × 10^6^	2.70 × 10^6^	1.29 × 10^7^
	Rank	2	4	9	1	7	6	5	3	8
F18	Best	1.36 × 10^4^	2.99 × 10^7^	2.36 × 10^10^	4.31 × 10^5^	2.67 × 10^6^	3.71 × 10^7^	8.32 × 10^6^	5.87 × 10^6^	2.79 × 10^8^
	Ave	5.45 × 10^4^	1.23 × 10^9^	3.47 × 10^10^	6.75 × 10^6^	3.83 × 10^7^	1.89 × 10^8^	3.65 × 10^7^	1.65 × 10^7^	2.63 × 10^9^
	Std	3.44 × 10^4^	1.45 × 10^9^	4.59 × 10^9^	4.26 × 10^6^	3.34 × 10^7^	2.51 × 10^8^	2.15 × 10^7^	7.60 × 10^6^	1.66 × 10^9^
	Rank	1	7	9	2	5	6	4	3	8
F19	Best	4.12 × 10^3^	5.21 × 10^3^	6.91 × 10^3^	5.30 × 10^3^	5.43 × 10^3^	5.87 × 10^3^	4.99 × 10^3^	5.31 × 10^3^	4.68 × 10^3^
	Ave	5.07 × 10^3^	6.24 × 10^3^	7.79 × 10^3^	6.36 × 10^3^	6.88 × 10^3^	6.98 × 10^3^	5.81 × 10^3^	6.14 × 10^3^	6.23 × 10^3^
	Std	5.50 × 10^2^	5.38 × 10^2^	4.62 × 10^2^	4.87 × 10^2^	8.33 × 10^2^	6.00 × 10^2^	6.16 × 10^2^	4.44 × 10^2^	1.00 × 10^3^
	Rank	1	5	9	6	7	8	2	3	4
F20	Best	2.79 × 10^3^	3.48 × 10^3^	3.96 × 10^3^	4.08 × 10^3^	3.74 × 10^3^	3.98 × 10^3^	3.75 × 10^3^	3.80 × 10^3^	3.32 × 10^3^
	Ave	2.94 × 10^3^	3.69 × 10^3^	4.21 × 10^3^	4.50 × 10^3^	3.99 × 10^3^	4.38 × 10^3^	4.13 × 10^3^	4.25 × 10^3^	3.52 × 10^3^
	Std	8.31 × 10^1^	1.28 × 10^2^	1.75 × 10^2^	1.92 × 10^2^	1.45 × 10^2^	2.24 × 10^2^	2.63 × 10^2^	2.06 × 10^2^	1.20 × 10^2^
	Rank	1	3	6	9	4	8	5	7	2
F21	Best	1.39 × 10^4^	2.18 × 10^4^	2.81 × 10^4^	1.76 × 10^4^	1.97 × 10^4^	2.80 × 10^4^	2.29 × 10^4^	2.31 × 10^4^	2.24 × 10^4^
	Ave	1.63 × 10^4^	2.47 × 10^4^	3.00 × 10^4^	1.97 × 10^4^	2.69 × 10^4^	3.05 × 10^4^	2.63 × 10^4^	2.60 × 10^4^	2.83 × 10^4^
	Std	1.26 × 10^3^	1.43 × 10^3^	9.46 × 10^2^	1.21 × 10^3^	5.64 × 10^3^	1.73 × 10^3^	1.62 × 10^3^	1.70 × 10^3^	4.90 × 10^3^
	Rank	1	3	8	2	6	9	5	4	7
F22	Best	3.09 × 10^3^	4.11 × 10^3^	4.74 × 10^3^	4.87 × 10^3^	4.36 × 10^3^	4.59 × 10^3^	4.36 × 10^3^	4.73 × 10^3^	4.11 × 10^3^
	Ave	3.21 × 10^3^	4.41 × 10^3^	5.70 × 10^3^	5.76 × 10^3^	4.84 × 10^3^	5.12 × 10^3^	4.75 × 10^3^	5.55 × 10^3^	4.43 × 10^3^
	Std	7.24 × 10^1^	1.82 × 10^2^	4.95 × 10^2^	3.99 × 10^2^	2.88 × 10^2^	2.16 × 10^2^	2.57 × 10^2^	3.82 × 10^2^	1.70 × 10^2^
	Rank	1	2	8	9	5	6	4	7	3
F23	Best	3.75 × 10^3^	4.92 × 10^3^	8.42 × 10^3^	6.18 × 10^3^	4.97 × 10^3^	5.60 × 10^3^	5.30 × 10^3^	6.48 × 10^3^	5.41 × 10^3^
	Ave	3.94 × 10^3^	5.42 × 10^3^	1.25 × 10^4^	6.89 × 10^3^	5.94 × 10^3^	6.46 × 10^3^	6.34 × 10^3^	7.56 × 10^3^	5.95 × 10^3^
	Std	1.14 × 10^2^	3.06 × 10^2^	1.66 × 10^3^	2.98 × 10^2^	5.31 × 10^2^	4.12 × 10^2^	4.91 × 10^2^	6.62 × 10^2^	3.95 × 10^2^
	Rank	1	2	9	7	3	6	5	8	4
F24	Best	3.28 × 10^3^	7.44 × 10^3^	3.06 × 10^4^	3.34 × 10^3^	3.83 × 10^3^	6.21 × 10^3^	5.35 × 10^3^	4.01 × 10^3^	9.64 × 10^3^
	Ave	3.40 × 10^3^	9.55 × 10^3^	3.30 × 10^4^	3.79 × 10^3^	6.68 × 10^3^	7.81 × 10^3^	6.51 × 10^3^	4.49 × 10^3^	1.22 × 10^4^
	Std	5.27 × 10^1^	1.53 × 10^3^	9.91 × 10^2^	3.06 × 10^2^	4.47 × 10^3^	7.50 × 10^2^	4.77 × 10^2^	1.95 × 10^2^	1.25 × 10^3^
	Rank	1	7	9	2	5	6	4	3	8
F25	Best	1.20 × 10^4^	2.52 × 10^4^	5.14 × 10^4^	1.30 × 10^4^	1.79 × 10^4^	2.96 × 10^4^	2.37 × 10^4^	2.52 × 10^4^	2.45 × 10^4^
	Ave	1.46 × 10^4^	3.12 × 10^4^	5.96 × 10^4^	3.11 × 10^4^	2.43 × 10^4^	3.63 × 10^4^	2.99 × 10^4^	2.87 × 10^4^	2.76 × 10^4^
	Std	1.50 × 10^3^	3.03 × 10^3^	2.62 × 10^3^	9.79 × 10^3^	3.93 × 10^3^	3.20 × 10^3^	2.53 × 10^3^	1.75 × 10^3^	1.63 × 10^3^
	Rank	1	7	9	6	2	8	5	4	3
F26	Best	3.58 × 10^3^	4.72 × 10^3^	7.11 × 10^3^	4.78 × 10^3^	4.01 × 10^3^	4.93 × 10^3^	5.11 × 10^3^	4.30 × 10^3^	4.68 × 10^3^
	Ave	3.80 × 10^3^	5.43 × 10^3^	1.17 × 10^4^	6.97 × 10^3^	4.63 × 10^3^	5.98 × 10^3^	6.07 × 10^3^	5.50 × 10^3^	5.78 × 10^3^
	Std	1.21 × 10^2^	4.63 × 10^2^	1.59 × 10^3^	1.33 × 10^3^	3.97 × 10^2^	9.36 × 10^2^	6.03 × 10^2^	6.65 × 10^2^	4.90 × 10^2^
	Rank	1	3	9	8	2	6	7	4	5
F27	Best	3.41 × 10^3^	1.01 × 10^4^	3.61 × 10^4^	3.67 × 10^3^	5.22 × 10^3^	9.18 × 10^3^	7.58 × 10^3^	4.42 × 10^3^	1.40 × 10^4^
	Ave	3.52 × 10^3^	1.33 × 10^4^	4.10 × 10^4^	5.20 × 10^3^	1.55 × 10^4^	1.15 × 10^4^	9.53 × 10^3^	5.51 × 10^3^	1.61 × 10^4^
	Std	5.00 × 10^1^	1.55 × 10^3^	1.20 × 10^3^	1.73 × 10^3^	8.17 × 10^3^	1.15 × 10^3^	1.04 × 10^3^	3.56 × 10^2^	1.64 × 10^3^
	Rank	1	6	9	2	7	5	4	3	8
F28	Best	6.53 × 10^3^	1.10 × 10^4^	2.72 × 10^5^	9.19 × 10^3^	8.35 × 10^3^	1.36 × 10^4^	1.11 × 10^4^	9.85 × 10^3^	1.10 × 10^4^
	Ave	7.45 × 10^3^	1.64 × 10^4^	3.02 × 10^6^	1.11 × 10^4^	1.08 × 10^4^	1.80 × 10^4^	1.37 × 10^4^	1.11 × 10^4^	1.56 × 10^4^
	Std	5.77 × 10^2^	1.02 × 10^4^	1.77 × 10^6^	1.57 × 10^3^	2.48 × 10^3^	2.61 × 10^3^	1.71 × 10^3^	9.52 × 10^2^	3.30 × 10^3^
	Rank	1	7	9	4	2	8	5	3	6
F29	Best	1.38 × 10^5^	7.17 × 10^8^	4.36 × 10^10^	1.70 × 10^7^	1.23 × 10^7^	6.98 × 10^8^	3.04 × 10^8^	4.08 × 10^7^	1.62 × 10^9^
	Ave	3.30 × 10^5^	3.62 × 10^9^	5.61 × 10^10^	4.61 × 10^7^	1.01 × 10^8^	1.70 × 10^9^	7.75 × 10^8^	1.55 × 10^8^	6.95 × 10^9^
	Std	1.58 × 10^5^	3.05 × 10^9^	3.56 × 10^9^	2.18 × 10^7^	6.06 × 10^7^	9.92 × 10^8^	3.46 × 10^8^	6.50 × 10^7^	3.29 × 10^9^
	Rank	1	7	9	2	3	6	5	4	8

**Table 9:** *p*-value of 8 algorithms on CEC 2017 (Dim = 10).

Function	SCSO	AOA	SSA	DBO	WOA	AO	HHO	GJO
F1	4.62 × 10^−10^	3.02 × 10^−11^	5.09 × 10^−6^	**5.59 × 10^−1^**	3.02 × 10^−11^	3.02 × 10^−11^	3.02 × 10^−11^	3.02 × 10^−11^
F2	2.87 × 10^−10^	3.02 × 10^−11^	3.64 × 10^−8^	1.11 × 10^−4^	3.02 × 10^−11^	4.20 × 10^−10^	**7.84 × 10^−1^**	3.02 × 10^−11^
F3	1.36 × 10^−7^	3.02 × 10^−11^	**2.90 × 10^−1^**	1.25 × 10^−4^	2.23 × 10^−9^	2.62 × 10^−3^	8.66 × 10^−5^	5.57 × 10^−10^
F4	1.11 × 10^−6^	1.09 × 10^−10^	7.38 × 10^−10^	3.99 × 10^−4^	5.46 × 10^−9^	**5.37 × 10^−2^**	1.43 × 10^−8^	1.78 × 10^−4^
F5	2.37 × 10^−10^	3.02 × 10^−11^	3.02 × 10^−11^	1.34 × 10^−5^	3.69 × 10^−11^	2.15 × 10^−10^	3.02 × 10^−11^	5.53 × 10^−8^
F6	5.27 × 10^−5^	3.02 × 10^−11^	3.02 × 10^−11^	**9.47 × 10^−1^**	2.20 × 10^−7^	1.50 × 10^−2^	2.23 × 10^−9^	4.71 × 10^−4^
F7	1.77 × 10^−3^	3.01 × 10^−7^	6.72 × 10^−10^	2.15 × 10^−2^	7.60 × 10^−7^	**1.19 × 10^−1^**	1.68 × 10^−3^	**4.46 × 10^−1^**
F8	8.48 × 10^−9^	3.02 × 10^−11^	3.02 × 10^−11^	7.74 × 10^−6^	3.69 × 10^−11^	5.46 × 10^−9^	4.98 × 10^−11^	4.31 × 10^−8^
F9	3.37 × 10^−4^	3.09 × 10^−6^	2.37 × 10^−10^	3.92 × 10^−2^	1.25 × 10^−4^	**1.33 × 10^−1^**	3.59 × 10^−5^	1.44 × 10^−3^
F10	8.10 × 10^−10^	3.02 × 10^−11^	1.46 × 10^−10^	6.53 × 10^−8^	1.33 × 10^−10^	5.07 × 10^−10^	1.31 × 10^−8^	1.78 × 10^−10^
F11	**5.11 × 10^−1^**	3.02 × 10^−11^	5.83 × 10^−3^	5.61 × 10^−5^	3.34 × 10^−3^	2.05 × 10^−3^	6.91 × 10^−4^	1.95 × 10^−3^
F12	**2.90 × 10^−1^**	**6.41 × 10^−1^**	**8.50 × 10^−2^**	**6.73 × 10^−1^**	**7.98 × 10^−2^**	**7.62 × 10^−1^**	**1.22 × 10^−1^**	**8.07 × 10^−1^**
F13	2.38 × 10^−7^	7.12 × 10^−9^	1.70 × 10^−8^	3.57 × 10^−6^	1.11 × 10^−6^	8.84 × 10^−7^	3.09 × 10^−6^	4.44 × 10^−7^
F14	1.03 × 10^−6^	3.02 × 10^−11^	2.15 × 10^−10^	2.77 × 10^−5^	7.77 × 10^−9^	5.00 × 10^−9^	5.97 × 10^−9^	2.03 × 10^−7^
F15	6.67 × 10^−3^	3.69 × 10^−11^	1.96 × 10^−10^	2.07 × 10^−2^	1.34 × 10^−5^	**7.48 × 10^−2^**	2.60 × 10^−5^	**2.97 × 10^−1^**
F16	1.49 × 10^−6^	2.15 × 10^−10^	3.02 × 10^−11^	8.20 × 10^−7^	2.67 × 10^−9^	2.38 × 10^−7^	1.01 × 10^−8^	4.11 × 10^−7^
F17	**4.55 × 10^−1^**	**6.10 × 10^−1^**	**5.20 × 10^−1^**	**5.20 × 10^−1^**	**3.95 × 10^−1^**	4.06 × 10^−2^	**3.63 × 10^−1^**	2.68 × 10^−6^
F18	6.55 × 10^−4^	1.41 × 10^−9^	1.87 × 10^−5^	4.43 × 10^−3^	1.49 × 10^−6^	5.86 × 10^−6^	5.09 × 10^−6^	2.60 × 10^−5^
F19	1.46 × 10^−10^	1.96 × 10^−10^	3.69 × 10^−11^	1.10 × 10^−8^	7.39 × 10^−11^	4.62 × 10^−10^	1.09 × 10^−10^	1.21 × 10^−10^
F20	6.67 × 10^−3^	1.20 × 10^−8^	5.00 × 10^−9^	**2.40 × 10^−1^**	4.08 × 10^−5^	2.15 × 10^−2^	1.43 × 10^−8^	8.15 × 10^−5^
F21	1.53 × 10^−5^	3.02 × 10^−11^	5.00 × 10^−9^	3.83 × 10^−6^	1.07 × 10^−9^	2.83 × 10^−8^	5.53 × 10^−8^	9.26 × 10^−9^
F22	2.53 × 10^−4^	3.02 × 10^−11^	3.02 × 10^−11^	7.22 × 10^−6^	7.59 × 10^−7^	5.83 × 10^−3^	8.14 × 10^−11^	**5.19 × 10^−2^**
F23	**5.49 × 10^−1^**	1.01 × 10^−8^	1.55 × 10^−9^	**5.40 × 10^−1^**	4.86 × 10^−3^	**9.47 × 10^−1^**	2.13 × 10^−5^	**1.91 × 10^−1^**
F24	6.38 × 10^−3^	3.02 × 10^−11^	1.34 × 10^−5^	3.39 × 10^−2^	3.01 × 10^−7^	3.64 × 10^−2^	4.51 × 10^−2^	**1.05 × 10^−1^**
F25	5.97 × 10^−5^	3.02 × 10^−11^	3.35 × 10^−8^	6.28 × 10^−6^	3.01 × 10^−7^	2.92 × 10^−2^	8.66 × 10^−5^	1.04 × 10^−4^
F26	**1.15 × 10^−1^**	4.08 × 10^−11^	7.39 × 10^−11^	3.64 × 10^−2^	2.38 × 10^−7^	6.77 × 10^−5^	1.55 × 10^−9^	5.57 × 10^−3^
F27	3.50 × 10^−3^	3.02 × 10^−11^	3.67 × 10^−3^	2.32 × 10^−2^	1.47 × 10^−7^	5.57 × 10^−10^	1.43 × 10^−5^	2.00 × 10^−5^
F28	**6.15 × 10^−2^**	6.70 × 10^−11^	4.98 × 10^−11^	2.61 × 10^−2^	4.80 × 10^−7^	**1.54 × 10^−1^**	1.70 × 10^−8^	**1.26 × 10^−1^**
F29	6.67 × 10^−3^	3.02 × 10^−11^	9.07 × 10^−3^	6.10 × 10^−3^	1.64 × 10^−5^	8.12 × 10^−4^	7.04 × 10^−7^	1.70 × 10^−2^

**Table 10:** *p*-value of 8 algorithms on CEC 2017 (Dim = 30).

Function	SCSO	AOA	SSA	DBO	WOA	AO	HHO	GJO
F1	3.02 × 10^−11^	3.02 × 10^−11^	3.02 × 10^−11^	8.29 × 10^−6^	3.02 × 10^−11^	3.02 × 10^−11^	3.02 × 10^−11^	3.02 × 10^−11^
F2	3.02 × 10^−11^	3.02 × 10^−11^	3.02 × 10^−11^	3.02 × 10^−11^	3.02 × 10^−11^	3.02 × 10^−11^	3.02 × 10^−11^	3.02 × 10^−11^
F3	3.02 × 10^−11^	3.02 × 10^−11^	**3.63 × 10^−1^**	4.57 × 10^−9^	3.02 × 10^−11^	3.02 × 10^−11^	9.53 × 10^−7^	3.02 × 10^−11^
F4	2.03 × 10^−9^	3.02 × 10^−11^	3.69 × 10^−11^	3.49 × 10^−9^	8.98 × 10^−11^	6.04 × 10^−7^	1.17 × 10^−9^	2.03 × 10^−7^
F5	3.02 × 10^−11^	3.02 × 10^−11^	3.02 × 10^−11^	3.34 × 10^−11^	3.02 × 10^−11^	3.02 × 10^−11^	3.02 × 10^−11^	4.08 × 10^−11^
F6	2.25 × 10^−4^	3.02 × 10^−11^	3.02 × 10^−11^	**5.40 × 10^−1^**	2.60 × 10^−8^	7.70 × 10^−4^	1.70 × 10^−8^	4.06 × 10^−2^
F7	2.67 × 10^−9^	3.02 × 10^−11^	4.08 × 10^−11^	1.07 × 10^−9^	1.09 × 10^−10^	9.79 × 10^−5^	4.44 × 10^−7^	2.43 × 10^−5^
F8	1.29 × 10^−9^	4.08 × 10^−11^	4.08 × 10^−11^	3.26 × 10^−7^	3.02 × 10^−11^	6.72 × 10^−10^	3.02 × 10^−11^	2.78 × 10^−7^
F9	4.62 × 10^−10^	3.02 × 10^−11^	4.62 × 10^−10^	2.61 × 10^−10^	4.08 × 10^−11^	1.41 × 10^−9^	4.08 × 10^−11^	2.87 × 10^−10^
F10	3.02 × 10^−11^	3.02 × 10^−11^	3.34 × 10^−11^	3.02 × 10^−11^	3.02 × 10^−11^	3.02 × 10^−11^	9.76 × 10^−10^	3.02 × 10^−11^
F11	4.08 × 10^−11^	3.02 × 10^−11^	**5.79 × 10^−1^**	2.13 × 10^−4^	3.02 × 10^−11^	5.49 × 10^−11^	6.12 × 10^−10^	3.02 × 10^−11^
F12	1.36 × 10^−7^	3.02 × 10^−11^	1.41 × 10^−4^	3.01 × 10^−7^	2.03 × 10^−9^	1.69 × 10^−9^	2.39 × 10^−8^	2.61 × 10^−10^
F13	9.07 × 10^−3^	5.49 × 10^−11^	2.53 × 10^−4^	4.36 × 10^−2^	3.35 × 10^−8^	1.56 × 10^−8^	7.60 × 10^−7^	3.83 × 10^−6^
F14	4.07 × 10^−11^	5.07 × 10^−10^	4.18 × 10^−9^	5.96 × 10^−9^	3.02 × 10^−11^	3.33 × 10^−11^	3.33 × 10^−11^	5.49 × 10^−11^
F15	9.83 × 10^−8^	3.02 × 10^−11^	1.29 × 10^−9^	4.94 × 10^−5^	2.87 × 10^−10^	2.83 × 10^−8^	6.72 × 10^−10^	1.68 × 10^−3^
F16	8.56 × 10^−4^	3.34 × 10^−11^	1.46 × 10^−10^	3.01 × 10^−7^	1.70 × 10^−8^	2.77 × 10^−5^	2.16 × 10^−3^	**1.05 × 10^−1^**
F17	**7.73 × 10^−2^**	3.34 × 10^−11^	2.39 × 10^−4^	**2.46 × 10^−1^**	4.31 × 10^−8^	1.19 × 10^−6^	**1.76 × 10^−1^**	**6.57 × 10^−2^**
F18	6.70 × 10^−11^	3.02 × 10^−11^	3.02 × 10^−11^	2.57 × 10^−7^	4.08 × 10^−11^	3.02 × 10^−11^	3.02 × 10^−11^	4.08 × 10^−11^
F19	6.36 × 10^−5^	1.43 × 10^−8^	8.99 × 10^−11^	9.51 × 10^−6^	8.89 × 10^−10^	**5.75 × 10^−2^**	3.52 × 10^−7^	4.43 × 10^−3^
F20	2.87 × 10^−10^	3.02 × 10^−11^	3.02 × 10^−11^	2.87 × 10^−10^	3.02 × 10^−11^	6.53 × 10^−8^	1.21 × 10^−10^	2.32 × 10^−6^
F21	**7.39 × 10^−1^**	3.69 × 10^−11^	6.12 × 10^−10^	**5.01 × 10^−1^**	1.47 × 10^−7^	3.99 × 10^−4^	1.36 × 10^−7^	**8.65 × 10^−1^**
F22	9.92 × 10^−11^	3.02 × 10^−11^	3.02 × 10^−11^	1.21 × 10^−10^	3.02 × 10^−11^	6.07 × 10^−11^	3.02 × 10^−11^	1.01 × 10^−8^
F23	**8.53 × 10^−1^**	3.02 × 10^−11^	3.02 × 10^−11^	9.88 × 10^−3^	6.05 × 10^−7^	**7.06 × 10^−1^**	4.50 × 10^−11^	**1.22 × 10^−1^**
F24	3.02 × 10^−11^	3.02 × 10^−11^	2.43 × 10^−5^	9.82 × 10^−8^	3.02 × 10^−11^	4.97 × 10^−11^	7.37 × 10^−10^	3.02 × 10^−11^
F25	2.13 × 10^−5^	3.02 × 10^−11^	2.57 × 10^−7^	8.35 × 10^−8^	7.39 × 10^−11^	**1.15 × 10^−1^**	1.10 × 10^−8^	2.01 × 10^−4^
F26	5.49 × 10^−11^	3.02 × 10^−11^	3.02 × 10^−11^	1.75 × 10^−5^	3.16 × 10^−10^	6.70 × 10^−11^	4.98 × 10^−11^	4.98 × 10^−11^
F27	3.02 × 10^−11^	3.02 × 10^−11^	**6.31 × 10^−1^**	7.39 × 10^−11^	3.02 × 10^−11^	3.02 × 10^−11^	1.61 × 10^−10^	3.02 × 10^−11^
F28	2.60 × 10^−8^	3.02 × 10^−11^	4.98 × 10^−11^	1.86 × 10^−9^	3.69 × 10^−11^	7.12 × 10^−9^	5.00 × 10^−9^	3.16 × 10^−5^
F29	3.02 × 10^−11^	3.02 × 10^−11^	3.69 × 10^−11^	1.43 × 10^−5^	3.02 × 10^−11^	3.02 × 10^−11^	3.02 × 10^−11^	3.02 × 10^−11^

**Table 11:** *p*-value of 8 algorithms on CEC 2017 (Dim = 50).

Function	SCSO	AOA	SSA	DBO	WOA	AO	HHO	GJO
F1	3.02 × 10^−11^	3.02 × 10^−11^	3.02 × 10^−11^	3.02 × 10^−11^	3.02 × 10^−11^	3.02 × 10^−11^	3.02 × 10^−11^	3.02 × 10^−11^
F2	3.69 × 10^−11^	3.02 × 10^−11^	3.02 × 10^−11^	3.02 × 10^−11^	3.02 × 10^−11^	3.02 × 10^−11^	3.02 × 10^−11^	3.02 × 10^−11^
F3	3.02 × 10^−11^	3.02 × 10^−11^	6.10 × 10^−3^	6.07 × 10^−11^	3.02 × 10^−11^	3.02 × 10^−11^	3.02 × 10^−11^	3.02 × 10^−11^
F4	6.07 × 10^−11^	3.02 × 10^−11^	1.21 × 10^−10^	6.12 × 10^−10^	3.02 × 10^−11^	6.72 × 10^−10^	2.87 × 10^−10^	3.16 × 10^−10^
F5	3.02 × 10^−11^	3.02 × 10^−11^	3.02 × 10^−11^	3.02 × 10^−11^	3.02 × 10^−11^	3.02 × 10^−11^	3.02 × 10^−11^	3.02 × 10^−11^
F6	2.25 × 10^−4^	3.34 × 10^−11^	4.50 × 10^−11^	1.99 × 10^−2^	2.03 × 10^−9^	7.70 × 10^−4^	2.37 × 10^−10^	3.92 × 10^−2^
F7	3.02 × 10^−11^	3.02 × 10^−11^	3.02 × 10^−11^	3.02 × 10^−11^	3.02 × 10^−11^	3.02 × 10^−11^	3.02 × 10^−11^	3.02 × 10^−11^
F8	3.02 × 10^−11^	3.02 × 10^−11^	5.57 × 10^−10^	1.96 × 10^−10^	3.02 × 10^−11^	3.02 × 10^−11^	3.02 × 10^−11^	3.02 × 10^−11^
F9	3.02 × 10^−11^	3.02 × 10^−11^	4.50 × 10^−11^	3.69 × 10^−11^	3.02 × 10^−11^	3.69 × 10^−11^	3.02 × 10^−11^	3.02 × 10^−11^
F10	3.02 × 10^−11^	3.02 × 10^−11^	3.65 × 10^−8^	1.96 × 10^−10^	3.02 × 10^−11^	3.02 × 10^−11^	5.57 × 10^−10^	3.02 × 10^−11^
F11	3.02 × 10^−11^	3.02 × 10^−11^	4.03 × 10^−3^	3.02 × 10^−11^	3.02 × 10^−11^	3.02 × 10^−11^	3.02 × 10^−11^	3.02 × 10^−11^
F12	3.02 × 10^−11^	3.02 × 10^−11^	4.64 × 10^−3^	3.69 × 10^−11^	3.02 × 10^−11^	3.02 × 10^−11^	3.02 × 10^−11^	3.02 × 10^−11^
F13	1.22 × 10^−2^	3.02 × 10^−11^	1.55 × 10^−9^	2.13 × 10^−4^	1.17 × 10^−9^	5.09 × 10^−8^	2.96 × 10^−5^	2.96 × 10^−5^
F14	1.78 × 10^−10^	3.02 × 10^−11^	2.42 × 10^−2^	2.37 × 10^−10^	3.02 × 10^−11^	3.02 × 10^−11^	3.02 × 10^−11^	3.34 × 10^−11^
F15	3.35 × 10^−8^	3.02 × 10^−11^	1.31 × 10^−8^	2.83 × 10^−8^	3.02 × 10^−11^	3.57 × 10^−6^	7.69 × 10^−8^	3.77 × 10^−4^
F16	8.88 × 10^−6^	3.02 × 10^−11^	7.74 × 10^−6^	1.60 × 10^−7^	1.55 × 10^−9^	6.28 × 10^−6^	1.04 × 10^−4^	3.50 × 10^−3^
F17	**1.12 × 10^−1^**	3.02 × 10^−11^	1.49 × 10^−6^	**1.81 × 10^−1^**	2.61 × 10^−10^	2.32 × 10^−6^	**8.07 × 10^−1^**	1.89 × 10^−4^
F18	3.02 × 10^−11^	3.02 × 10^−11^	3.02 × 10^−11^	1.33 × 10^−10^	3.02 × 10^−11^	3.02 × 10^−11^	3.02 × 10^−11^	3.02 × 10^−11^
F19	1.99 × 10^−2^	2.23 × 10^−9^	3.96 × 10^−8^	1.75 × 10^−5^	8.48 × 10^−9^	**1.15 × 10^−1^**	**1.15 × 10^−1^**	**6.31 × 10^−1^**
F20	3.34 × 10^−11^	3.02 × 10^−11^	3.02 × 10^−11^	3.34 × 10^−11^	3.02 × 10^−11^	4.50 × 10^−11^	3.02 × 10^−11^	1.09 × 10^−10^
F21	6.70 × 10^−11^	3.02 × 10^−11^	1.61 × 10^−10^	1.96 × 10^−10^	3.02 × 10^−11^	1.86 × 10^−9^	4.50 × 10^−11^	4.98 × 10^−11^
F22	3.34 × 10^−11^	3.02 × 10^−11^	3.02 × 10^−11^	3.02 × 10^−11^	3.02 × 10^−11^	3.02 × 10^−11^	3.02 × 10^−11^	4.08 × 10^−11^
F23	**7.48 × 10^−2^**	3.02 × 10^−11^	3.02 × 10^−11^	3.08 × 10^−8^	2.61 × 10^−10^	1.17 × 10^−3^	3.02 × 10^−11^	**6.95 × 10^−1^**
F24	3.02 × 10^−11^	3.02 × 10^−11^	8.56 × 10^−4^	2.44 × 10^−9^	3.02 × 10^−11^	3.02 × 10^−11^	3.02 × 10^−11^	3.02 × 10^−11^
F25	7.39 × 10^−11^	3.02 × 10^−11^	7.66 × 10^−5^	4.31 × 10^−8^	3.02 × 10^−11^	1.17 × 10^−3^	8.99 × 10^−11^	6.72 × 10^−10^
F26	3.69 × 10^−11^	3.02 × 10^−11^	3.02 × 10^−11^	8.89 × 10^−10^	4.50 × 10^−11^	3.02 × 10^−11^	3.34 × 10^−11^	3.02 × 10^−11^
F27	3.02 × 10^−11^	3.02 × 10^−11^	3.16 × 10^−5^	3.02 × 10^−11^	3.02 × 10^−11^	3.02 × 10^−11^	3.02 × 10^−11^	3.02 × 10^−11^
F28	4.50 × 10^−11^	3.02 × 10^−11^	4.08 × 10^−11^	1.33 × 10^−10^	3.02 × 10^−11^	3.02 × 10^−11^	8.99 × 10^−11^	8.15 × 10^−11^
F29	3.02 × 10^−11^	3.02 × 10^−11^	3.02 × 10^−11^	1.33 × 10^−10^	3.02 × 10^−11^	3.02 × 10^−11^	3.02 × 10^−11^	3.02 × 10^−11^

**Table 12:** *p*-value of 8 algorithms on CEC 2017 (Dim = 100).

Function	SCSO	AOA	SSA	DBO	WOA	AO	HHO	GJO
F1	3.02 × 10^−11^	3.02 × 10^−11^	**3.79 × 10^−1^**	3.02 × 10^−11^	3.02 × 10^−11^	3.02 × 10^−11^	3.02 × 10^−11^	3.02 × 10^−11^
F2	3.02 × 10^−11^	3.02 × 10^−11^	3.02 × 10^−11^	3.02 × 10^−11^	3.02 × 10^−11^	3.02 × 10^−11^	3.02 × 10^−11^	3.02 × 10^−11^
F3	3.02 × 10^−11^	3.02 × 10^−11^	4.42 × 10^−6^	3.02 × 10^−11^	3.02 × 10^−11^	3.02 × 10^−11^	3.02 × 10^−11^	3.02 × 10^−11^
F4	3.02 × 10^−11^	3.02 × 10^−11^	4.50 × 10^−11^	2.15 × 10^−10^	3.02 × 10^−11^	3.02 × 10^−11^	3.02 × 10^−11^	3.02 × 10^−11^
F5	3.02 × 10^−11^	3.02 × 10^−11^	3.02 × 10^−11^	3.02 × 10^−11^	3.02 × 10^−11^	3.02 × 10^−11^	3.02 × 10^−11^	3.02 × 10^−11^
F6	1.49 × 10^−6^	3.02 × 10^−11^	9.92 × 10^−11^	**3.55 × 10^−1^**	3.02 × 10^−11^	3.20 × 10^−9^	3.02 × 10^−11^	1.95 × 10^−3^
F7	3.34 × 10^−11^	3.02 × 10^−11^	5.49 × 10^−11^	8.99 × 10^−11^	3.02 × 10^−11^	3.02 × 10^−11^	3.02 × 10^−11^	1.46 × 10^−10^
F8	3.69 × 10^−11^	3.02 × 10^−11^	1.69 × 10^−9^	8.99 × 10^−11^	3.02 × 10^−11^	3.02 × 10^−11^	3.02 × 10^−11^	3.02 × 10^−11^
F9	3.02 × 10^−11^	3.02 × 10^−11^	6.07 × 10^−11^	3.02 × 10^−11^	3.02 × 10^−11^	3.02 × 10^−11^	3.02 × 10^−11^	3.02 × 10^−11^
F10	3.02 × 10^−11^	3.02 × 10^−11^	3.02 × 10^−11^	3.02 × 10^−11^	3.02 × 10^−11^	3.02 × 10^−11^	3.02 × 10^−11^	3.02 × 10^−11^
F11	3.02 × 10^−11^	3.02 × 10^−11^	3.34 × 10^−11^	3.02 × 10^−11^	3.02 × 10^−11^	3.02 × 10^−11^	3.02 × 10^−11^	3.02 × 10^−11^
F12	3.02 × 10^−11^	3.02 × 10^−11^	5.53 × 10^−8^	3.02 × 10^−11^	3.02 × 10^−11^	3.02 × 10^−11^	3.02 × 10^−11^	3.02 × 10^−11^
F13	2.03 × 10^−7^	3.02 × 10^−11^	3.52 × 10^−7^	1.36 × 10^−7^	1.46 × 10^−10^	5.49 × 10^−11^	5.61 × 10^−5^	3.47 × 10^−10^
F14	3.02 × 10^−11^	3.02 × 10^−11^	**9.82 × 10^−1^**	9.92 × 10^−11^	3.02 × 10^−11^	3.02 × 10^−11^	3.02 × 10^−11^	3.02 × 10^−11^
F15	3.02 × 10^−11^	3.02 × 10^−11^	2.03 × 10^−9^	6.70 × 10^−11^	3.02 × 10^−11^	3.02 × 10^−11^	3.34 × 10^−11^	3.34 × 10^−11^
F16	3.02 × 10^−11^	3.02 × 10^−11^	3.52 × 10^−7^	3.02 × 10^−11^	3.02 × 10^−11^	3.02 × 10^−11^	3.02 × 10^−11^	3.02 × 10^−11^
F17	9.88 × 10^−3^	3.02 × 10^−11^	5.53 × 10^−8^	2.02 × 10^−8^	3.20 × 10^−9^	4.31 × 10^−8^	2.38 × 10^−3^	1.47 × 10^−7^
F18	3.02 × 10^−11^	3.02 × 10^−11^	3.02 × 10^−11^	3.02 × 10^−11^	3.02 × 10^−11^	3.02 × 10^−11^	3.02 × 10^−11^	3.02 × 10^−11^
F19	4.18 × 10^−9^	3.02 × 10^−11^	8.89 × 10^−10^	4.62 × 10^−10^	4.98 × 10^−11^	4.35 × 10^−5^	3.20 × 10^−9^	2.68 × 10^−6^
F20	3.02 × 10^−11^	3.02 × 10^−11^	3.02 × 10^−11^	3.02 × 10^−11^	3.02 × 10^−11^	3.02 × 10^−11^	3.02 × 10^−11^	3.02 × 10^−11^
F21	3.02 × 10^−11^	3.02 × 10^−11^	1.96 × 10^−10^	3.02 × 10^−11^	3.02 × 10^−11^	3.02 × 10^−11^	3.02 × 10^−11^	3.02 × 10^−11^
F22	3.02 × 10^−11^	3.02 × 10^−11^	3.02 × 10^−11^	3.02 × 10^−11^	3.02 × 10^−11^	3.02 × 10^−11^	3.02 × 10^−11^	3.02 × 10^−11^
F23	3.02 × 10^−11^	3.02 × 10^−11^	3.02 × 10^−11^	3.02 × 10^−11^	3.02 × 10^−11^	3.02 × 10^−11^	3.02 × 10^−11^	3.02 × 10^−11^
F24	3.02 × 10^−11^	3.02 × 10^−11^	5.53 × 10^−8^	3.02 × 10^−11^	3.02 × 10^−11^	3.02 × 10^−11^	3.02 × 10^−11^	3.02 × 10^−11^
F25	3.02 × 10^−11^	3.02 × 10^−11^	2.02 × 10^−8^	3.02 × 10^−11^	3.02 × 10^−11^	3.02 × 10^−11^	3.02 × 10^−11^	3.02 × 10^−11^
F26	3.02 × 10^−11^	3.02 × 10^−11^	3.02 × 10^−11^	4.08 × 10^−11^	3.02 × 10^−11^	3.02 × 10^−11^	3.02 × 10^−11^	3.02 × 10^−11^
F27	3.02 × 10^−11^	3.02 × 10^−11^	3.02 × 10^−11^	3.02 × 10^−11^	3.02 × 10^−11^	3.02 × 10^−11^	3.02 × 10^−11^	3.02 × 10^−11^
F28	3.02 × 10^−11^	3.02 × 10^−11^	3.02 × 10^−11^	9.92 × 10^−11^	3.02 × 10^−11^	3.02 × 10^−11^	3.02 × 10^−11^	3.02 × 10^−11^
F29	3.02 × 10^−11^	3.02 × 10^−11^	3.02 × 10^−11^	3.02 × 10^−11^	3.02 × 10^−11^	3.02 × 10^−11^	3.02 × 10^−11^	3.02 × 10^−11^

**Table 13:** Wilcoxon rank sum test statistical results.

IMSCSO VS.	CEC2017	CEC2017	CEC2017	CEC2017
	(Dim = 10)	(Dim = 30)	(Dim = 50)	(Dim = 100)
SCSO	23/6/0	26/3/0	27/2/0	29/0/0
AOA	27/2/0	29/0/0	29/0/0	29/0/0
SSA	24/3/2	22/3/4	26/0/3	24/2/3
DBO	22/6/1	26/3/0	28/1/0	28/1/0
WOA	27/2/0	29/0/0	29/0/0	29/0/0
AOA	22/7/0	25/3/1	28/1/0	29/0/0
HHO	25/3/1	28/1/0	27/2/0	29/0/0
GJO	21/7/1	25/4/0	27/2/0	29/0/0
Overall (+/=/−)	**191**/36/5	**210**/17/5	**221**/8/3	**226**/3/3

**Table 14:** Friedman mean rank test.

Suites	CEC 2017							
Dimensions	10		30		50		100	
Algorithms	Ave.	Overall	Ave.	Overall	Ave.	Overall	Ave.	Overall
	Rank	Rank	Rank	Rank	Rank	Rank	Rank	Rank
IMSCSO	**1.45**	**1**	**1.31**	**1**	**1.10**	**1**	**1.10**	**1**
SCSO	4.41	4	4.55	4	5.31	7	5.03	6
AOA	8.14	9	8.66	9	8.41	9	8.14	9
SSA	7.03	8	5.10	7	4.52	3	4.17	2
DBO	3.55	2	4.41	3	4.69	5	4.79	4
WOA	6.62	7	7.03	8	7.07	8	7.07	8
AOA	3.79	3	4.24	2	4.66	4	4.93	5
HHO	5.45	6	4.83	6	4.21	2	4.17	2
GJO	4.55	5	4.86	5	5.03	6	5.59	7

## Data Availability

The data presented in this study are available on request from the corresponding author.

## References

[1] Wu G., Pedrycz W., Suganthan P.N., Mallipeddi R. (2015). A variable reduction strategy for evolutionary algorithms handling equality constraints. Appl. Soft Comput. J..

[2] Tang A.D., Han T., Zhou H., Xie L. (2021). An improved equilibrium optimizer with application in unmanned aerial vehicle path planning. Sensors.

[3] Huang C., Zhao K. (2018). Three Dimensional Path Planning of UAV with Improved Ant Lion Optimizer. Dianzi Yu Xinxi Xuebao/J. Electron. Inf. Technol..

[4] Liu Q., Li N., Jia H., Qi Q., Abualigah L. (2022). Modified Remora Optimization Algorithm for Global Optimization and Multilevel Thresholding Image Segmentation. Mathematics.

[5] Liu Q., Li N., Jia H., Qi Q., Abualigah L. (2023). A chimp-inspired remora optimization algorithm for multilevel thresholding image segmentation using cross entropy. Artif. Intell. Rev..

[6] Jia H., Zhang W., Zheng R., Wang S., Leng X., Cao N. (2022). Ensemble mutation slime mould algorithm with restart mechanism for feature selection. Int. J. Intell. Syst..

[7] Zouache D., Got A., Alarabiat D., Abualigah L., Talbi E.G. (2024). A novel multi-objective wrapper-based feature selection method using quantum-inspired and swarm intelligence techniques. Multimed. Tools Appl..

[8] Jia H., Sun K. (2021). Improved barnacles mating optimizer algorithm for feature selection and support vector machine optimization. Pattern Anal. Appl..

[9] Got A., Zouache D., Moussaoui A., Abualigah L., Alsayat A. (2024). Improved Manta Ray Foraging Optimizer-based SVM for Feature Selection Problems: A Medical Case Study. J. Bionic Eng..

[10] Li Y., Han T., Zhao H., Gao H. (2019). An adaptive whale optimization algorithm using gaussian distribution strategies and its application in heterogeneous ucavs task allocation. IEEE Access.

[11] Wang X., Zhao H., Han T., Zhou H., Li C. (2019). A grey wolf optimizer using Gaussian estimation of distribution and its application in the multi-UAV multi-target urban tracking problem. Appl. Soft Comput. J..

[12] Mamoudan M.M., Jafari A., Mohammadnazari Z., Nasiri M.M., Yazdani M. (2023). Hybrid machine learning-metaheuristic model for sustainable agri-food production and supply chain planning under water scarcity. Resour. Environ. Sustain..

[13] Gorji S.A. (2023). Challenges and opportunities in green hydrogen supply chain through metaheuristic optimization. J. Comput. Des. Eng..

[14] Yazdani M., Kabirifar K., Haghani M. (2024). Optimising post-disaster waste collection by a deep learning-enhanced differential evolution approach. Eng. Appl. Artif. Intell..

[15] Mittal N., Singh U., Salgotra R., Sohi B.S. (2019). An energy efficient stable clustering approach using fuzzy extended grey wolf optimization algorithm for WSNs. Wirel. Netw..

[16] Mittal N., Singh U., Salgotra R., Sohi B.S. (2018). A boolean spider monkey optimization based energy efficient clustering approach for WSNs. Wirel. Netw..

[17] Salgotra R., Singh U. (2018). A novel bat flower pollination algorithm for synthesis of linear antenna arrays. Neural Comput. Appl..

[18] Singh U., Salgotra R. (2019). Synthesis of Linear Antenna Arrays Using Enhanced Firefly Algorithm. Arab. J. Sci. Eng..

[19] Khalili-Fard A., Parsaee S., Bakhshi A., Yazdani M., Aghsami A., Rabbani M. (2024). Multi-objective optimization of closed-loop supply chains to achieve sustainable development goals in uncertain environments. Eng. Appl. Artif. Intell..

[20] Yazdani M., Haghani M. (2023). Elderly people evacuation planning in response to extreme flood events using optimisation-based decision-making systems: A case study in western Sydney, Australia. Knowl.-Based Syst..

[21] Holland J.H. (1992). Adaptation in Natural and Artificial Systems.

[22] Sarker R.A., Elsayed S.M., Ray T. (2014). Differential evolution with dynamic parameters selection for optimization problems. IEEE Trans. Evol. Comput..

[23] Fogel D.B. (1993). Applying evolutionary programming to selected traveling salesman problems. Cybern. Syst..

[24] Beyer H.-G., Schwefel H.-P. (2002). Evolution strategies—A comprehensive introduction. Nat. Comput..

[25] Yang X. (2010). Nature-Inspired Metaheuristic Algorithms.

[26] Rashedi E., Nezamabadi-pour H., Saryazdi S. (2009). GSA: A Gravitational Search Algorithm. Inf. Sci..

[27] Mirjalili S. (2016). SCA: A Sine Cosine Algorithm for solving optimization problems. Knowl.-Based Syst..

[28] Mirjalili S., Mirjalili S.M., Hatamlou A. (2016). Multi-Verse Optimizer: A nature-inspired algorithm for global optimization. Neural Comput. Appl..

[29] Rao R.V., Savsani V.J., Vakharia D.P. (2011). Teaching-learning-based optimization: A novel method for constrained mechanical design optimization problems. CAD Comput. Aided Des..

[30] Bayzidi H., Talatahari S., Saraee M., Lamarche C.P. (2021). Social Network Search for Solving Engineering Optimization Problems. Comput. Intell. Neurosci..

[31] Zhang Y., Jin Z. (2020). Group teaching optimization algorithm: A novel metaheuristic method for solving global optimization problems. Expert Syst. Appl..

[32] Kennedy J., Eberhart R. Particle swarm optimization. Proceedings of the IEEE International Conference on Neural Networks—Conference Proceedings.

[33] Dorigo M., Di Caro G. Ant colony optimization: A new meta-heuristic. Proceedings of the 1999 Congress on Evolutionary Computation, CEC 1999.

[34] Mirjalili S., Lewis A. (2016). The Whale Optimization Algorithm. Adv. Eng. Softw..

[35] Mirjalili S., Mirjalili S.M., Lewis A. (2014). Grey Wolf Optimizer. Adv. Eng. Softw..

[36] Abualigah L., Elaziz M.A., Sumari P., Geem Z.W., Gandomi A.H. (2022). Reptile Search Algorithm (RSA): A nature-inspired meta-heuristic optimizer. Expert Syst. Appl..

[37] Agushaka J.O., Ezugwu A.E., Abualigah L. (2022). Dwarf Mongoose Optimization Algorithm. Comput. Methods Appl. Mech. Eng..

[38] Xie L., Han T., Zhou H., Zhang Z.-R., Han B., Tang A. (2021). Tuna Swarm Optimization: A Novel Swarm-Based Metaheuristic Algorithm for Global Optimization. Comput. Intell. Neurosci..

[39] Yazdani M., Jolai F. (2016). Lion Optimization Algorithm (LOA): A nature-inspired metaheuristic algorithm. J. Comput. Des. Eng..

[40] Salgotra R., Singh U. (2019). The naked mole-rat algorithm. Neural Comput. Appl..

[41] Seyyedabbasi A., Kiani F. (2023). Sand Cat swarm optimization: A nature-inspired algorithm to solve global optimization problems. Eng. Comput..

[42] Wolpert D.H., Macready W.G. (1997). No free lunch theorems for optimization. IEEE Trans. Evol. Comput..

[43] Seyyedabbasi A. (2023). A reinforcement learning-based metaheuristic algorithm for solving global optimization problems. Adv. Eng. Softw..

[44] Wang X., Liu Q., Zhang L. (2023). An Adaptive Sand Cat Swarm Algorithm Based on Cauchy Mutation and Optimal Neighborhood Disturbance Strategy. Biomimetics.

[45] Wu D., Rao H., Wen C., Jia H., Liu Q., Abualigah L. (2022). Modified Sand Cat Swarm Optimization Algorithm for Solving Constrained Engineering Optimization Problems. Mathematics.

[46] Li Y., Wang G. (2022). Sand Cat Swarm Optimization Based on Stochastic Variation with Elite Collaboration. IEEE Access.

[47] Qtaish A., Albashish D., Braik M., Alshammari M.T., Alreshidi A., Alreshidi E.J. (2023). Memory-Based Sand Cat Swarm Optimization for Feature Selection in Medical Diagnosis. Electronics.

[48] Abualigah L., Diabat A., Mirjalili S., Abd Elaziz M., Gandomi A.H. (2021). The Arithmetic Optimization Algorithm. Comput. Methods Appl. Mech. Eng..

[49] Mirjalili S., Gandomi A.H., Mirjalili S.Z., Saremi S., Faris H., Mirjalili S.M. (2017). Salp Swarm Algorithm: A bio-inspired optimizer for engineering design problems. Adv. Eng. Softw..

[50] Xue J., Shen B. (2023). Dung beetle optimizer: A new meta-heuristic algorithm for global optimization. J. Supercomput..

[51] Abualigah L., Yousri D., Abd Elaziz M., Ewees A.A., Al-qaness M.A.A., Gandomi A.H. (2021). Aquila Optimizer: A novel meta-heuristic optimization algorithm. Comput. Ind. Eng..

[52] Heidari A.A., Mirjalili S., Faris H., Aljarah I., Mafarja M., Chen H. (2019). Harris hawks optimization: Algorithm and applications. Future Gener. Comput. Syst..

[53] Chopra N., Mohsin Ansari M. (2022). Golden jackal optimization: A novel nature-inspired optimizer for engineering applications. Expert Syst. Appl..

[54] Kahraman H.T., Aras S., Gedikli E. (2020). Fitness-distance balance (FDB): A new selection method for meta-heuristic search algorithms. Knowl.-Based Syst..

[55] Salgotra R., Singh U., Saha S., Nagar A. New Improved SALSHADE-cnEpSin Algorithm with Adaptive Parameters. Proceedings of the 2019 IEEE Congress on Evolutionary Computation, CEC 2019.

[56] Li Y., Han T., Zhou H., Tang S., Zhao H. (2022). A novel adaptive L-SHADE algorithm and its application in UAV swarm resource configuration problem. Inf. Sci..

[57] Salgotra R., Singh S., Singh U., Saha S., Gandomi A.H. Hybridizing Cuckoo Search with Naked Mole-rat Algorithm: Adapting for CEC 2017 and CEC 2021 Test Suites. Proceedings of the 2021 IEEE Symposium Series on Computational Intelligence, SSCI 2021.

[58] Salgotra R., Singh U., Saha S. Improved Cuckoo Search with Better Search Capabilities for Solving CEC2017 Benchmark Problems. Proceedings of the 2018 IEEE Congress on Evolutionary Computation, CEC 2018.

[59] Li Y., Han T., Zhou H., Wei Y., Wang Y., Tan M., Huang C. (2023). APSM-jSO: A novel jSO variant with an adaptive parameter selection mechanism and a new external archive updating mechanism. Swarm Evol. Comput..

[60] Li Y., Han T., Wang X., Zhou H., Tang S., Huang C., Han B. (2023). MjSO: A modified differential evolution with a probability selection mechanism and a directed mutation strategy. Swarm Evol. Comput..

